# Transcriptomic Data Analyses Reveal That Sow Fertility-Related lincRNA *NORFA* Is Essential for the Normal States and Functions of Granulosa Cells

**DOI:** 10.3389/fcell.2021.610553

**Published:** 2021-02-23

**Authors:** Xing Du, Qiqi Li, Liu Yang, Qiang Zeng, Siqi Wang, Qifa Li

**Affiliations:** College of Animal Science and Technology, Nanjing Agricultural University, Nanjing, China

**Keywords:** *NORFA*, pig, granulosa cells, RNA-seq, sow fertility

## Abstract

*NORFA*, the first lincRNA associated with sow fertility, has been shown to control granulosa cell (GC) functions and follicular atresia. However, the underlying mechanism is not fully understood. In this study, RNA-seq was performed and we noticed that inhibition of *NORFA* led to dramatic transcriptomic alterations in porcine GCs. A total of 1,272 differentially expressed transcripts were identified, including 1167 DEmRNAs and 105 DEmiRNAs. Furthermore, protein–protein interaction, gene-pathway function, and TF–miRNA–mRNA regulatory networks were established and yielded four regulatory modules with multiple hub genes, such as *AR*, *ATG5*, *BAK1*, *CENPE*, *NR5A1*, *NFIX*, *WNT5B*, *ssc-miR-27b*, and *ssc-miR-126*. Functional assessment showed that these hub DEGs were mainly enriched in TGF-β, PI3K-Akt, FoxO, Wnt, MAPK, and ubiquitin pathways that are essential for GC states (apoptosis and proliferation) and functions (hormone secretion). *In vitro*, we also found that knockdown of *NORFA* in porcine GCs significantly induced cell apoptosis, impaired cell viability, and suppressed 17β-estradiol (E2) synthesis. Notably, four candidate genes for sow reproductive traits (*INHBA*, *NCOA1*, *TGF*β*-1*, and *TGFBR2*) were also identified as potential targets of *NORFA*. These findings present a panoramic view of the transcriptome in *NORFA*-reduced GCs, highlighting that *NORFA*, a candidate lincRNA for sow fertility, is crucial for the normal states and functions of GCs.

## Introduction

In mammalian ovaries, follicular development is a crucial biological process for follicle maturation, ovulation, and female fertility (Botta et al., 2017). However, more than 99% of follicles undergo atresia during each stage of follicular development (Kaipia and Hsueh, 1997; Liu et al., 2014). As the main threat to female ovarian function, severe follicular atresia leads to follicular development arrest, premature follicles failure (PFF), and even infertility (Meng et al., 2017; Yeung et al., 2017). It is generally believed that the fate of follicles is mainly determined by the state of granulosa cells (GCs) (Yu et al., 2004; Matsuda-Minehata et al., 2006). Increasing evidence has also suggested that GC apoptosis and non-apoptotic programmed cell death are the main causes of follicular atresia (Shen et al., 2017; Zhang et al., 2018). Recent studies have suggested that GC apoptosis is mediated by a complicated regulatory network consisting of multiple crucial factors that can be divided into two categories, exogenous factors (i.e., environmental factors and oxidative stress) (Li et al., 2019a; Spears et al., 2019) and endogenous factors (i.e., steroid hormones, cytokines, and epigenetic regulators) (Yang et al., 2017; Li et al., 2018a, 2019b). In the first two decades of this century, a bunch of microRNAs (miRNAs) and circRNAs have been identified by using high-throughput sequencing technology and further proved to be involved in the regulation of follicular development, especially for GC states and functions (Du et al., 2016, 2018; Guo et al., 2019). However, the roles of long non-coding RNAs (lncRNAs) in follicular atresia and GC apoptosis have not been fully characterized.

LncRNAs are a class of RNA transcripts that are more than 200 nucleotides (nt) in length and lacking of the protein-coding potential or open reading frame (ORF) (Canzio et al., 2019). Unlike the protein-coding RNAs, lncRNAs are relatively unstable, are less evolutionary conserved, have lower abundance, and have species- or tissue-specific expression patterns, which results in the large unknown about the roles, functions, and regulatory mechanisms of lncRNAs (Ransohoff et al., 2018). Compared to the mature miRNAs, which are mainly located in the cytoplasm and inhibit target gene expression at the post-transcriptional level, the location and regulatory mechanisms of lncRNAs are more complex (Kopp and Mendell, 2018). It has been reported that lncRNAs can be located in both cytoplasm and nucleus, which are involved in the control of multiple crucial biological processes by regulating gene expression in *cis* or in *trans* manners (Engreitz et al., 2016; Uszczynska-Ratajczak et al., 2018). Although thousands of lncRNAs in human or other organisms have been identified by high-throughput sequencing technology, only a few of them are well characterized (Liu et al., 2018; Stojic et al., 2020). Notably, only several lncRNAs including *lncRNA-Neat1* (Nakagawa et al., 2014), *lncRNA-HUPCOS* (Che et al., 2020), and *lncRNA-PVT1* (Liu et al., 2020) have been reported to be involved in the regulation of ovarian functions, such as steroid hormone secretion and corpus luteum formation. However, the role of lncRNAs in regulating follicular development, GC state, and functions remain unknown.

It has been well documented that 17β-estradiol (E2), the principal form of estrogens, is produced by adrenals and ovarian GCs and crucial for estrus cycle, ovarian development, follicular maturity, and ovulation (Baerwald et al., 2012; Herbison, 2020). E2 secretion is the most important function of female ovarian GCs, which also feedback regulates the transcriptome and the states (such as proliferation and apoptosis) of GCs in an autocrine manner (Ciesiolka et al., 2017; Liu et al., 2019). In recent years, E2 synthesis in female ovarian GCs has been proved to be regulated by a bunch of epigenetic regulation factors including miRNAs, circRNAs, and lncRNAs (Li et al., 2018a; Huang et al., 2020). For lncRNAs, only *lncRNA-Gm2044* (Hu et al., 2019) and *lncRNA-SRA* (Li et al., 2018b) have been proved to influence E2 synthesis and secretion in mammalian ovarian GCs through regulating the expression of *Cyp19a1*, but which are all studied in mice. However, it is still unclear whether lncRNAs can regulate E2 synthesis in porcine ovarian GCs. In our previous study, we have identified a pig-specific novel functional lincRNA (*NORFA*) in porcine GCs, which is 739 nt in length with two exons and transcript from pig chromosome 1, and further demonstrate that *NORFA* is the first candidate lincRNA for sow fertility through inhibiting follicular atresia (Du et al., 2020). However, little is known about the role of *NORFA* in porcine GCs. This study aims to comprehensively investigate the transcriptomic alteration of porcine GCs induced by knockdown of *NORFA* and further primarily detect the role of *NORFA* in the regulation of the normal states and functions of porcine GCs.

## Materials and Methods

### Animal Ethics

A total of 50 healthy and sexually mature commercial large white sows (average 180 days, and average mass 110 kg) randomly selected from Zhunshun Biological Technology Co. (Nanjing, China) were involved in this study for bilateral ovaries collection and GC isolation. The studied sows were healthy, unstimulated, and taken care of by animal welfare. All the experiments involving animals in this research were approved and supervised by the Animal Ethics Committee of Nanjing Agricultural University, Nanjing, Jiangsu, China [SYXK (Su) 2015-0656].

### Cell Culture and Treatment

The sows were slaughtered according to the animal ethics and the fresh ovaries were collected and placed in a flask filled with 37°C normal saline, which were then sent back to the laboratory within 1 h. Porcine GCs were derived from 3- to 5-mm non-atretic follicles by using the syringes with 22-gauge needles and cultured into six-well plates filled with DEME/F12 medium (#11320033, Invitrogen) with 10% fetal bovine serum (FBS, #30044333, Gibco) and 1% penicillin/streptomycin, which were then placed in a 37°C incubator with humidified atmosphere and 5% CO_2_ for 24 h as described previously (Du et al., 2018). For siRNA, miRNA, and plasmid transfection, Lipofectamine 3000 reagent (#L13778-150, Invitrogen) was used according to the manufacturer’s instructions. The oligonucleotide sequences used in this study were synthesized by GenePharma (Shanghai, China) and listed as follows: *NORFA*-specific small interfering RNA (siNORFA), 5′-CAG ACA GAU GUG GAU GAA UTT-3′ (Sense), 5′-UGU GGU UGA UGU UGU UGG CTT-3′ (Anti-sense); NC-siRNA (siNC), 5′-UUC UCC GAA CGU GUC ACG UTT-3′ (Sense), 5′-ACG UGA CAC GUU CGG AGA ATT-3′ (Anti-sense). pcDNA3.1-*NFIX*, the eukaryotic expression vector for porcine *NFIX*, was constructed in our previous study (Du et al., 2020). For pan-caspase inhibitor treatment, porcine GCs were seeded into six-well cell culture plates for 36 h, and the medium was then replaced with serum-free DMEM/F12 medium for 12 h. Subsequently, Z-VAD-FMK (#MK3270-1MG, MKBio, Shanghai, China), a well-known pan-caspase inhibitor, was added to the medium to a final concentration of 50 μM. All the experiments were repeated in triplicate.

### RNA Isolation, Library Preparation, and Sequencing

After transfection for 24 h, total RNA from porcine GCs were isolated and purified by using TRIzol reagent (#15596018, Invitrogen). The quantity and quality of the purified RNA were detected by a NanoDrop 3000 spectrophotometer (Agilent Technologies, United States). The degradation and contamination of the total RNA were estimated by running on a 1.0% agarose gel, and an Agilent 2100 Bioanalyzer (Agilent Technologies, United States) was used to detect the integrity of each sample. Each group has three independent samples, and the total RNA from the same treatment group were diluted and equally mixed to avoid the intra-species differences. Thus, a total of two RNA libraries were established for sequencing. Before the preparation for RNA-seq, the transfection efficiency and knockdown efficiency of siNORFA in porcine GCs were detected and validated. Subsequently, the cDNA libraries for sequencing were prepared as previously described (Du et al., 2019). Briefly, after removing rRNA, total RNA was broken into 100- to 150-nt fragments and cDNA was synthesized and purified by using QIAquick PCR Purification kit (#28104, QIAGEN), which was subsequently sent to Biomarker Technologies Co. Ltd. (Beijing, China) for sequencing. Paired-end sequencing of 150 bases length was performed by using an Illumina HiSeq3000. The total clean tags were checked and genome mapping was performed by HISAT2 software (Kim et al., 2015); the reads and sequences were obtained with the background of *Sus Scrofa* RefSeq (*Sscorfa* 11.1) database. Subsequently, the known and novel transcripts were identified. The full raw transcriptome sequencing data sets have been submitted to sequence read archive (SRA) database of NCBI (SRR11793932, SRR11793933, SRR11793934, and SRR11793935, Bioproject ID: PRJNA632987).

### Identification of the Differentially Expressed Transcripts

The total clean tags were extracted and low-quality reads were removed by using Perl scripts designed by Biomarker Technologies Co. Ltd. (Beijing, China). After quantile normalization, sequencing data of each gene in all samples were normalized as fragments per kilobase of transcript sequence per million mapped reads (FPKM). The expression level changes of each transcript were transformed with log2, and the significance was adjusted to the control for false discovery rate (FDR). For miRNAs, their expression levels were corrected and normalized by using the TPM algorithm on an R-studio software. Differentially expressed mRNA (DEmRNAs) and miRNAs (DEmiRNAs) were identified as a result of comparison the transcriptomes between control group and *NORFA*-inhibited group, and detected by DESeq R package v1.10.1 (Anders and Huber, 2010) and IEDG6 tool (Romualdi et al., 2003) according to their corresponding protocols, respectively. The significant differentially expressed transcripts were identified with a cutoff criteria of adjusted FDR < 0.05 and |Log_2_(fold change)| ≥ 0.59 (or |fold change| > 1.5).

### Functional Analysis of DEmRNAs and DEmiRNAs

To identify the potential functions and roles of DEmRNAs, and their involved biological processes, Gene Ontology (GO) analyses were performed by using the Database for Annotation, Visualization and Integrated Discovery^1^ (DAVID v6.8). To identify the signaling pathways that DEmRNAs were mainly enriched in, Kyoto Encyclopedia of Genes and Genomes (KEGG) analyses were performed by using the KOBAS online analysis tool^2^. To analyze the functional annotation and miRNA–pathway interaction of DEmiRNAs, the targets of DEmiRNAs were firstly identified by using miRWalk 3.0, TarBase, and TaregetScan online miRNAs software. Subsequently, DIANA-miRPath v 3.0 database^3^ was used to analyze the functions of DEmiRNAs according the methods as previously described (Vlachos et al., 2015). The significance of *P* < 0.05 and enrichment score ≥2 were set as the thresholds for both DEmRNAs and DEmiRNAs. To further evaluate the alteration trend of the significant enriched GO terms in *NORFA*-reduced porcine GCs, *z* score was calculated based on the expression pattern of the DEGs, which were enriched in each GO term.

### Protein–Protein Interaction (PPI) Network Construction

To construct the effective *NORFA*-mediated PPI network, all the function-known DEmRNA-encoded proteins were selected, and the network was established as previously described (Du et al., 2019). Briefly, all the interactions (validated and predicted) between DEG-encoded proteins were analyzed and generated by using STRING v11.0 online statistical analysis software^4^ (Szklarczyk et al., 2019) with the basic settings: the interacted protein amount ≥1 and the minimum required interaction score ≥0.7 [0–1]. The PPI network was subsequently visualized by using Cytoscape v3.7.2 software (Shannon et al., 2003). The nodes with higher degrees (top 5%) were considered as hub genes and identified using Cytohubba function. The different modules in the PPI network were identified by Cytoscape software MCODE package function.

### DEmiRNA–DEmRNA Regulatory Network Construction

To construct the *NORFA*-mediated DEmiRNA–DEmRNA regulatory network, the identified targets of DEmiRNAs were firstly obtained using DIANA-TarBase database^5^. The common genes between DEmRNAs and validated target genes of DEmiRNAs were considered as significant differentially expressed target genes. Furthermore, DEmiRNAs and common DEmRNA targets with opposite expression patterns were chosen for DEmiRNA–DEmRNA regulatory network construction using Cytoscape v3.7.2 software. To further assess the potential functions of this miRNA–mRNA regulatory network, DAVID database was used to analyze the significant enriched GO annotation and KEGG pathways. Hub miRNAs were defined as the miRNA nodes that had higher degree (top 10%) in the DEmiRNA–DEmRNA regulatory network.

### Potential DETF–DEmiRNA Regulatory Network Construction

To construct the potential *NORFA*-mediated DETF (differentially expressed transcription factor)–DEmiRNA regulatory network, the candidate promoters of DEmiRNAs were analyzed by Promoter 2.0 prediction server^6^, and the potential transcription factors within these promoters were identified by using JASPAR online database^7^, which is an open-access database of curated, non-redundant TF binding profiles. The DETFs were considered as the common molecules between DEmRNAs and candidate TFs that target the DEmiRNAs mentioned above. The similarity of binding motif ≥0.9 [0–1] and the interacted DEmiRNA amount ≥1 were set as the thresholds for DETFs. The DETF–DEmiRNA regulatory network was constructed and visualized by using Cytoscape v3.7.2 software. The TFs and miRNAs with higher degrees (top 5%) were considered as hub nodes in the DETF–DEmiRNA regulatory network.

### Quantitative Real-Time PCR (qRT-PCR) Validation for RNA-Seq Analysis

Quantitative real-time PCR (qRT-PCR) was performed to confirm the accuracy of RNA-seq analysis and detect the expression pattern of crucial genes. In brief, 1 μg of total RNA derived from porcine GCs in the control group or the siNORFA treatment group was reverse-transcribed into cDNA by using HiScript III RT SuperMix for qPCR with gDNA wiper (#R323-01, Vazyme Biotech Co., Ltd., Nanjing, China) according to the manufacturer’s instructions. Twelve DEmRNAs and six DEmiRNAs, which are crucial for cell states and functions, were selected for validation. qRT-PCR was conducted on an ABI 7500 System (Applied Biosystems) with three independent biological repeats by using AceQ qPCR SYBR Green Master Mix (#Q111-03, Vazyme Biotech Co., Ltd., Nanjing, China), and the original experimental data were analyzed using 2^–ΔΔCT^ method. *GAPDH* and *U6* were selected to normalize the expression levels of coding and non-coding genes, respectively. Each group has three independent samples and qRT-PCR has been performed in triplicate. The primers used are listed in Supplementary Table 1.

### Western Blotting

Porcine GCs were lysed with cold RIPA buffer and total proteins were collected. The concentration of each protein sample was detected by BCA method (#P0012, Beyotime Biotechnology). Equal amounts of total protein (15 μg) were loaded and separated on 4–20% SDS-PAGE gel to measure the expression levels of interested proteins; Western blotting assays were performed as described previously (Du et al., 2020). The primary antibodies used in this study were anti-Caspase3/cleaved-Caspase3 (#19677-1-AP, Proteintech, 1:1,000), anti-PCNA (#13110, Cell Signaling Technology, 1:1,000), anti-MCM2 (#4007s, Cell Signaling Technology, 1:1,000), anti-NFIX (#F3250, Affinity, 1:1,000), and anti-GAPDH (#TA802519, ORIGENE, 1:2,000). Corresponding HRP-conjugated secondary antibodies were purchased from Sangon Biotech (Shanghai, China) and diluted in 0.25% BSA/TBST. The protein level of GAPDH was detected as an internal control.

### Morphometric Analysis

In order to analyze the effects of knockdown of *NORFA* on the morphology of porcine GCs, morphometric analysis was performed after 24-h transfection with siNORFA. The high-solution images of porcine GCs were obtained from Odyssey Imaging System (LI-COR Biosciences). The detection area was randomly selected, and the analysis was performed with three independent samples per group.

### Caspase3 Activity Detection Assay

Caspase3 Activity Assay kit (#C1115, Beyotime Biotechnology) was used to detect the activity of Caspase3 in control or *NORFA*-reduced porcine GCs according to the manufacturer’s instruction. In brief, GCs were collected and lysed with 100 μl of cold lysis buffer for 15 min. After 16,000 *g* centrifugation for 15 min at 4°C, 50 μl of supernatant was transferred into a new tube and incubated with 10 μl of Ac-DEVD-*p*NA (2 mM) at 37°C for 2 h. Then, the activity of Caspase3 in *NORFA*-reduced porcine GCs was measured and indicated by the absorbance at 405 nm.

### Cell Viability Assay and Apoptosis Detection

Cell viability was performed with TransDetect cell counting kit-8 (CCK-8) (#FC101, Transgen) according to the manufacturer’s instructions. In brief, 2,000 porcine GCs were seeded in 96-well cell culture plates. After treatment for the indicated time, 10 μl of CCK solution was added to the medium for 2 h and the absorbance was detected at 450 nm with a microplate reader. The cell viability was calculated with the following equation: (OD_treatment_ − OD_control_)/OD_control_. Each group has six independent replicates.

For cell apoptosis measurement, Annexin V-FITC/PI Apoptosis Detection kit (#A211, Vazyme, China) was purchased and performed according to the manufacturer’s instruction. Briefly, 20,000 cells were collected and washed with cold PBS twice, and subsequently dyed with Annexin V-FITC and PI in the dark room for 10 min, and finally they were sorted by FACS on a cell counting machine. The apoptosis rate was determined using the following equation: (cell number in the right quadrants)/(total cell number).

### E2 Measurement

After transfection for 48 h, the E2 secretion levels within the cell culture medium were analyzed using a 17β-estradiol detection kit (enzyme-linked immunosorbent assay, ELISA) (#AR E-8800, Beijing North Institute of Biotechnology Co., Ltd.) according to the manufacturer’s instruments. The sensitivity of detection kit was 0.1 pg/ml, and each group has three independent samples.

### Statistical Analysis

Statistical analyses were performed by using SPSS v20.0 (SPSS, IL, United States) and GraphPad Prism v8.0 (GraphPad Software, CA, United States). Figures were made by GraphPad Prism and Rstudio. The data in qRT-PCR, Western blotting, Caspase3 activity assay, and ELISA experiments were presented as mean ± S.E.M., and the significance was calculated by a two-tailed Student’s *t* test. *P* value <0.05 was considered as statistically significant.

## Results

### Transcriptome Sequencing of Porcine GCs

**FIGURE 1:**
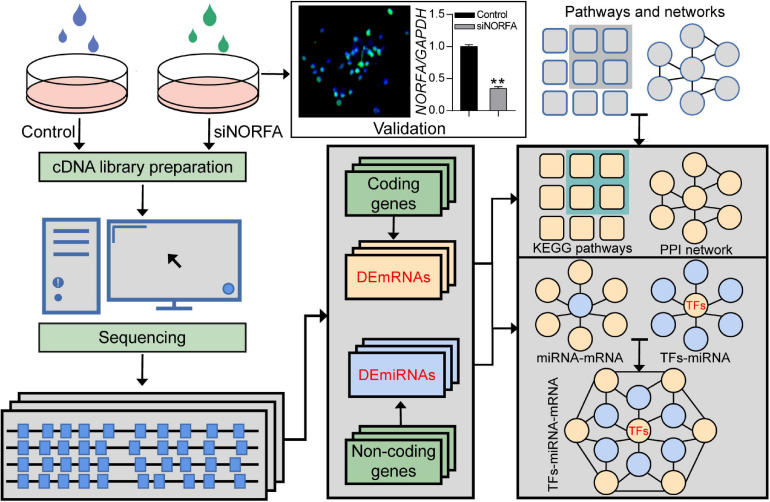
The strategy for identification of *NORFA*-mediated TF–miRNA–mRNA regulatory network in porcine GCs. Flow diagram representing the strategy for DEmRNAs and DEmiRNAs detection by using RNA-seq and the subsequent assessment of their function as well as the construction of the regulatory network in *NORFA*-inhibited porcine GCs.

In the current study, a high-throughput RNA sequencing strategy was designed to identify the crucial RNA molecules and core pathways involved in the responses of porcine GCs to the knockdown of *NORFA* (Figure 1). Briefly, porcine GCs were cultured for 48 h and then transfected with siNORFA as indicated. The transfection efficiency and knockdown efficiency of siNORFA in porcine GCs were detected. Then, the cells were collected for transcriptome sequencing experiment. After filtering out the low-quality reads and removing the adaptor sequences, about 38.22 Gb clean data (paired-end, average 63,806,779 reads per sample) with Q30(%) > 93.83% were obtained. Using a two-iteration mapping strategy, approximately 96.25% of the total clean reads were mapped to the *Sus scrofa* genome assembly 11.1 (range from 96.14 to 96.35%) with HISAT2. Transcriptomes were assembled using StringTie (Pertea et al., 2016), and transcripts from each sample were merged into a consensus transcriptome model. Furthermore, the differentially expressed RNA molecules including mRNAs and miRNAs were identified, and their potential functions were assessed. Besides, according to multiple bioinformatic analyses, the *NORFA*-mediated PPI network and miRNA–mRNA and TF–miRNA regulatory networks were also established to comprehensively understand the roles of *NORFA* in porcine GCs.

### Identification and Characterization of the DEmRNAs in *NORFA*-Inhibited Porcine GCs

**FIGURE 2:**
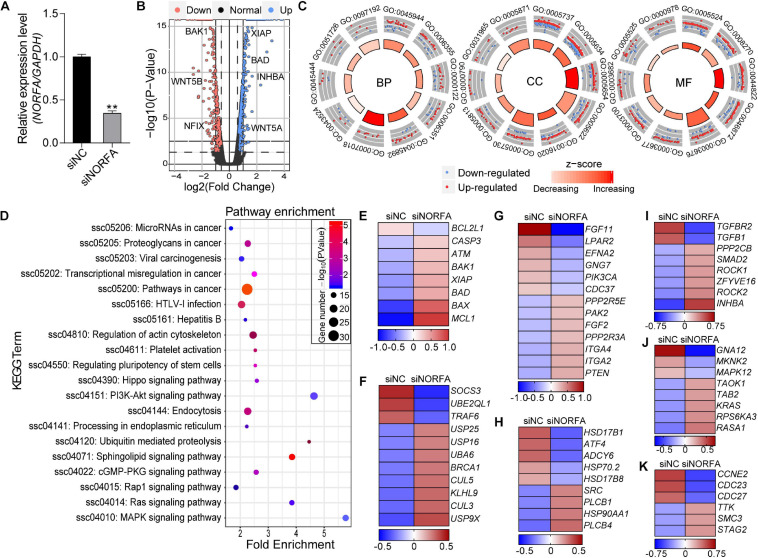
Identification and functional assessment of DEmRNAs in *NORFA*-reduced porcine GCs. **(A)** The expression level of *NORFA* in porcine GCs after treatment with siNORFA was detected by qRT-PCR (*n* = 3). **(B)** Volcano plot representing the expression profile of DEmRNAs in *NORFA*-reduced porcine GCs with the criteria of FDR < 0.05 and |Log_2_(fold change)| ≥ 0.59. Blue and pink points indicate up- and down-regulated genes, while the normal genes are labeled in black. Several crucial DEmRNAs were also indicated in the volcano plot. **(C)** Gene Ontology (GO) analyses of the DEmRNAs in *NORFA*-reduced porcine GCs and top 10 GO terms in BP (biological process), CC (biological process), and MF (molecular function) categories were represented. Blue points in each term indicated down-regulated DEmRNAs, while red points indicated up-regulated DEmRNAs. The regulation trend of each term was estimated according to the *z* score, which ranges from light red (decreasing) to dark red (increasing). **(D)** KEGG analyses of the DEmRNAs in *NORFA*-reduced porcine GCs and the significant enriched KEGG terms were also presented. **(E–K)** Heatmaps showing the expression patterns of DEmRNAs in KEGG enriched pathways, apoptosis signaling pathway **(E)**, ubiquitin signaling pathway **(F)**, PI3K-Akt signaling pathway **(G)**, estrogen signaling pathway **(H)**, TGF-β signaling pathway **(I)**, MAPK signaling pathway **(J)**, and cell cycle signaling pathway **(K)**. The color scale of each heatmap ranges from blue (low expression) to red (high expression).

**FIGURE 3:**
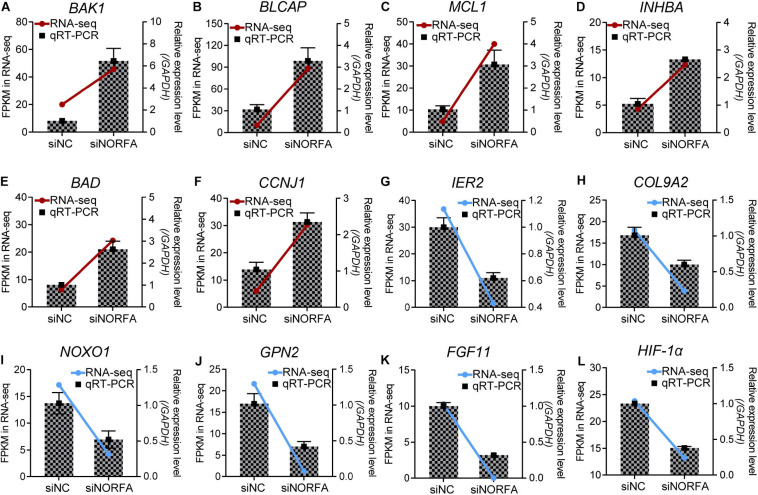
Multiple crucial genes were influenced by *NORFA* in porcine GCs. A total of 12 key DEmRNAs that are essential for cell states and functions were selected, and their expression patterns in porcine GCs treated with siNORFA were validated by qRT-PCR (*n* = 3). **(A–F)** Six up-regulated DEmRNAs and **(G–L)** six down-regulated DEmRNAs. The color lines showing the expression patterns of DEmRNAs in RNA-seq, which refers to the left *y-*axis (red and blue indicate up- and down-regulated). The columns represent qRT-PCR results by referring to the right *y*-axis (mean ± S.E.M.; *n* = 3).

Based on the data from RNA-seq, a total of 17,503 genes were detected. Among them, 1,167 DEmRNAs including 965 function-known genes and 202 function-unknown genes were identified in *NORFA*-reduced porcine GCs compared to the control group (Figures 2A,B and Supplementary Table 2) with the criteria of FDR < 0.05 and |Log_2_(fold change)| ≥ 0.59. The expression patterns of DEmRNAs were analyzed by generating DEmRNA-involved heatmaps and showed that 787 DEmRNAs are up-regulated and 380 are down-regulated (Supplementary Figure 1A). According to the fold change, the top 10 most up- and down-regulated DEmRNAs are listed in Table 1. The most up- and down-regulated DEmRNA are *LOC100522201* and *Sus scrofa_newgene_194813*. To further assess the functions of these DEmRNAs, 965 function-known DEmRNAs were chosen for GO analyses and a total of 88 significantly altered GO terms (*P* < 0.05) were identified, including 45 biological process (BP) terms, 23 cell component (CC) terms, and 20 molecular function (MF) terms (Supplementary Table 3). As shown in Figure 2C, the top 10 terms of each GO category and their alteration trend in response to the knockdown of *NORFA* were presented. The most enriched GO term in BP, CC, and MF categories are “positive regulation of transcription from RNA polymerase II promoter,” “cytoplasm,” and “ATP binding.” Besides, KEGG pathway enrichment analyses were performed and 43 significant enriched pathways (*P* < 0.05), including PI3K-Akt, TGF-β, MAPK, ubiquitin-mediated proteolysis, and estrogen signaling pathway, which mainly associate with cell functions and states, were identified (Figure 2D and Supplementary Table 4). The expression patterns of DEmRNAs involved in the seven most enriched KEGG pathways were detected and listed in Figures 2E–K. Furthermore, 12 key DEmRNAs (6 up- and 6 down-regulated) that play essential roles in the mammalian ovarian GCs were selected for qRT-PCR detection, and results showed that the alteration of their expression patterns in *NORFA*-reduced porcine GCs were extremely similar to the sequencing data (Figure 3), indicating high accuracy of the sequencing data, and also suggesting that *NORFA* influences the expression of multiple crucial genes in porcine GCs at transcription level.

**TABLE 1:** Top 10 down- and up-regulated DEmRNAs in *NORFA*-inhibited porcine GCs.

**Gene ID**	**Gene symbol**	**Chr.^1^**	**Log_2_FC**	**FDR**	**Regulated**
Sus scrofa_newgene_194813	–	1	−7.066	3.16E−33	Down
gene19996	*FGF11*	12	−6.955	7.94E−31	Down
gene24136	*LOC110256914*	15	−6.815	1.58E−29	Down
gene29811	*LOC110258925*	X	−6.241	2.51E−27	Down
Sus scrofa_newgene_30420	*–*	9	−5.488	3.16E−26	Down
gene3831	*IER2*	2	−5.320	5.01E−14	Down
Sus scrofa_newgene_32920	*–*	13	−5.298	3.16E−12	Down
gene13673	*LOC110261743*	7	−5.246	3.98E−13	Down
Sus scrofa_newgene_92712	*–*	6	−5.220	5.01E−35	Down
gene12983	*LOC100158121*	7	−5.198	6.31E−23	Down
Sus scrofa_newgene_206428	*–*	1	5.176	3.98E−26	Up
gene13051	*LOC110261670*	7	5.223	3.16E−27	Up
gene8084	*MCL1*	4	5.276	2.51E−28	Up
Sus scrofa_newgene_194856	*–*	5	5.367	2.00E−29	Up
Sus scrofa_newgene_192505	*–*	5	5.773	1.58E−30	Up
gene21112	*P2RY1*	13	6.213	1.26E−23	Up
gene14415	*LOC100152619*	7	6.219	1.26E−23	Up
Sus scrofa_newgene_209873	*–*	X	6.262	1.00E−30	Up
gene8105	*LOC100154181*	4	8.146	7.94E−33	Up
gene16625	*LOC100522201*	9	8.828	5.01E−34	Up

*^1^Chr. indicates chromosome in porcine genome.*

### *NORFA*-Mediated PPI Network Construction and Module Identification

**FIGURE 4:**
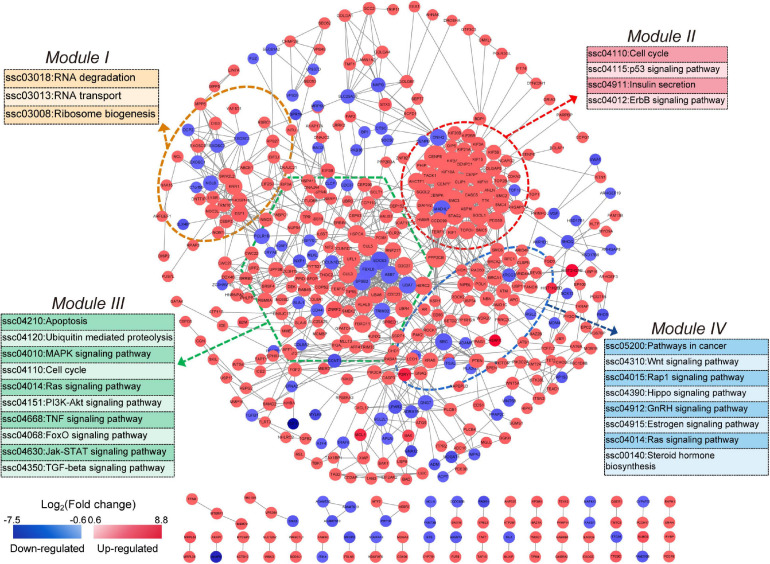
Identification of the *NORFA*-mediated protein–protein interaction network. The *NORFA*-mediated protein–protein interaction (PPI) network was constructed based on the DEmRNAs obtained from RNA-seq results. A total of 462 protein-coding DEmRNAs (nodes) were identified in the PPI network. Color scale depicts the expression pattern of DEmRNAs and ranges from blue to red (red indicates up-regulated and blue indicates down-regulated). Degrees are shown as the size of the nodes. The dotted areas indicate four significant modules (module I in orange, module II in red, module III in green, and module IV in blue). The most enriched signaling pathways (*P* < 0.05) represented by the four modules are depicted.

To construct the *NORFA*-mediated PPI network, 965 protein-coding DEmRNAs were chosen for interaction analysis by using STRING online database and the Cytoscape v3.7.2 visualization tool. As shown in Figure 4, a total of 462 nodes (295 up- and 167 down-regulated DEmRNAs) and 1,334 edges exist in the *NORFA*-mediated PPI network. After analysis, the enrichment *P* value is less than 1.00E−16, the average node degree (AND) is 3.27, and the average local clustering coefficient (ALCC) is 0.537, indicating that the *NORFA*-mediated PPI network has relatively high reliability. Also, 25 nodes with high degree (top 5%) were identified as hub genes (Supplementary Table 5), among which *CENPE*, *SMC3*, *KIF18A*, *PPP2CB*, *CENPF*, *KIF2A*, *CUL5*, *KIF11*, *CUL3*, and *CASC5* are the top 10 nodes with high degree (average degree ≥ 21). Besides, qRT-PCR was performed and results showed that the expression patterns of these 10 hub genes in *NORFA*-inhibited porcine GCs were consistent with the RNA-seq data (Supplementary Figure 2). Furthermore, four significant enriched modules (termed modules I, II, III, and IV) were identified in the PPI network. The significant enriched signaling pathways in which DEmRNAs within these four modules were participating in were identified by KEGG analyses and showed that these modules were mainly associated with the pathways involved in cell cycle, proliferation, apoptosis, steroid hormone synthesis, and ubiquitin-mediated proteolysis (Figure 4), suggesting that *NORFA* has an important role in regulating multiple biological processes in porcine GC.

### Identification and Functional Analyses of the DEmiRNAs in *NORFA*-Reduced Porcine GCs

**FIGURE 5:**
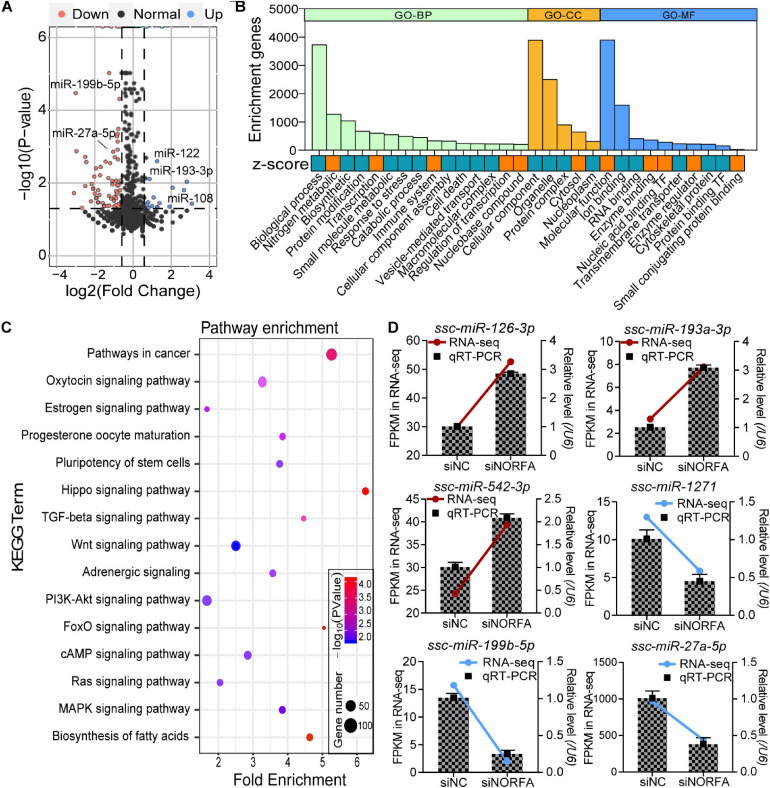
Identification and functional annotation of DEmiRNAs in porcine GCs treated with *NORFA*-siRNA. **(A)** Volcano plot showing the expression profile of DEmiRNAs in *NORFA*-reduced porcine GCs with the following criteria FDR < 0.05 and |Log_2_(fold change)| ≥ 0.59. Up- and down-regulated genes are labeled in blue and pink; the black points indicate normal genes. Several important DEmiRNAs were also indicated in the volcano plot. **(B)** Gene Ontology analyses of DEmiRNAs in porcine GCs treated with *NORFA*-siRNA. The columns in green, yellow, and blue indicate the enriched terms of BP (biological process), CC (cellular components), and MF (molecular function) categories. The significant GO terms with their full names were listed below and their expression patterns were estimated by *z* score, which were labeled in orange (up-regulated) and blue (down-regulated). **(C)** KEGG pathway enrichment analyses of DEmiRNAs in *NORFA*-inhibited porcine GCs and significant KEGG terms were also presented. **(D)** Six DEmiRNAs (three up- and three down-regulated) that are crucial for ovarian GCs were selected, and their expression patterns in porcine GCs were validated by qRT-PCR. The color lines depict the expression patterns of DEmiRNAs determined by RNA-seq, which refers to the left *y*-axis (red and blue indicate up- and down-regulated). The bars represent the results of qRT-PCR by referring to the right *y*-axis (mean ± S.E.M.; *n* = 3).

In addition to DEmRNAs, 105 differentially expressed miRNAs (DEmiRNAs, 23 up- and 82 down-regulated) were also identified by RNA-seq with the following criteria of FDR < 0.05 and |fold change| ≥ 1.5, and several crucial miRNAs for ovarian GCs, such as miR-122 and miR-27a-5p, were also differentially expressed (Figure 5A and Supplementary Table 6). According to fold change, the expression patterns of DEmiRNAs were detected by constructing DEmiRNA-mediated heatmaps (Supplementary Figure 1B). After analysis, the top 10 up- and down-regulated DEmiRNAs are listed in Table 2, among which, novel-miR-619 and novel-miR-318 are the most up- and down-regulated DEmiRNA, respectively. To further assess the potential roles and functions of the 105 DEmiRNAs in *NORFA*-reduced porcine GCs, the identified targets of DEmiRNAs were analyzed and GO analyses were conducted. A total of 104 significant GO terms (*P* < 0.05, micro T-threshold > 0.8) including three categories (88 GO terms in BP, 6 GO terms in CC, and 10 GO terms in MF) were identified by using the miRPath v3.0 database (Supplementary Table 7), and top 30 GO terms were presented in Figure 5B. In the BP category, “cellular nitrogen compound metabolic process” is the most significant enriched GO term, while “organelle” and “ion binding” are the most significant enriched GO terms in the CC and MF categories, respectively. Furthermore, KEGG analyses showed that the DEmiRNAs were mainly enriched in 45 signaling pathways (*P* < 0.05, Supplementary Table 8), which are involved in regulating cell growth, proliferation, apoptosis, death, and functions, such as TGF-β, FoxO, PI3K-Akt, cAMP, insulin, hippo, and Wnt signaling pathways (Figure 5C). Furthermore, six important DEmiRNAs were selected for qRT-PCR validation, and the results were highly consistent with RNA-seq data (Figure 5D). These observations not only indicate a high accuracy in our RNA-seq data but also demonstrate that multiple crucial miRNAs in porcine GCs are regulated by *NORFA*.

**TABLE 2:** Top 10 down- and up-regulated DEmiRNAs in *NORFA*-reduced porcine GCs.

**miRNAs**	**Mature sequence (5′–3′)**	**Log_2_FC**	**FDR**	**Regulated**
novel-miR-318	AGCCAGGUGGAUGCGGACGAGC	−22.41	4.91E−03	Down
novel-miR-122	CAGGGCAUCCCUGUAGGAGCU	−22.14	1.15E−02	Down
novel-miR-118	CCUCUGUUCACUCCCUGGAGCU	−21.82	2.67E−02	Down
novel-miR-263	UGGAGGAGUCCUGGGCCUCUCUG	−21.63	3.92E−02	Down
novel-miR-74	CGUGUGCUGCCCAGAGGCUGG	−3.09	1.88E−02	Down
ssc-miR-199b	CCCAGUGUUUAGACUAUCUGUU	−3.01	3.36E−05	Down
novel-miR-155	UGCAAUUGUGAUCUGAUCUCAGC	−2.97	1.33E−03	Down
novel-miR-77	UAGUUGCUGUGGGAAGUGAGC	−2.68	4.83E−02	Down
ssc-miR-9851	UGGCACCAGCACUGGCGGUGGC	−2.51	1.21E−02	Down
novel-miR-616	UUGCAAGCAACACUCUGUGGCAGAU	−2.51	1.90E−03	Down
novel-miR-517	ACCGGCCCGGGGCUGCCUCCA	1.27	4.66E−02	Up
ssc-miR-193a	AACUGGCCUACAAAGUCCCAGU	1.27	4.66E−02	Up
novel-miR-205	UAAUACUGCCGGGUAAUGAUGGA	1.39	3.57E−02	Up
ssc-miR-122	UGGAGUGUGACAAUGGUGUUUGU	1.51	2.30E−05	Up
novel-miR-59	GGGCAAGCCUGCGGAGGUGUGG	1.91	4.46E−02	Up
novel-miR-591	CUAUACAACUUACUACUUUCCC	2.08	1.34E−02	Up
novel-miR-256	UCCAGGGUGGAAAAGAGCUUG	2.66	1.57E−02	Up
novel-miR-290	GAUUGGGAACACUGGAGAACU	2.81	9.12E−03	Up
novel-miR-108	UUGGGGGACGAGAGGCUGUG	3.08	3.70E−02	Up
novel-miR-619	UAUGUGGGACGGUAAACCAUU	22.90	1.17E−04	Up

### Construction and Functional Annotation of *NORFA*-Mediated DEmiRNA–DEmRNA Regulatory Network

**FIGURE 6:**
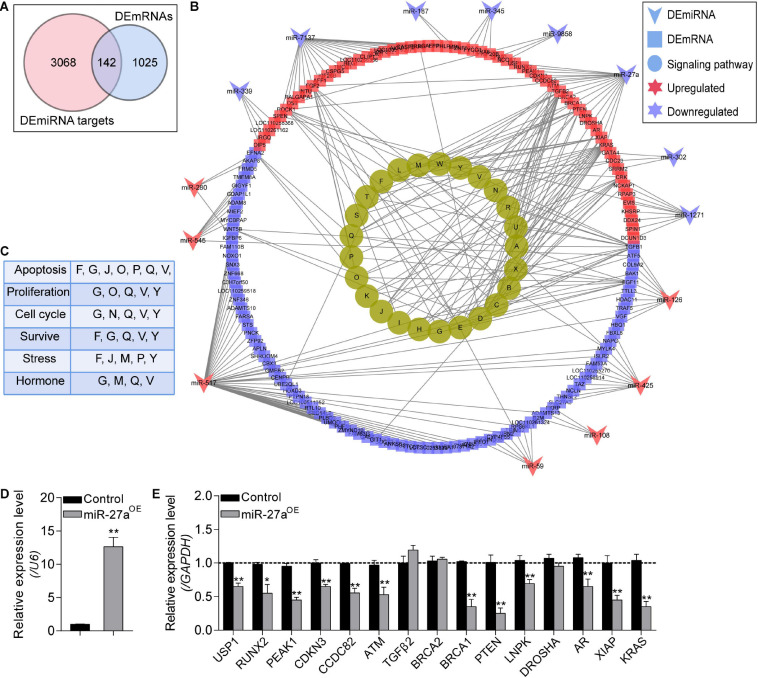
Identification of the *NORFA*-mediated DEmiRNA–DEmRNA network. **(A)** Venn diagram showing the common genes that simultaneously belong to the DEmRNAs and the validated target genes of DEmiRNAs. **(B)** DEmiRNA–DEmRNA pathway network. Arrows indicate DEmiRNAs, squares indicate DEmRNAs, and circles represent the most significant signaling pathways (*P* < 0.05). The colors depict the changes of the expression patterns (red and purple indicate up- and down-regulated). The identified KEGG pathways were as follows: A, pathways in cancer; B, regulation of actin cytoskeleton; C, microRNAs in cancer; D, HTLV-I infection; E, proteoglycans in cancer; F, ubiquitin mediated proteolysis; G, MAPK signaling pathway; H, renal cell carcinoma; I, melanoma; J, FoxO signaling pathway; K, chronic myeloid leukemia; L, toxoplasmosis; M, focal adhesion; N, cell cycle; O, Rap1 signaling pathway; P, Ras signaling pathway; Q, PI3K-Akt signaling pathway; R, leishmaniasis; S, pancreatic cancer; T, hepatitis B; U, colorectal cancer; V, TGF-β signaling pathway; W, small cell lung cancer; X, prostate cancer; Y, NF-kappa B signaling pathway. **(C)** The *NORFA*-mediated pathway–function interactions were analyzed. **(D)** The expression level of miR-27a in porcine GCs treated with miR-27a mimics was detected by qRT-PCR (*n* = 3). **(E)** The interactions between miR-27a and its potential targets within the DEmiRNA–DEmRNA regulatory network were measured by qRT-PCR (*n* = 3). Data in **(D)** and **(E)** were presented as mean ± S.E.M. with three independent replicates, and *P* values were calculated by a two-tailed Student’s *t* test. *P < 0.05 and **P < 0.01.

To construct the *NORFA*-mediated DEmiRNA–DEmRNA regulatory network, we first analyzed the targets of DEmiRNAs and a total of 3,210 validated targets were identified, including 142 common genes with DEmRNAs in the sequencing data (Figure 6A). With the interaction between DEmiRNAs and their target DEmRNAs (Supplementary Table 9), the DEmiRNA–DEmRNA regulatory network containing 185 nodes and 274 edges was established (Figure 6B). After analysis, 15 DEmiRNAs (8 down- and 7 up-regulated) and 142 DEmRNAs (83 down- and 59 up-regulated) exist in the network. Among them, novel-miR-517 and ssc-miR-7137 with high degree were considered as hub miRNAs. Besides, functional assessment of the DEmRNAs in the network showed that they are mainly enriched in PI3K-Akt, TGF-β, FoxO, MAPK, Ras, Rap1, and ubiquitin signaling pathways (Figure 6B). In addition, pathway-function co-expression patterns were analyzed to estimate the effects of this regulatory network on porcine GCs and showed that the *NORFA*-mediated DEmiRNA–DEmRNA regulatory axes play important roles in regulating the states (apoptosis, proliferation, and survival) and functions (response to stress and hormone secretion) of porcine GCs (Figure 6C). Furthermore, the interactions between miR-27a and 15 target DEmRNAs in this network were also validated by qRT-PCR. As shown in Figures 6D,E, overexpression of miR-27a dramatically inhibited the mRNA levels of 12 targets in porcine GCs but does not affect the expression of *BRCA2*, *TGF*β*2*, and *DROSHA*, indicating high reliability of *NORFA*-mediated DEmiRNA–DEmRNA regulatory network.

### Construction of the Potential DETF–DEmiRNA Interaction Network in *NORFA*-Reduced Porcine GCs

**FIGURE 7:**
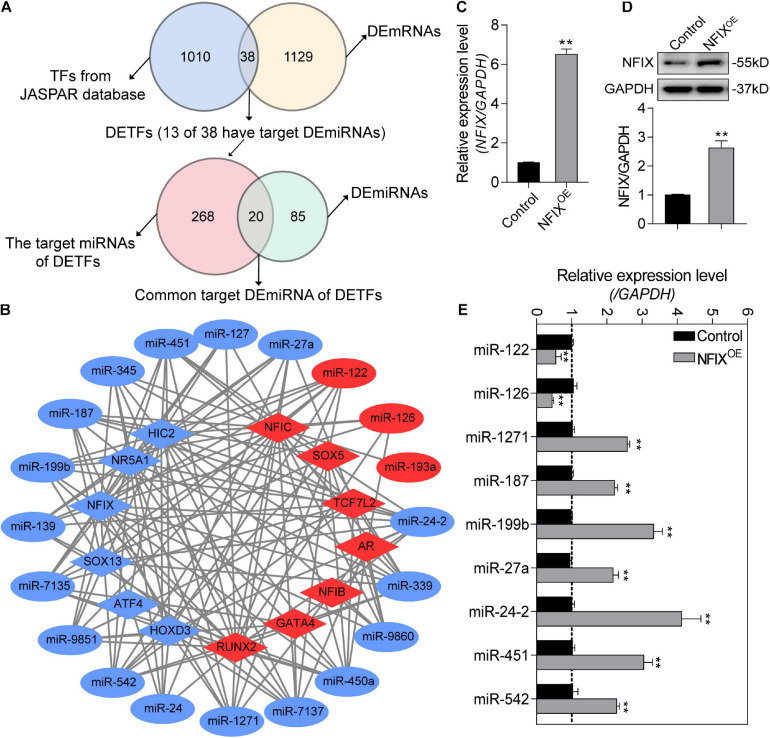
Identification of the potential *NORFA*-mediated DETF–DEmiRNA regulatory network. **(A)** Venn diagrams showing the identified DETFs and their target DEmiRNAs. **(B)** The potential DETF–DEmiRNA regulatory network was constructed. Circles indicate DEmiRNAs and diamonds indicate DETFs. The color depicts the expression patterns (red indicates up-regulated and blue indicates down-regulated). **(C,D)** The expression of NFIX at both mRNA level **(C)** and protein level **(D)** in NFIX overexpressed porcine GCs were detected by qRT-PCR and Western blotting (*n* = 3). **(E)** The expression patterns of the potential NFIX-regulated miRNAs in NFIX overexpressed porcine GCs were identified by qRT-PCR (*n* = 3). Data in **(C–E)** were shown as mean ± S.E.M. with three independent replicates. *P* values were calculated by a two-tailed Student’s *t* test. **P < 0.01.

To further investigate the *NORFA*-mediated DETF–DEmiRNA regulatory network, 38 DETFs were first identified from 1,167 DEmRNAs, and only 13 of which have been reported to be able to target 20 common DEmiRNAs between 105 DEmiRNAs and 288 DETF–target miRNAs (Figure 7A). As shown in Figure 7B, the *NORFA*-mediated DETF–DEmiRNA regulatory network containing 33 nodes and 151 edges was constructed with the interaction between 13 DETFs (6 down- and 7 up-regulated) and 20 common DEmiRNAs (17 down- and 3 up-regulated), which has been also listed in Supplementary Table 10. After analysis, NFIC and miR-24-2 with the highest degrees were considered as the hub DETF and DEmiRNA in this regulatory network, respectively. To detect the accuracy of the network, the expression levels of nine DEmiRNAs (the potential targets of NFIX) in *NFIX* overexpressed porcine GCs were determined and the qRT-PCR results showed that their expression patterns were similar to the RNA-seq data (Figures 7C–E), indicating that *NORFA* regulates the expression of miRNAs in porcine GCs partially in a TF-dependent manner.

### Knockdown of *NORFA* Impairs the Normal States and Functions of Porcine GCs

**FIGURE 8:**
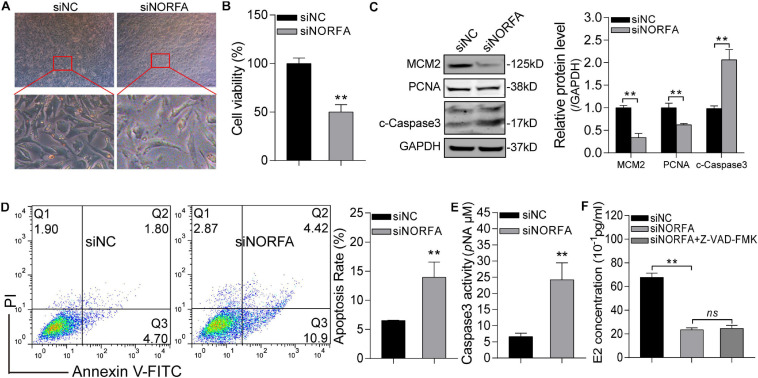
Knockdown of *NORFA* impairs the normal states and functions of porcine GCs. **(A)** The morphological features of control and *NORFA*-reduced porcine GCs were observed and recorded by a stereomicroscope. **(B)** Cell viability in control and siNORFA treatment group was measured by CCK-8 reagent at the 48-h point. **(C)** The effects of *NORFA* reduction on the protein level of MCM2, PCNA, and cleaved-Caspase3 (c-Caspase3) in porcine GCs were determined by Western blotting. **(D,E)** Porcine GCs were transfected with siNORFA for 48 h, and then cell apoptosis rate **(D)** and Caspase3 activity **(E)** were measured. **(F)** The concentration of E2 in porcine GCs after treatment with siNORFA or siNORFA with 50 μM Z-VAD-FMK (a pan-caspase inhibitor) was detected. Data were represented as mean ± S.E.M., and *P* values were calculated by a two-tailed Student’s *t* test with three independent replicates. **P < 0.01 and ns indicates no significance.

Based on the transcriptomic data and bioinformatic analysis results, we hypothesized that *NORFA* is essential for the normal states and functions of porcine GCs. To address this, the effects of siNORFA on porcine GCs were identified. Morphometric analysis showed that shrink and jagged edges occurred in *NORFA*-reduced porcine GCs (Figure 8A), indicating that knockdown of *NORFA* destroyed the membrane integrity of porcine GCs and led to low cell viability, which was further proved by cell viability assay (Figure 8B) and proliferation-associated protein level detection (Figure 8C). Furthermore, siNORFA treatment significantly induced porcine GC apoptosis and increased Caspase3 activity and cleaved-Caspase3 protein level (Figures 8C–E). As the most important function of GCs, E2 secretion is crucial for follicular development. Here, we also detected the E2 synthesis in *NORFA*-inhibited porcine GCs and validated that knockdown of *NORFA* dramatically inhibited the E2 secretion (Figure 8G), and the inhibitory efforts cannot be rescued by Z-VAD-FMK (a pan-caspase inhibitor), indicating that knockdown of *NORFA* suppresses E2 synthesis not through inducing porcine GC apoptosis. Taken together, these data demonstrate that *NORFA* is essential for maintaining the normal states and functions of porcine GCs.

## Discussion

Follicular development is influenced by a complex regulatory network consisting of steroid hormones, growth factors, and epigenetic factors (Cao et al., 2018), which are not fully identified. With the continuous development and improvement of high-throughput sequencing technology, the mono-mics sequencing technology (genomics, transcriptomics, proteomics, and metabolomics) and multi-omics joint analysis have been well improved (Rappoport and Shamir, 2019). In recent years, a large number of studies have analyzed the alterations in different omics during follicular development by using high-throughput sequencing technology. For example, genomics and transcriptomics have been applied to detect the crucial genes and RNA molecules (i.e., mRNAs and miRNAs) at different stages of follicular development in human, pig, and goat (Zou et al., 2020). RNA-seq has become an innovative method for quantifying the transcriptome signatures, and high-throughput RNA-seq has been considered as a standard strategy and an indispensable tool for transcriptome-wide alteration in different tissues, species, and conditions (Stark et al., 2019). In the current study, we have employed an RNA-seq strategy to characterize the transcriptome profiles in *NORFA*-reduced porcine GCs and attempted to identify the crucial downstream targets of *NORFA*.

In the present study, the transcriptome-wide analysis was performed and 1,272 differentially expressed transcripts (1,167 DEmRNAs and 105 DEmiRNAs) were identified in *NORFA*-inhibited porcine GCs. Function annotation by GO and KEGG pathway enrichment analyses showed that *NORFA* might be involved in multiple crucial biological processes and molecular functions, such as metabolism, biosynthesis, response to stress, cell death, TF binding, transcription, enzyme regulation, etc., through affecting important signaling pathways, including TGF-β, PI3K-Akt, Wnt, FoxO, Hippo, cAMP, estrogen, and progesterone synthesis, and ubiquitin-mediated proteolysis signaling pathways. The potential functions of DEmRNAs were also assessed, and they were closely associated with the states and functions of porcine GCs. For example, *ATM*, *ATG5*, *BAD*, *BAK1*, *BAX*, *BCL2L1*, *MCL1*, and *XIAP* are reported to be involved in cell apoptosis (Yousefi et al., 2006; Hellmuth and Stemmann, 2020), which is consistent with the findings in our previous study (Du et al., 2020) and may partially explain the anti-apoptotic effects of *NORFA* on GCs. *CCNJ*, *CCNT1*, and *CCNE2* are associated with cell cycle (Mohamed et al., 2018). Besides, *BRCA1*, *TGF-*β*1*, *TGF-*β*2*, *TGFBR2*, *SMAD2*, *PIK3CA*, *PTEN*, and *WNT5B* are cell proliferation regulators (Miyoshi et al., 2012). *CYP11A1*, *CYP19A1*, *HSD3B1*, *HSD17B1*, and *NR5A1* are associated with estradiol (E2) biosynthesis and secretion (Fuentes and Silveyra, 2019). In addition, *UBA6*, *USP1*, *USP9X*, *USP16*, *USP25*, and *NOVA1* are ubiquitin factors and responsible for proteolysis (Chen et al., 2019a; Raimondi et al., 2019). Moreover, *AR*, *PIK3R1*, *TAB2*, and *TGFBR2* are crucial for cytokine and steroid hormone responses (Hata and Chen, 2016). Based on these DEmRNAs, we hypothesize that *NORFA* also plays an important role in regulating the state (cell cycle, proliferation, and autophagy) and functions (E2 synthesis) of porcine GCs, which has been partially validated in the present study (Figure 8). *In vitro* assays proved that knockdown of *NORFA* significantly induced porcine GC apoptosis, impaired the normal morphology and cell viability, and dramatically inhibited E2 synthesis. Interestingly, we also noticed that NFIX, a transcription factor targeting the promoter of *NORFA* (Du et al., 2020), is dramatically down-regulated in *NORFA*-reduced porcine GCs, indicating that a positive feedback regulatory network between *NORFA* and NFIX exists. Apart from the well-known genes, 202 function-unknown DEmRNAs were identified and further research will focus on investigating their functions.

MicroRNAs are a class of short non-coding RNA (∼21–25 nt), which inhibit the expression of target genes by binding to their 3′-UTR in a base-pairing manner (Treiber et al., 2019). Nowadays, miRNAs are well-known to be involved in a wide range of cellular biological processes, including cell proliferation, differentiation, apoptosis, and even diseases, which are usually under the control of lncRNAs. For instance, *lncRNA-MIR100HG* has been proved to mediate cell apoptosis by influencing the expression of miR-100 and miR-125b (Lu et al., 2017). Zhao et al. demonstrated that *lncRNA-CA7-4* promotes autophagy and apoptosis via regulating miR-877-3p and miR-5680 in vascular endothelial cells (Zhao et al., 2020). In this study, a total of 105 DEmiRNAs (23 up- and 82 down-regulated) were identified in *NORFA*-inhibited porcine GCs. Interestingly, miR-126 is one of the significantly up-regulated miRNAs, which is consistent with our previous finding that *NORFA* negatively regulates the expression of miR-126 by acting as a competing endogenous RNA (ceRNA) (Du et al., 2020). Besides, multiple DEmiRNAs have been reported to play vital roles in ovaries. For example, miR-26b regulates porcine GC apoptosis by directly inhibiting *SMAD4* (Liu et al., 2014). miR-424/SMAD7 and miR-27a/NFAT5 signal axis are actively involved in regulating GC proliferation and cycle progression in cow and mouse (Pande et al., 2018; Tao et al., 2019). Besides, it has been reported that the expression levels of miR-451 and miR-193a-3p in GCs are closely associated with follicular development and diminished ovarian reserve in human and cow (Sontakke et al., 2014; Jiang et al., 2017; Kleaveland et al., 2018). Thus, we provide a hypothesis that *NORFA* may have multiple important functions in porcine GCs via a specific miRNA-dependent manner. It is worth noting that *DROSHA*, a miRNA processing enzyme encoding gene, is significantly up-regulated in *NORFA*-reduced porcine GCs, which may partially lead to the occurrence of multiple DEmiRNA after *NORFA* was knocked down. Moreover, we have identified 83 novel and function-unknown DEmiRNAs, which required deep investigation for their functions. More importantly, the *NORFA*-mediated miRNA–mRNA regulatory network was constructed and partially validated (Figure 6E), which demonstrates that miRNAs is crucial for the regulation of lincRNAs, such as *NORFA*, to the expression of target genes, especially at the post-transcription level.

In recent years, the regulatory mechanism of lncRNA on other RNAs has become a hotspot in biological and medical science, especially for the regulation to miRNAs (Kleaveland et al., 2018). Nowadays, several different mechanisms have been identified, including interaction with chromatin-modifying complexes, epigenetic modifiers, miRNA processing factors, and transcriptional regulatory proteins (Kiontke et al., 2019), and by which lncRNAs could influence the biogenesis and expression of miRNAs. Our findings in this study identified three potential mechanisms for *NORFA* regulation of miRNAs: (i) acting as a ceRNA of miRNAs such as miR-126, which is the same as most lncRNAs; (ii) recruiting specific transcription factors in the promoter region of miRNA; and (iii) controlling miRNA biogenesis (*DROSHA* for instance). Importantly, we have partially validated the mechanism (ii) in this study by identifying the effects of NFIX, a *NORFA*-induced TF, on the expression of its target miRNAs (Figure 7E), which indicates that lincRNAs, such as *NORFA*, may function through TF–miRNA interaction networks. Further studies should focus on whether TFs, like NFIX, regulate the transcription of miRNAs directly or dependent on several transcription co-regulators, and the role of lincRNAs during this process. However, mechanisms (iii) need to be verified by future investigations. It is worth noting that if negative correlations are identified between lncRNAs and miRNAs, two mechanisms may contribute to the regulation process: ceRNA (Thomson and Dinger, 2016) and target-directed miRNA degradation (TDMD) (Ghini et al., 2018). ceRNA method has already been well characterized, while TDMD is a newly identified mechanism for specific miRNA degradation (Sheu-Gruttadauria et al., 2019). In this research, multiple *NORFA*-induced DEmiRNAs were identified and whether the mechanisms mentioned above are involved in this regulatory network need further validation.

It is well known that sow fertility mainly determines the production and profits of pig industry (Cordoba et al., 2015). In the last century, multiple crucial genes including *ESR1*, *FSH*β, *OPN*, *RBP4*, *PRLR*, and *BMP15* have been considered as candidate genes for sow reproductive traits (Rothschild et al., 1996; Zhao et al., 1998). With the development of high-throughput sequencing technology, several novel candidate genes (e.g., *ARID1A*, *IGF2*, and *VEGFA*) and multiple mutations (e.g., ALGA01051183, rs55618224, and *IGFBP2* g.455 A > T) that are associated with sow reproductive traits (e.g., litter size and ovulation rate) were identified by whole-genome sequencing and genome-wide association study (Munoz et al., 2010; Sell-Kubiak et al., 2015; Chen et al., 2019b). Previous studies about screening of candidate genes for sow reproductive traits mainly focused on coding genes, while research on the candidate lncRNA selection for sow reproductive performance has just started and *NORFA* is the first identified candidate lincRNA affecting the high prolificacy of Erhualian pigs. In the current study, several candidate genes for sow reproductive traits are also differentially expressed in *NORFA*-reduced porcine GCs, such as *NCOA1* (Korwin-Kossakowska et al., 2002), *TGF*β*-1* (Wu et al., 2010), *INHBA*, and *TGFBR2* (Li et al., 2020). Their expression patterns were also determined by qRT-PCR (Supplementary Figure 3), and results indicate that *NORFA* may form a regulatory network with these candidate genes and influence sow reproductive traits and fertility. Based on the diverse functions and specific expression patterns, we believe that lincRNAs, such as *NORFA*, are more suitable as candidate genes for the reproductive traits of female mammals.

## Conclusion

In summary, our research provides a worthy strategy to characterize the cellular and transcriptomic alteration in *NORFA*-inhibited porcine GCs for the first time. We have found that *NORFA* is essential for the normal states and function (E2 synthesis) of porcine GCs, and knockdown of *NORFA* leads to dramatic changes in GC transcriptome. More importantly, we have further confirmed that *NORFA* is a candidate gene for sow fertility by controlling several known candidate genes for sow reproductive traits, which provides opportunities to identify potential therapeutic RNA molecules for inhibiting follicular atresia and female infertility.

## Data Availability Statement

The data generated in this study have been deposited into the Sequence Read Archive database (The accession IDs are SRR11793932, SRR11793933, SRR11793934, and SRR11793935).

## Ethics Statement

The animal study was reviewed and approved by Animal Ethics Committee of Nanjing Agricultural University, Nanjing, Jiangsu, China (SYXK (Su) 2015-0656).

## Author Contributions

XD and QFL designed and conceived the research. XD, QL, and LY performed the majority of the experiments. QZ and SW assisted. QL and XD analyzed the data. XD wrote the manuscript. All authors critically reviewed and approved the final version of the manuscript.

## Conflict of Interest

The authors declare that the research was conducted in the absence of any commercial or financial relationships that could be construed as a potential conflict of interest.

## Footnotes

1 http://david.ncifcrf.gov/

2 http://kobas.cbi.pku.edu.cn/anno_iden.php

3 http://www.microrna.gr/miRPathv3/

4 http://string-db.org/

5 http://carolina.imis.athena-innovation.gr

6 http://www.cbs.dtu.dk/services/Promoter/

7 http://jaspar.genereg.net/
