# Supplementary material for: Transcriptomic Data Analyses Reveal That Sow Fertility-Related lincRNA NORFA Is Essential for the Normal States and Functions of Granulosa Cells
Source: Front Cell Dev Biol. 2021 Feb 23;9:610553. doi: 10.3389/fcell.2021.610553 (PMC7940361; doi:10.3389/fcell.2021.610553)
Supplement: Supplementary file 1 [file Data_Sheet_1.docx]

**Supplementary Figures**

**
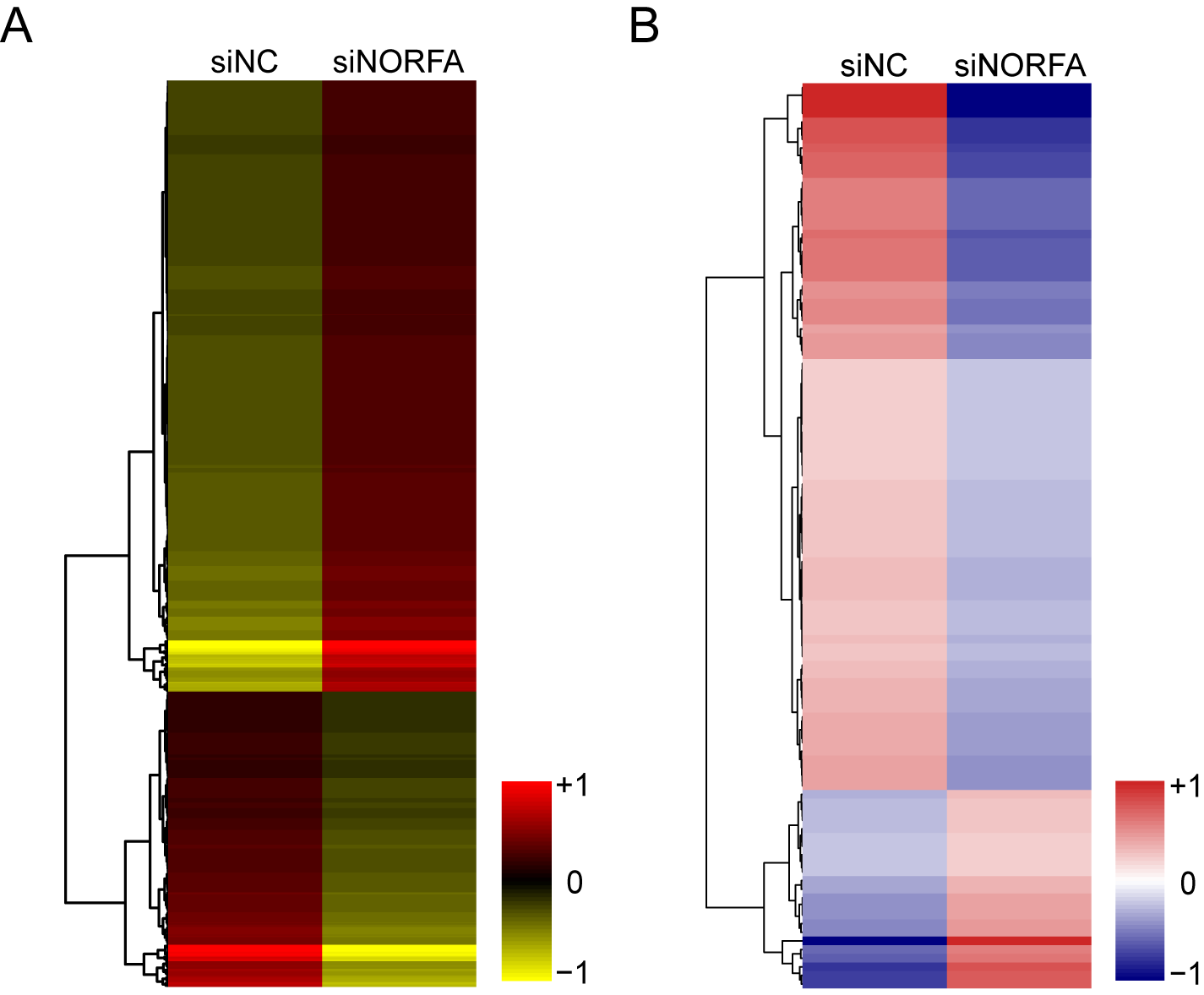
**

**FIGURE S1.** The expression patterns of differentially expressed transcripts in porcine GCs treated with siNORFA. Heatmaps showing the 1167 DEmRNAs (**A**) and 105 DEmiRNAs (**B**) in *NORFA*-reduced porcine GCs. The color scale of heatmaps rang from yellow or blue (low expression) to red (high expression).

**
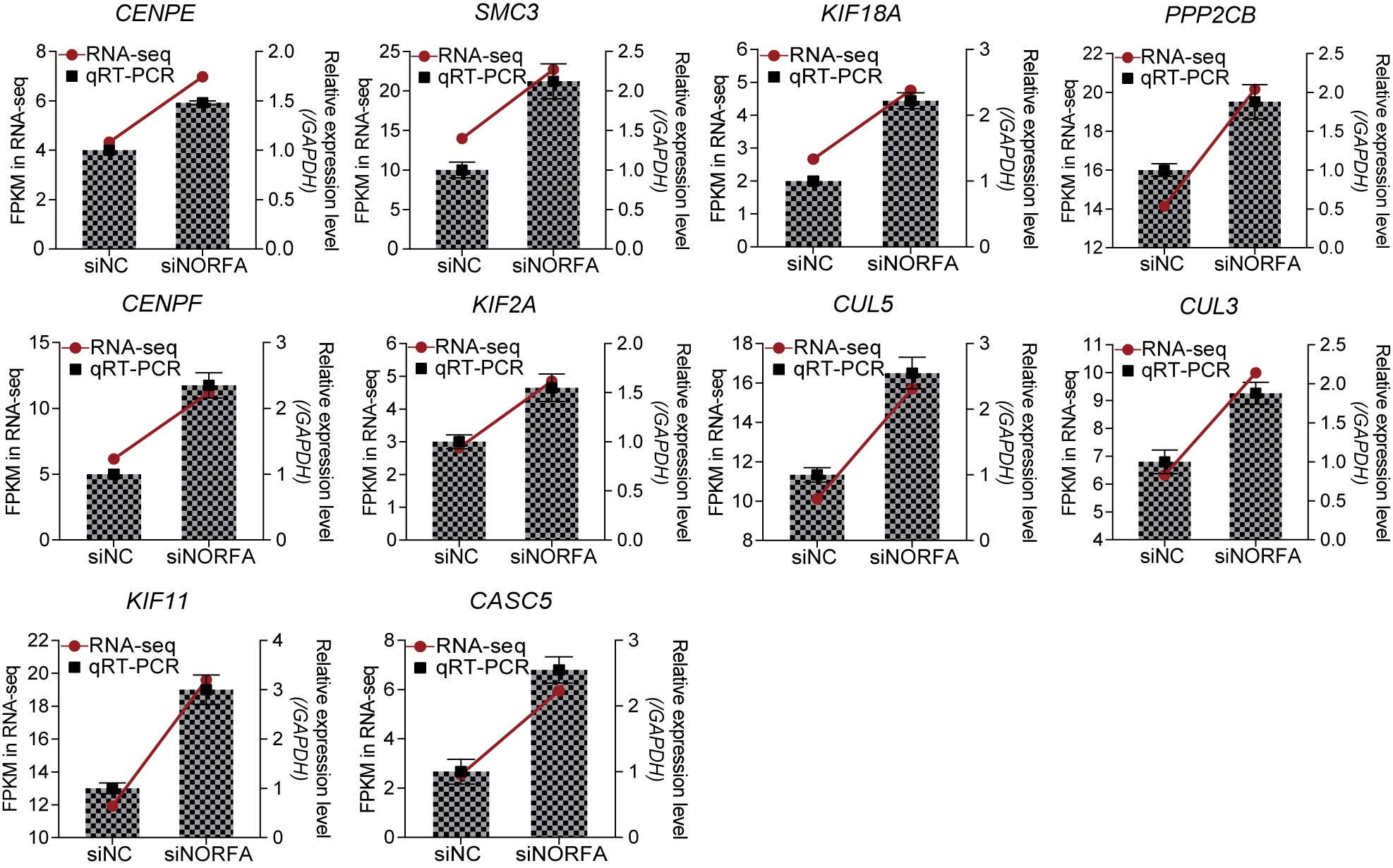
**

**FIGURE S2.** The expression patterns of top 10 hub genes within the protein-protein interaction network were detected by qRT-PCR. The red points indicate the FPKM values of interested genes in RNA-seq data, and bars depict the qRT-PCR results (mean ± S.E.M.; n=3).


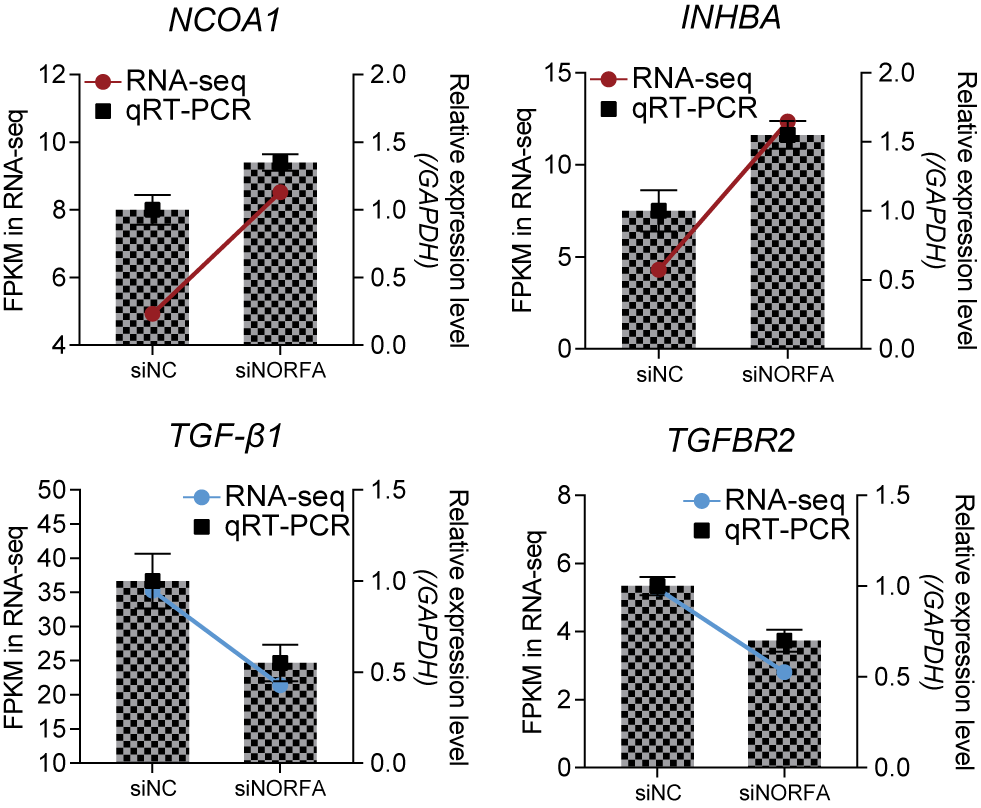


**FIGURE S3.** The expression patterns of four candidate genes for sow fertility in *NORFA*-reduced porcine GCs were detected by qRT-PCR. The points indicate the FPKM values of interested genes in RNA-seq data, and bars depict the qRT-PCR results (mean ± S.E.M.; n=3).

**SUPPLEMENTARY TABLES**

**TABLE S1** Primers used for qRT-PCR in this study.

| **Gene** | **Primers (5’-3’)** | **Tm (℃)** | **Product length (bp)** |
| --- | --- | --- | --- |
| *BAD* | F:TTGAGCAGAGTGAGCAGGAAGAC | 60.0 | 498 |
|  | R:CGGAAGGTCATTGGGAGGG |  |  |
| *BLCAP* | F:ACGGCAGCCTCTGGTCCTTC | 58.5 | 162 |
|  | R:CCTCCTCCTCCTCCTCCTCCTC |  |  |
| *MCL1* | F:TGTGCCAGGCTCACGGACTG | 58.0 | 160 |
|  | R:TCCTCTGCCATGACCACCTCTTC |  |  |
| *INHBA* | F:CTTCTGCCACCTGGACATCATCTG | 60.0 | 149 |
|  | R:GGCTGGCACACTGGCATCTATC |  |  |
| *BAK1* | F:GCTGCTCTGTGCTTACCTGCTC | 60.0 | 137 |
|  | R:ACTCCTGCCAGATCTCCGTCAC |  |  |
| *CCNJ1* | F:AATAGCCCATCCCGTCGTT | 56.0 | 150 |
|  | R:TTGGGCTGCTCTTCTACTTTGG |  |  |
| *IER2* | F:CTGATCCTCGCCTACACCCA | 62.0 | 226 |
|  | R:CGGGCTTTCTTACTCGGGAC |  |  |
| *COL9A2* | F:AGGCGAGAAGGGCGAGTC | 63.0 | 211 |
|  | R:CTGGTCGCTGGCATCTCG |  |  |
| *NOXO1* | F:CTGCGGCTTCTGGAGACCTA | 61.0 | 345 |
|  | R:CGTCTGCTGGTCCTCGTTG |  |  |
| *GPN2* | F:CTTCTCCCAGATGACGCAGTG | 60.0 | 276 |
|  | R:CGATAGTGGCGGAAGAAAGGA |  |  |
| *FGF11* | F:CTCCTCATCCTGCTGTCCAAG | 60.0 | 355 |
|  | R:GAGCAGAGGCGTACAGGACATAG |  |  |
| *HIF-1α* | F:AGGGAGGCAAGAGTGGGAAAT | 58.0 | 219 |
|  | R:GCCGAAATGTACTCCAGTTATCAA |  |  |
| *CENPE* | F:CAGACTTGCTCTGCGACACTC | 59.3 | 337 |
|  | R:AGTCGCAAACCTTCAGCACCT |  |  |
| *SMC3* | F:GTCGAGGAAGCCAGTTTACATC | 58.1 | 146 |
|  | R:CTCTTTATTTGCCTCAGTGTCCT |  |  |
| *KIF18A* | F:GCTGCTGAACCTGGAGTGA | 57.3 | 199 |
|  | R:TCAACCCTTGAACGACTACCC |  |  |
| *PPP2CB* | F:GTCGAGCAGCTGAACGAGTG | 59.1 | 202 |
|  | R:GATAGTTTGTGTCTGGTGATTTTCC |  |  |
| *CENPF* | F:GAAAAAGATTAGAGGCAGAGGT | 57.5 | 114 |
|  | R:CAGATGATGAAGCCTGATGTCG |  |  |
| *KIF2A* | F:TGGTGGGATTACAGGAACGG | 60.9 | 121 |
|  | R:TGGCTCCGAGATGAATGTGC |  |  |
| *CUL5* | F:GGGTAAACAGGGCAGCAATA | 57.5 | 579 |
|  | R:TTCAACATCGGCTCTATCCC |  |  |
| *CUL3* | F:CAATTCAAGAAATCCAGCGTAAG | 55.8 | 358 |
|  | R:TCCCTAATACACCCATAAC |  |  |
| *KIF11* | F:TATCCAGGTGGTGGTGAGAT | 56.3 | 210 |
|  | R:GGACAAACAACACTTCGGTAAA |  |  |
| *CASC5* | F:AATACATTGCTTTGTGCTCCCA | 60.3 | 196 |
|  | R:TGGTGGACTTTTCACTTTTCGTAC |  |  |
| *TGF-β1* | F:GGCACCCCCCACAGCTTATAT | 59.5 | 291 |
|  | R:GTGGGCACTGAGGCGAAAAC |  |  |
| *TGFBR2* | F:TGGCTCCTGAAGTCCTAGAGT | 61.0 | 154 |
|  | R:GAACCAAAGGGTGGCTCAT |  |  |
| *NCOA1* | F:CAGCTACTTGGGTTACAATCAGG | 58.8 | 160 |
|  | R:GGTATGGCTATTTCGTCGTGTT |  |  |
| *INHBA* | F:GGAGATGGTGGAAGCCGT | 59.3 | 100 |
|  | R:GTTCAGAAGCGCCGCCTT |  |  |
| *USP1* | F:GGGACTGAATAATCTCGGCAATA | 60.8 | 308 |
|  | R:GCAGTCGCCTTGGCTGAGTA |  |  |
| *RUNX2* | F:CATTCGCCTCACAAACAACCA | 60.8 | 222 |
|  | R:GCTGAAGAGGCTGTTTGATGC |  |  |
| *PEAK1* | F:GTCACAAATCGGCACCTACTTC | 60.0 | 468 |
|  | R:GCGGTGAAGACTGACCTCTGTTA |  |  |
| *CDKN3* | F:GAAGCCGCCCAGTTCAATAC | 60.3 | 150 |
|  | R:CAACCTGGAAGAGCACATAAACC |  |  |
| *CCDC82* | F:GGTTGATTGGAGGCGGACTA | 59.5 | 124 |
|  | R:ACTGTCAATACTTGCATTATTGTCA |  |  |
| *ATM* | F:CTACAGAACGAAGGAAGGCAGTT | 60.3 | 439 |
|  | R:TCTCGCCACTGTTGCTGAGAT |  |  |
| *TGF-β2* | F:ACGAGGAATACTACGCCAAGGA | 59.9 | 215 |
|  | R:GATCCGTTGTTCAGCCACTCT |  |  |
| *BRCA2* | F:CCGTCAGTTAATACTGTATCTGGGTG | 61.0 | 344 |
|  | R:TCTGCCTGACTGACAGATGGG |  |  |
| *BRCA1* | F:CCTTGGCATTGTGAAACCTCT | 59.8 | 330 |
|  | R:TGACTACTTCCCATAGGTTGTTCG |  |  |
| *PTEN* | F:GGAGATATCAAGAGGATGGATTCG | 60.1 | 359 |
|  | R:TACACCAGTTCGTCCCTTTCC |  |  |
| *LNPK* | F:GGGTCTTCATCCTCCTGGTC | 58.9 | 118 |
|  | R:CAGGGCATACCTGTTCTGTGG |  |  |
| *DROSHA* | F:CATGCCCTGTCCCAATAACC | 60.3 | 274 |
|  | R:CTTTGGGAGCGGGTATGGA |  |  |
| *AR* | F:CCCCAAGTCCAGAGCAGAGG | 60.0 | 347 |
|  | R:CTGTTGCCTTCGGATACTGC |  |  |
| *XIAP* | F:CAGTGCGGTGCTTTAGTTGTCA | 61.2 | 299 |
|  | R:GGCAGGGTTCCTCGGGTAT |  |  |
| *KRAS* | F:CAAGAGTGCCTTGACAATACAGC | 59.5 | 149 |
|  | R:GTACTCCTCTTGACCTGCTGTG |  |  |
| *NFIX* | F:CGAGGAGCGAGCGGTGAA | 60.0 | 213 |
|  | R:TGGCGAAGGCAGTCAATCC |  |  |
| *GAPDH* | F:GGACTCATGACCACGGTCCAT | 60.0 | 220 |
|  | R:TCAGATCCACAACCGACACGT |  |  |
| *U6* | F:TTATGGGTCCTAGCCTGAC | 55.4 | 224 |
|  | R:CACTATTGCGGGTCTGC |  |  |
| miR-126-3p | F:CGGGCTCGTACCGTGAGTAA | 60.0 | 80 |
|  | R:CAGCCACAAAAGAGCACAAT |  |  |
| miR-193a-3p | F:CGGGCAACUGGCCUACAAAG | 60.0 | 76 |
|  | R:CAGCCACAAAAGAGCACAAT |  |  |
| miR-542-3p | F:CGGGCCGCGGCAUAUGGAGGUUC | 60.0 | 79 |
|  | R:CAGCCACAAAAGAGCACAAT |  |  |
| miR-1271 | F:CGGGCUGCCUGCUAUGUG | 60.0 | 74 |
|  | R:CAGCCACAAAAGAGCACAAT |  |  |
| miR-199b-5p | F:CGGGCCCCAGUGUUUAGACU | 60.0 | 76 |
|  | R:CAGCCACAAAAGAGCACAAT |  |  |
| miR-27a-5p | F:CGGGCTTCACAGTGGCTAA | 60.0 | 75 |
|  | R:CAGCCACAAAAGAGCACAAT |  |  |

**TABLE S2** Differentially expressed mRNAs in *NORFA*-reduced porcine GCs.

| **Gene ID** | **FDR** | **log2FC** | **Regulated** | **Gene ID** | **FDR** | **log2FC** | **Regulated** |
| --- | --- | --- | --- | --- | --- | --- | --- |
| Pig_newGene_194813 | 3.16E-33 | -7.066 | down | gene21453 | 2.82E-03 | 0.671 | up |
| gene19996 | 7.94E-31 | -6.955 | down | gene25866 | 5.75E-04 | 0.671 | up |
| gene24136 | 1.58E-29 | -6.815 | down | gene10671 | 2.40E-03 | 0.672 | up |
| gene29811 | 2.51E-27 | -6.241 | down | gene24297 | 3.47E-04 | 0.673 | up |
| Pig_newGene_30420 | 3.16E-26 | -5.488 | down | gene17566 | 4.79E-04 | 0.673 | up |
| gene3831 | 5.01E-14 | -5.320 | down | gene20961 | 3.47E-04 | 0.674 | up |
| Pig_newGene_32920 | 3.16E-12 | -5.298 | down | gene21178 | 3.31E-04 | 0.674 | up |
| gene13673 | 3.98E-13 | -5.246 | down | gene1766 | 3.16E-03 | 0.674 | up |
| Pig_newGene_92712 | 5.01E-35 | -5.220 | down | gene21765 | 3.39E-04 | 0.675 | up |
| gene12983 | 6.31E-23 | -5.198 | down | gene14023 | 3.39E-04 | 0.675 | up |
| Pig_newGene_186083 | 3.98E-26 | -5.152 | down | gene21790 | 1.15E-03 | 0.676 | up |
| Pig_newGene_91913 | 7.41E-12 | -5.122 | down | gene20745 | 3.39E-04 | 0.676 | up |
| gene3709 | 5.89E-13 | -5.006 | down | gene10780 | 3.09E-04 | 0.677 | up |
| gene26899 | 4.57E-14 | -4.905 | down | gene14009 | 3.09E-04 | 0.677 | up |
| Pig_newGene_120224 | 3.55E-15 | -4.276 | down | gene4697 | 3.24E-04 | 0.677 | up |
| gene11849 | 6.31E-12 | -3.865 | down | gene172 | 4.27E-04 | 0.677 | up |
| gene21027 | 5.01E-16 | -3.626 | down | gene1061 | 3.09E-04 | 0.678 | up |
| Pig_newGene_132781 | 3.98E-17 | -3.372 | down | gene11850 | 3.02E-04 | 0.680 | up |
| Pig_newGene_49287 | 6.31E-18 | -3.323 | down | gene24883 | 2.82E-04 | 0.680 | up |
| gene3842 | 5.01E-19 | -3.077 | down | gene906 | 2.57E-04 | 0.681 | up |
| Pig_newGene_120547 | 1.48E-14 | -3.012 | down | gene4760 | 6.46E-03 | 0.681 | up |
| gene11026 | 7.94E-20 | -2.988 | down | gene17816 | 2.95E-04 | 0.682 | up |
| gene14481 | 3.47E-11 | -2.785 | down | gene493 | 3.16E-03 | 0.682 | up |
| gene15881 | 1.26E-21 | -2.631 | down | gene14614 | 4.07E-04 | 0.682 | up |
| gene21800 | 1.74E-10 | -2.629 | down | gene13952 | 6.03E-04 | 0.682 | up |
| gene8166 | 6.61E-15 | -2.627 | down | gene21515 | 7.24E-04 | 0.683 | up |
| Pig_newGene_223945 | 1.12E-16 | -2.550 | down | gene6447 | 2.45E-04 | 0.683 | up |
| gene11177 | 6.17E-22 | -2.478 | down | gene10858 | 2.88E-04 | 0.683 | up |
| gene5164 | 6.31E-23 | -2.407 | down | gene166 | 1.55E-03 | 0.683 | up |
| gene5912 | 5.01E-16 | -2.288 | down | gene6107 | 3.24E-04 | 0.684 | up |
| gene21010 | 6.31E-14 | -2.284 | down | gene11348 | 7.94E-03 | 0.684 | up |
| gene24620 | 5.62E-08 | -2.268 | down | gene26730 | 6.92E-04 | 0.684 | up |
| gene28514 | 7.08E-11 | -2.265 | down | gene13164 | 2.19E-04 | 0.684 | up |
| gene12762 | 3.98E-08 | -2.247 | down | gene5663 | 2.34E-04 | 0.685 | up |
| gene4990 | 2.19E-06 | -2.243 | down | gene1559 | 2.14E-04 | 0.686 | up |
| gene25970 | 2.19E-07 | -2.215 | down | gene734 | 2.09E-04 | 0.686 | up |
| gene5555 | 2.19E-08 | -2.174 | down | gene7114 | 3.72E-03 | 0.687 | up |
| gene9480 | 2.19E-09 | -2.150 | down | gene25516 | 1.70E-03 | 0.687 | up |
| gene12490 | 2.19E-10 | -2.146 | down | gene27740 | 2.63E-02 | 0.688 | up |
| gene17521 | 2.19E-11 | -2.089 | down | gene7319 | 8.91E-04 | 0.689 | up |
| gene8785 | 2.19E-12 | -2.032 | down | gene8003 | 3.02E-04 | 0.689 | up |
| gene8855 | 3.47E-14 | -2.025 | down | gene24959 | 2.95E-02 | 0.689 | up |
| Pig_newGene_49940 | 2.24E-16 | -1.973 | down | gene9327 | 2.69E-04 | 0.689 | up |
| gene25289 | 1.00E-19 | -1.951 | down | gene4624 | 1.95E-04 | 0.690 | up |
| gene5988 | 3.98E-19 | -1.921 | down | gene26645 | 2.24E-04 | 0.690 | up |
| gene5523 | 1.45E-07 | -1.903 | down | gene23167 | 2.04E-04 | 0.690 | up |
| gene30328 | 1.78E-10 | -1.901 | down | gene24322 | 1.15E-02 | 0.691 | up |
| Pig_newGene_14087 | 1.12E-16 | -1.874 | down | gene21227 | 2.19E-04 | 0.692 | up |
| gene13906 | 9.77E-11 | -1.859 | down | gene12364 | 1.82E-04 | 0.693 | up |
| gene5260 | 5.75E-09 | -1.856 | down | gene25057 | 1.62E-04 | 0.693 | up |
| gene17987 | 1.15E-13 | -1.848 | down | gene877 | 1.58E-04 | 0.693 | up |
| gene5909 | 5.01E-19 | -1.843 | down | gene20647 | 8.13E-04 | 0.694 | up |
| gene22760 | 6.31E-20 | -1.818 | down | gene27911 | 1.91E-04 | 0.695 | up |
| gene25321 | 7.76E-10 | -1.756 | down | gene29464 | 1.62E-04 | 0.695 | up |
| gene4292 | 2.00E-12 | -1.751 | down | gene25129 | 2.14E-04 | 0.695 | up |
| gene25566 | 2.51E-12 | -1.738 | down | gene9738 | 1.38E-04 | 0.697 | up |
| gene13741 | 3.16E-12 | -1.722 | down | gene26761 | 2.69E-04 | 0.697 | up |
| gene28006 | 2.82E-05 | -1.719 | down | gene9958 | 3.16E-02 | 0.698 | up |
| gene3566 | 3.98E-12 | -1.705 | down | gene372 | 2.00E-03 | 0.698 | up |
| gene9862 | 5.01E-12 | -1.705 | down | gene15575 | 5.25E-03 | 0.699 | up |
| gene6792 | 4.90E-12 | -1.670 | down | gene24195 | 1.26E-04 | 0.700 | up |
| gene11490 | 6.31E-12 | -1.663 | down | Pig_newGene_138519 | 1.29E-04 | 0.700 | up |
| gene8507 | 2.45E-04 | -1.634 | down | gene916 | 1.58E-04 | 0.700 | up |
| gene5366 | 1.58E-12 | -1.631 | down | gene21462 | 5.75E-03 | 0.701 | up |
| gene11338 | 7.94E-20 | -1.622 | down | gene18533 | 1.15E-04 | 0.701 | up |
| gene21020 | 1.58E-12 | -1.619 | down | gene27603 | 1.58E-04 | 0.701 | up |
| gene5553 | 1.26E-12 | -1.587 | down | gene668 | 1.82E-04 | 0.703 | up |
| gene26902 | 4.90E-04 | -1.573 | down | gene13682 | 4.79E-02 | 0.703 | up |
| gene13915 | 1.82E-04 | -1.551 | down | gene25543 | 1.15E-04 | 0.704 | up |
| gene16869 | 7.59E-13 | -1.549 | down | gene7483 | 1.02E-04 | 0.705 | up |
| gene4054 | 6.31E-13 | -1.546 | down | gene6473 | 1.45E-04 | 0.706 | up |
| gene3323 | 1.26E-20 | -1.545 | down | gene7383 | 3.09E-04 | 0.706 | up |
| gene6539 | 1.58E-19 | -1.519 | down | gene29574 | 1.38E-04 | 0.707 | up |
| gene7857 | 7.08E-08 | -1.519 | down | gene17146 | 4.17E-04 | 0.707 | up |
| gene19486 | 2.00E-18 | -1.495 | down | gene7345 | 1.78E-04 | 0.708 | up |
| gene26117 | 1.02E-06 | -1.491 | down | gene864 | 1.32E-04 | 0.708 | up |
| gene28787 | 2.51E-17 | -1.479 | down | gene20266 | 8.71E-05 | 0.709 | up |
| gene558 | 3.89E-05 | -1.478 | down | gene20764 | 5.13E-04 | 0.709 | up |
| gene16601 | 2.57E-14 | -1.469 | down | gene16483 | 9.12E-05 | 0.710 | up |
| gene24629 | 3.98E-04 | -1.462 | down | gene9366 | 8.32E-05 | 0.710 | up |
| gene30214 | 1.00E-09 | -1.462 | down | gene21167 | 1.05E-04 | 0.710 | up |
| gene5652 | 3.16E-16 | -1.443 | down | gene9814 | 1.62E-03 | 0.712 | up |
| gene11418 | 3.31E-16 | -1.443 | down | gene14979 | 8.32E-05 | 0.712 | up |
| gene4147 | 3.98E-15 | -1.439 | down | gene954 | 2.51E-04 | 0.712 | up |
| gene1933 | 6.92E-06 | -1.435 | down | gene13635 | 3.80E-02 | 0.712 | up |
| Pig_newGene_176539 | 5.01E-14 | -1.422 | down | gene22342 | 2.63E-03 | 0.712 | up |
| gene12073 | 8.71E-04 | -1.421 | down | gene24918 | 1.10E-04 | 0.713 | up |
| gene9687 | 6.31E-13 | -1.416 | down | gene24159 | 8.51E-05 | 0.713 | up |
| gene9930 | 1.78E-15 | -1.407 | down | gene23754 | 7.94E-05 | 0.713 | up |
| gene22289 | 6.61E-11 | -1.402 | down | gene6516 | 7.41E-05 | 0.714 | up |
| gene25280 | 7.94E-12 | -1.398 | down | gene11905 | 7.24E-05 | 0.714 | up |
| gene18874 | 6.03E-09 | -1.397 | down | gene6476 | 7.08E-05 | 0.715 | up |
| gene5820 | 1.45E-15 | -1.394 | down | gene23737 | 1.15E-03 | 0.715 | up |
| gene27619 | 1.00E-10 | -1.394 | down | gene21464 | 6.92E-05 | 0.716 | up |
| gene2779 | 5.37E-06 | -1.384 | down | gene11427 | 7.24E-05 | 0.716 | up |
| gene20590 | 2.24E-16 | -1.371 | down | gene7191 | 1.26E-02 | 0.716 | up |
| gene10408 | 7.76E-04 | -1.344 | down | gene18397 | 6.76E-05 | 0.716 | up |
| Pig_newGene_60888 | 1.02E-10 | -1.338 | down | gene10591 | 6.92E-05 | 0.716 | up |
| gene3841 | 9.55E-10 | -1.334 | down | gene659 | 1.00E-03 | 0.717 | up |
| gene21364 | 5.13E-03 | -1.294 | down | gene5863 | 9.77E-05 | 0.717 | up |
| gene15738 | 1.32E-09 | -1.291 | down | gene26500 | 1.38E-04 | 0.718 | up |
| gene25094 | 2.40E-06 | -1.290 | down | gene7328 | 6.17E-05 | 0.718 | up |
| gene4336 | 1.70E-08 | -1.288 | down | gene4562 | 1.70E-02 | 0.719 | up |
| gene2116 | 1.48E-13 | -1.274 | down | gene333 | 5.75E-05 | 0.719 | up |
| gene10951 | 2.24E-16 | -1.274 | down | gene1401 | 6.46E-05 | 0.720 | up |
| gene3634 | 3.16E-05 | -1.272 | down | gene4609 | 5.62E-05 | 0.721 | up |
| gene11414 | 2.09E-15 | -1.271 | down | gene23806 | 5.62E-05 | 0.721 | up |
| gene10774 | 2.14E-02 | -1.271 | down | gene12372 | 3.09E-03 | 0.722 | up |
| gene6942 | 5.89E-03 | -1.262 | down | gene25465 | 7.94E-05 | 0.722 | up |
| gene10320 | 1.23E-12 | -1.256 | down | gene18060 | 1.91E-04 | 0.723 | up |
| gene7519 | 1.91E-05 | -1.251 | down | gene14834 | 5.25E-05 | 0.723 | up |
| gene2452 | 8.91E-14 | -1.250 | down | gene15487 | 4.90E-05 | 0.724 | up |
| gene25883 | 5.01E-02 | -1.243 | down | gene9334 | 7.41E-05 | 0.724 | up |
| gene19924 | 2.34E-14 | -1.238 | down | gene15508 | 7.76E-05 | 0.725 | up |
| gene2602 | 1.51E-04 | -1.226 | down | gene23574 | 2.04E-04 | 0.725 | up |
| gene25380 | 9.55E-13 | -1.214 | down | gene9250 | 2.40E-03 | 0.726 | up |
| gene2672 | 6.17E-13 | -1.208 | down | gene1959 | 2.04E-04 | 0.727 | up |
| gene27601 | 3.47E-10 | -1.204 | down | gene19913 | 4.07E-05 | 0.727 | up |
| gene981 | 1.82E-03 | -1.198 | down | gene27177 | 2.34E-03 | 0.728 | up |
| gene5996 | 2.57E-15 | -1.194 | down | gene1249 | 8.91E-05 | 0.729 | up |
| gene25314 | 3.24E-06 | -1.193 | down | gene16527 | 8.51E-05 | 0.731 | up |
| gene26438 | 2.63E-03 | -1.192 | down | gene22307 | 3.98E-05 | 0.731 | up |
| gene22768 | 1.10E-04 | -1.184 | down | gene24350 | 4.07E-05 | 0.731 | up |
| gene28090 | 1.38E-10 | -1.180 | down | gene20930 | 8.32E-05 | 0.732 | up |
| gene28851 | 1.15E-08 | -1.177 | down | gene9376 | 2.75E-04 | 0.733 | up |
| gene4229 | 5.25E-08 | -1.173 | down | gene1687 | 3.72E-05 | 0.734 | up |
| gene4934 | 3.09E-15 | -1.171 | down | gene297 | 4.27E-05 | 0.735 | up |
| gene975 | 3.98E-15 | -1.167 | down | gene920 | 2.29E-02 | 0.736 | up |
| gene22171 | 1.02E-13 | -1.164 | down | gene15565 | 4.07E-05 | 0.736 | up |
| gene16880 | 6.92E-15 | -1.163 | down | gene3874 | 1.10E-03 | 0.737 | up |
| gene27284 | 1.10E-13 | -1.163 | down | gene7386 | 1.86E-03 | 0.737 | up |
| gene19247 | 1.62E-08 | -1.160 | down | gene16954 | 1.00E-04 | 0.737 | up |
| Pig_newGene_65620 | 9.33E-06 | -1.159 | down | gene1573 | 7.08E-05 | 0.738 | up |
| gene24682 | 7.24E-14 | -1.151 | down | gene26913 | 2.75E-05 | 0.738 | up |
| gene5761 | 9.12E-14 | -1.148 | down | gene16385 | 4.17E-05 | 0.739 | up |
| gene19824 | 2.19E-09 | -1.146 | down | gene26081 | 8.13E-04 | 0.739 | up |
| gene13411 | 1.35E-08 | -1.146 | down | gene21534 | 1.26E-03 | 0.740 | up |
| gene1073 | 4.47E-02 | -1.141 | down | gene25735 | 4.79E-05 | 0.740 | up |
| gene11492 | 3.39E-14 | -1.138 | down | gene27225 | 2.63E-05 | 0.740 | up |
| gene27236 | 2.88E-12 | -1.135 | down | gene16457 | 1.02E-02 | 0.740 | up |
| gene13924 | 7.08E-05 | -1.131 | down | gene6438 | 2.95E-05 | 0.740 | up |
| gene15815 | 2.57E-13 | -1.131 | down | gene9400 | 2.57E-05 | 0.741 | up |
| gene21994 | 2.40E-11 | -1.130 | down | gene4499 | 2.51E-05 | 0.741 | up |
| gene12099 | 1.38E-06 | -1.127 | down | gene7455 | 4.79E-05 | 0.742 | up |
| gene21415 | 1.05E-04 | -1.119 | down | gene12431 | 2.51E-05 | 0.742 | up |
| gene4287 | 9.55E-04 | -1.111 | down | gene18006 | 2.45E-05 | 0.742 | up |
| gene28355 | 9.77E-13 | -1.110 | down | gene18066 | 2.34E-03 | 0.742 | up |
| gene2398 | 2.51E-03 | -1.102 | down | gene23496 | 1.23E-04 | 0.743 | up |
| gene6128 | 3.55E-10 | -1.086 | down | gene24382 | 6.76E-04 | 0.743 | up |
| Pig_newGene_91530 | 2.24E-10 | -1.084 | down | gene16473 | 2.29E-05 | 0.743 | up |
| gene12091 | 2.09E-11 | -1.082 | down | gene15275 | 2.51E-05 | 0.744 | up |
| gene5146 | 8.71E-11 | -1.072 | down | gene22426 | 2.14E-05 | 0.745 | up |
| gene2314 | 3.39E-12 | -1.066 | down | gene13615 | 4.07E-05 | 0.746 | up |
| gene15354 | 1.70E-08 | -1.059 | down | gene6815 | 2.29E-05 | 0.746 | up |
| gene10202 | 1.20E-03 | -1.058 | down | gene11808 | 2.00E-05 | 0.746 | up |
| gene27810 | 1.32E-07 | -1.053 | down | gene3268 | 2.24E-05 | 0.747 | up |
| gene22723 | 1.45E-04 | -1.051 | down | gene25558 | 2.04E-05 | 0.747 | up |
| gene15064 | 2.34E-06 | -1.049 | down | gene25255 | 3.02E-02 | 0.747 | up |
| gene4968 | 1.32E-07 | -1.048 | down | gene24063 | 4.79E-02 | 0.748 | up |
| gene19249 | 1.95E-02 | -1.042 | down | gene12306 | 9.77E-05 | 0.749 | up |
| gene22726 | 6.17E-05 | -1.039 | down | gene4470 | 1.78E-05 | 0.749 | up |
| gene5550 | 1.86E-02 | -1.039 | down | gene3680 | 5.50E-05 | 0.749 | up |
| gene23687 | 3.55E-10 | -1.036 | down | gene20711 | 2.82E-05 | 0.750 | up |
| gene27869 | 8.91E-09 | -1.031 | down | gene23285 | 1.66E-05 | 0.751 | up |
| gene3132 | 2.95E-08 | -1.025 | down | gene24458 | 1.91E-05 | 0.752 | up |
| gene11310 | 4.47E-05 | -1.022 | down | gene8466 | 4.37E-04 | 0.752 | up |
| gene18735 | 3.55E-10 | -1.014 | down | gene1117 | 1.58E-05 | 0.753 | up |
| Pig_newGene_142742 | 1.82E-02 | -1.013 | down | gene833 | 4.07E-05 | 0.753 | up |
| gene3174 | 5.50E-11 | -1.012 | down | gene15443 | 1.51E-05 | 0.753 | up |
| gene5068 | 7.59E-11 | -1.008 | down | gene13394 | 1.48E-05 | 0.753 | up |
| gene10876 | 3.89E-02 | -1.008 | down | gene12980 | 2.57E-05 | 0.754 | up |
| gene8734 | 2.63E-05 | -1.006 | down | gene1435 | 1.55E-05 | 0.755 | up |
| gene28012 | 1.02E-09 | -0.999 | down | gene21207 | 2.04E-05 | 0.755 | up |
| gene15675 | 1.20E-08 | -0.997 | down | gene27063 | 3.72E-05 | 0.755 | up |
| gene25666 | 1.00E-02 | -0.997 | down | gene19646 | 1.38E-05 | 0.755 | up |
| gene16784 | 3.98E-10 | -0.995 | down | gene9714 | 2.95E-05 | 0.756 | up |
| gene13593 | 4.27E-07 | -0.994 | down | gene1495 | 1.45E-05 | 0.757 | up |
| gene18442 | 1.32E-07 | -0.994 | down | gene23191 | 1.35E-05 | 0.757 | up |
| gene28869 | 1.32E-07 | -0.993 | down | gene17234 | 1.95E-05 | 0.758 | up |
| gene15787 | 3.02E-07 | -0.992 | down | gene12168 | 1.86E-05 | 0.758 | up |
| gene30266 | 3.09E-02 | -0.991 | down | gene14958 | 1.26E-05 | 0.759 | up |
| Pig_newGene_169487 | 2.04E-02 | -0.989 | down | gene23813 | 1.23E-05 | 0.759 | up |
| gene2704 | 2.40E-03 | -0.987 | down | gene15489 | 1.38E-05 | 0.759 | up |
| gene10418 | 2.00E-09 | -0.986 | down | gene930 | 1.45E-05 | 0.759 | up |
| gene8067 | 8.13E-08 | -0.984 | down | gene9391 | 1.15E-05 | 0.759 | up |
| gene18103 | 1.58E-08 | -0.978 | down | gene1624 | 1.15E-05 | 0.761 | up |
| gene22630 | 4.37E-09 | -0.978 | down | gene12148 | 1.10E-05 | 0.761 | up |
| gene2594 | 1.17E-09 | -0.973 | down | gene16487 | 1.10E-05 | 0.762 | up |
| gene4237 | 2.14E-08 | -0.972 | down | gene23269 | 9.55E-06 | 0.762 | up |
| gene20091 | 2.09E-07 | -0.971 | down | gene25157 | 1.38E-05 | 0.763 | up |
| gene8238 | 2.69E-08 | -0.967 | down | gene20680 | 4.79E-04 | 0.765 | up |
| gene11727 | 4.47E-09 | -0.964 | down | gene25113 | 7.08E-05 | 0.765 | up |
| gene6313 | 6.92E-09 | -0.963 | down | gene1283 | 9.33E-06 | 0.766 | up |
| gene28030 | 1.55E-05 | -0.963 | down | gene2081 | 8.51E-03 | 0.766 | up |
| gene2700 | 2.09E-08 | -0.961 | down | gene8857 | 1.07E-05 | 0.766 | up |
| gene24270 | 4.07E-02 | -0.960 | down | gene481 | 1.12E-05 | 0.767 | up |
| gene5512 | 2.45E-06 | -0.957 | down | Pig_newGene_147847 | 1.00E-04 | 0.767 | up |
| gene16244 | 3.89E-07 | -0.956 | down | gene6620 | 4.79E-05 | 0.768 | up |
| gene20889 | 1.26E-08 | -0.953 | down | gene20681 | 8.32E-06 | 0.768 | up |
| gene9006 | 1.02E-06 | -0.952 | down | gene14870 | 1.55E-04 | 0.768 | up |
| gene321 | 2.63E-03 | -0.952 | down | gene15255 | 9.12E-06 | 0.769 | up |
| gene24995 | 4.79E-05 | -0.948 | down | gene25125 | 8.13E-06 | 0.769 | up |
| gene23651 | 3.39E-09 | -0.947 | down | gene20288 | 8.51E-05 | 0.769 | up |
| gene11313 | 8.91E-09 | -0.937 | down | gene9227 | 8.71E-06 | 0.770 | up |
| gene18861 | 7.41E-09 | -0.929 | down | gene21489 | 4.68E-05 | 0.770 | up |
| gene10392 | 1.02E-07 | -0.921 | down | gene24636 | 7.76E-06 | 0.771 | up |
| gene8070 | 1.82E-07 | -0.914 | down | gene10191 | 2.09E-05 | 0.771 | up |
| gene2653 | 4.37E-02 | -0.913 | down | gene22199 | 7.76E-06 | 0.771 | up |
| gene13225 | 5.25E-07 | -0.910 | down | Pig_newGene_60755 | 6.61E-06 | 0.772 | up |
| gene13412 | 1.58E-04 | -0.902 | down | gene11678 | 3.31E-03 | 0.773 | up |
| gene18860 | 2.69E-07 | -0.902 | down | gene4512 | 9.12E-06 | 0.774 | up |
| gene11395 | 1.58E-04 | -0.901 | down | gene10676 | 7.94E-06 | 0.775 | up |
| gene10860 | 5.37E-03 | -0.900 | down | gene21374 | 6.46E-06 | 0.776 | up |
| gene16892 | 5.75E-08 | -0.900 | down | gene4638 | 8.51E-06 | 0.776 | up |
| gene8656 | 3.31E-07 | -0.898 | down | gene11657 | 1.23E-05 | 0.777 | up |
| gene14450 | 2.09E-03 | -0.893 | down | gene7339 | 1.15E-05 | 0.777 | up |
| gene13886 | 3.89E-03 | -0.892 | down | gene23974 | 3.31E-04 | 0.777 | up |
| Pig_newGene_179922 | 3.55E-07 | -0.889 | down | gene23248 | 9.33E-06 | 0.777 | up |
| gene19051 | 9.12E-04 | -0.889 | down | gene7374 | 6.92E-06 | 0.780 | up |
| gene4408 | 1.05E-02 | -0.888 | down | gene12383 | 6.31E-06 | 0.780 | up |
| gene13165 | 3.39E-06 | -0.887 | down | gene1712 | 5.75E-05 | 0.781 | up |
| Pig_newGene_32202 | 2.24E-02 | -0.885 | down | gene24129 | 4.79E-06 | 0.781 | up |
| gene9616 | 4.37E-08 | -0.885 | down | gene10429 | 2.04E-03 | 0.782 | up |
| gene26446 | 1.91E-04 | -0.885 | down | Pig_newGene_194568 | 1.12E-05 | 0.782 | up |
| gene4256 | 2.75E-02 | -0.883 | down | gene1355 | 6.61E-06 | 0.782 | up |
| gene3168 | 8.32E-05 | -0.882 | down | gene891 | 5.37E-06 | 0.783 | up |
| gene23922 | 5.62E-05 | -0.880 | down | gene491 | 3.02E-03 | 0.783 | up |
| gene8960 | 5.50E-08 | -0.877 | down | gene26898 | 2.63E-04 | 0.785 | up |
| gene22683 | 4.90E-04 | -0.876 | down | Pig_newGene_21832 | 5.37E-06 | 0.785 | up |
| gene25672 | 4.68E-05 | -0.873 | down | gene22879 | 4.17E-06 | 0.785 | up |
| Pig_newGene_219658 | 1.70E-02 | -0.871 | down | gene16999 | 2.57E-05 | 0.785 | up |
| gene11231 | 2.51E-07 | -0.869 | down | gene21561 | 3.55E-06 | 0.785 | up |
| gene8636 | 8.91E-08 | -0.868 | down | gene831 | 3.63E-06 | 0.787 | up |
| gene4369 | 3.72E-07 | -0.867 | down | gene13977 | 3.55E-05 | 0.788 | up |
| gene4847 | 5.89E-03 | -0.864 | down | gene20356 | 2.00E-04 | 0.789 | up |
| gene16346 | 2.29E-02 | -0.863 | down | gene27531 | 1.15E-05 | 0.790 | up |
| gene29162 | 5.01E-07 | -0.863 | down | gene27373 | 3.09E-06 | 0.791 | up |
| gene23361 | 2.00E-03 | -0.861 | down | gene873 | 3.09E-06 | 0.791 | up |
| gene10797 | 2.29E-07 | -0.861 | down | gene21233 | 3.16E-06 | 0.791 | up |
| gene773 | 2.57E-05 | -0.848 | down | gene6764 | 3.16E-06 | 0.793 | up |
| gene15230 | 2.95E-02 | -0.848 | down | gene12910 | 1.32E-05 | 0.793 | up |
| gene14748 | 3.39E-07 | -0.848 | down | gene18313 | 1.45E-05 | 0.794 | up |
| Pig_newGene_186067 | 9.77E-07 | -0.845 | down | gene22398 | 5.62E-05 | 0.795 | up |
| gene19398 | 2.69E-06 | -0.844 | down | gene1166 | 7.41E-06 | 0.796 | up |
| gene21518 | 1.12E-06 | -0.838 | down | gene934 | 2.14E-04 | 0.796 | up |
| gene13619 | 8.91E-06 | -0.829 | down | gene7493 | 8.71E-05 | 0.796 | up |
| Pig_newGene_203793 | 2.69E-03 | -0.829 | down | gene24303 | 3.39E-05 | 0.797 | up |
| gene27326 | 9.77E-05 | -0.829 | down | gene8423 | 3.47E-06 | 0.797 | up |
| gene18784 | 1.23E-06 | -0.825 | down | gene23271 | 2.69E-06 | 0.797 | up |
| gene25700 | 2.75E-03 | -0.823 | down | gene11714 | 3.02E-06 | 0.798 | up |
| gene14519 | 9.77E-07 | -0.819 | down | gene21829 | 2.29E-06 | 0.798 | up |
| gene28873 | 1.35E-06 | -0.817 | down | gene20445 | 2.57E-06 | 0.798 | up |
| gene27401 | 1.38E-05 | -0.815 | down | gene17660 | 3.31E-06 | 0.799 | up |
| gene20564 | 9.33E-06 | -0.813 | down | gene8139 | 2.24E-06 | 0.799 | up |
| gene6199 | 1.07E-06 | -0.813 | down | gene9310 | 3.98E-06 | 0.799 | up |
| gene11104 | 1.51E-03 | -0.812 | down | gene9933 | 2.24E-06 | 0.799 | up |
| gene26760 | 2.19E-06 | -0.810 | down | gene10027 | 2.45E-06 | 0.799 | up |
| gene19918 | 1.41E-04 | -0.807 | down | gene27041 | 2.63E-05 | 0.799 | up |
| gene25030 | 2.57E-02 | -0.794 | down | gene26664 | 2.51E-06 | 0.800 | up |
| gene21838 | 3.89E-05 | -0.792 | down | gene23836 | 2.09E-06 | 0.800 | up |
| gene23700 | 1.35E-05 | -0.790 | down | gene9306 | 4.07E-03 | 0.802 | up |
| gene11039 | 5.89E-04 | -0.790 | down | gene24278 | 2.75E-04 | 0.803 | up |
| gene25918 | 7.76E-05 | -0.789 | down | gene10657 | 1.74E-02 | 0.803 | up |
| gene1905 | 1.62E-03 | -0.787 | down | gene461 | 1.66E-06 | 0.804 | up |
| gene8778 | 4.07E-02 | -0.781 | down | gene15637 | 3.16E-06 | 0.805 | up |
| gene18432 | 3.02E-04 | -0.776 | down | gene27783 | 1.02E-05 | 0.806 | up |
| gene25941 | 1.45E-05 | -0.775 | down | gene17418 | 2.82E-06 | 0.806 | up |
| gene23886 | 5.89E-04 | -0.771 | down | gene25116 | 1.23E-03 | 0.806 | up |
| gene4264 | 7.24E-03 | -0.769 | down | gene4800 | 1.20E-05 | 0.808 | up |
| gene12642 | 8.32E-06 | -0.768 | down | gene1701 | 2.57E-06 | 0.809 | up |
| gene18160 | 1.23E-04 | -0.765 | down | gene16982 | 3.47E-06 | 0.809 | up |
| gene14929 | 3.24E-05 | -0.764 | down | gene9428 | 1.35E-06 | 0.810 | up |
| Pig_newGene_128933 | 5.25E-05 | -0.760 | down | gene18323 | 1.41E-06 | 0.810 | up |
| gene2462 | 1.48E-05 | -0.757 | down | gene17703 | 4.79E-06 | 0.811 | up |
| gene23675 | 2.75E-05 | -0.754 | down | gene20868 | 3.63E-06 | 0.812 | up |
| gene22472 | 2.14E-05 | -0.750 | down | gene27659 | 1.58E-06 | 0.813 | up |
| gene19575 | 3.16E-02 | -0.749 | down | gene17186 | 2.09E-05 | 0.814 | up |
| gene56 | 5.37E-05 | -0.746 | down | gene25024 | 2.14E-06 | 0.814 | up |
| Pig_newGene_69060 | 2.19E-05 | -0.745 | down | gene21123 | 1.66E-06 | 0.815 | up |
| gene5310 | 2.19E-05 | -0.743 | down | gene14406 | 2.82E-05 | 0.817 | up |
| gene19763 | 6.03E-05 | -0.742 | down | gene25584 | 7.94E-07 | 0.821 | up |
| gene3995 | 2.04E-03 | -0.742 | down | gene13951 | 9.33E-07 | 0.822 | up |
| gene17482 | 3.98E-05 | -0.741 | down | gene3269 | 7.08E-07 | 0.822 | up |
| gene7982 | 1.26E-03 | -0.728 | down | gene25501 | 3.89E-06 | 0.827 | up |
| gene23371 | 3.02E-02 | -0.728 | down | gene9385 | 3.55E-06 | 0.828 | up |
| gene2634 | 1.07E-02 | -0.725 | down | gene1393 | 5.37E-07 | 0.830 | up |
| gene20202 | 5.62E-05 | -0.724 | down | gene18507 | 8.13E-06 | 0.832 | up |
| gene27802 | 5.01E-03 | -0.720 | down | gene4658 | 6.92E-07 | 0.832 | up |
| gene17874 | 2.88E-02 | -0.719 | down | gene9972 | 7.59E-06 | 0.833 | up |
| gene28004 | 1.45E-04 | -0.717 | down | gene23530 | 4.90E-07 | 0.834 | up |
| Pig_newGene_121789 | 2.19E-04 | -0.711 | down | gene2115 | 1.58E-04 | 0.835 | up |
| gene26432 | 1.74E-04 | -0.711 | down | gene21212 | 8.91E-07 | 0.835 | up |
| gene9592 | 2.82E-04 | -0.710 | down | gene27750 | 4.27E-07 | 0.836 | up |
| Pig_newGene_110928 | 1.02E-02 | -0.708 | down | gene4575 | 1.38E-06 | 0.836 | up |
| gene1135 | 2.19E-04 | -0.707 | down | gene21670 | 7.41E-07 | 0.836 | up |
| gene4351 | 2.51E-03 | -0.707 | down | gene473 | 4.37E-07 | 0.838 | up |
| gene6054 | 1.15E-04 | -0.705 | down | gene1418 | 5.13E-07 | 0.839 | up |
| gene28886 | 1.17E-04 | -0.701 | down | gene24291 | 2.34E-06 | 0.839 | up |
| gene22147 | 1.41E-03 | -0.700 | down | gene19336 | 7.41E-06 | 0.839 | up |
| gene19770 | 3.09E-02 | -0.699 | down | gene29285 | 1.41E-03 | 0.840 | up |
| gene17984 | 2.88E-04 | -0.697 | down | gene23183 | 3.02E-07 | 0.844 | up |
| gene19163 | 5.89E-03 | -0.697 | down | gene13544 | 3.02E-07 | 0.844 | up |
| gene5539 | 4.57E-03 | -0.694 | down | gene18233 | 3.16E-07 | 0.845 | up |
| gene20421 | 4.27E-04 | -0.691 | down | gene23224 | 7.94E-07 | 0.845 | up |
| gene17865 | 2.57E-03 | -0.690 | down | gene7443 | 3.24E-07 | 0.846 | up |
| gene6219 | 1.10E-03 | -0.689 | down | gene22955 | 5.37E-07 | 0.848 | up |
| gene23006 | 3.39E-02 | -0.688 | down | gene9347 | 2.45E-07 | 0.848 | up |
| gene20568 | 3.63E-02 | -0.688 | down | gene4888 | 5.01E-04 | 0.849 | up |
| gene22498 | 8.51E-04 | -0.687 | down | gene27572 | 2.19E-07 | 0.849 | up |
| gene3836 | 3.24E-04 | -0.687 | down | gene26715 | 2.09E-07 | 0.850 | up |
| gene20003 | 2.69E-04 | -0.686 | down | gene678 | 1.95E-04 | 0.851 | up |
| gene28039 | 2.69E-04 | -0.683 | down | gene554 | 6.76E-07 | 0.851 | up |
| gene29161 | 5.62E-04 | -0.681 | down | gene8311 | 2.75E-07 | 0.853 | up |
| gene24477 | 2.88E-04 | -0.681 | down | gene21743 | 1.05E-06 | 0.854 | up |
| gene23670 | 9.77E-03 | -0.678 | down | gene17951 | 2.69E-06 | 0.854 | up |
| gene10198 | 5.13E-04 | -0.675 | down | gene10241 | 7.24E-07 | 0.855 | up |
| gene5421 | 3.39E-02 | -0.672 | down | gene7295 | 2.95E-04 | 0.855 | up |
| gene27309 | 4.79E-04 | -0.670 | down | gene27344 | 1.66E-07 | 0.855 | up |
| gene11069 | 3.55E-04 | -0.670 | down | gene21142 | 9.55E-03 | 0.855 | up |
| gene23341 | 8.51E-04 | -0.669 | down | gene21628 | 2.29E-07 | 0.857 | up |
| gene10440 | 4.57E-04 | -0.669 | down | gene596 | 2.34E-07 | 0.857 | up |
| gene5342 | 3.39E-02 | -0.669 | down | gene148 | 1.95E-07 | 0.857 | up |
| gene13298 | 5.13E-04 | -0.663 | down | gene4799 | 1.35E-05 | 0.859 | up |
| gene25894 | 3.39E-02 | -0.662 | down | gene486 | 6.92E-07 | 0.859 | up |
| gene6048 | 3.98E-03 | -0.662 | down | gene10677 | 2.40E-07 | 0.861 | up |
| gene6243 | 1.48E-02 | -0.661 | down | gene899 | 1.00E-07 | 0.862 | up |
| gene19485 | 4.27E-02 | -0.656 | down | gene12070 | 1.66E-04 | 0.862 | up |
| gene19912 | 8.51E-04 | -0.655 | down | gene25914 | 7.08E-07 | 0.868 | up |
| Pig_newGene_29195 | 7.59E-03 | -0.655 | down | gene5871 | 1.74E-07 | 0.868 | up |
| gene3992 | 2.75E-03 | -0.650 | down | gene23321 | 4.90E-07 | 0.868 | up |
| gene20179 | 9.12E-04 | -0.649 | down | gene17793 | 5.01E-07 | 0.869 | up |
| gene8584 | 8.51E-04 | -0.648 | down | gene18310 | 2.24E-06 | 0.870 | up |
| gene25882 | 1.10E-03 | -0.648 | down | gene17651 | 1.26E-07 | 0.871 | up |
| Pig_newGene_36814 | 1.15E-03 | -0.645 | down | gene4673 | 5.01E-07 | 0.872 | up |
| gene20015 | 5.13E-03 | -0.645 | down | gene18512 | 6.76E-07 | 0.873 | up |
| gene14528 | 9.33E-04 | -0.644 | down | gene1467 | 8.71E-08 | 0.876 | up |
| gene8581 | 1.05E-03 | -0.644 | down | gene25120 | 5.50E-04 | 0.879 | up |
| gene4218 | 1.62E-03 | -0.643 | down | gene20257 | 5.01E-07 | 0.880 | up |
| gene13401 | 7.41E-03 | -0.643 | down | gene19182 | 7.94E-06 | 0.881 | up |
| gene19346 | 1.70E-03 | -0.639 | down | gene1508 | 2.45E-06 | 0.882 | up |
| gene20655 | 1.78E-03 | -0.639 | down | gene17180 | 8.13E-07 | 0.886 | up |
| gene24563 | 1.78E-03 | -0.636 | down | gene16419 | 5.37E-08 | 0.886 | up |
| gene11077 | 3.47E-02 | -0.635 | down | gene10662 | 5.50E-08 | 0.887 | up |
| gene3702 | 5.62E-03 | -0.634 | down | gene6189 | 3.98E-08 | 0.887 | up |
| Pig_newGene_14085 | 2.19E-03 | -0.633 | down | gene24691 | 1.82E-03 | 0.887 | up |
| gene10793 | 1.38E-03 | -0.633 | down | gene17111 | 3.55E-08 | 0.888 | up |
| gene13258 | 1.41E-03 | -0.632 | down | gene23518 | 4.47E-02 | 0.889 | up |
| gene4654 | 1.95E-03 | -0.632 | down | gene17417 | 5.13E-05 | 0.889 | up |
| gene11088 | 1.91E-03 | -0.631 | down | Pig_newGene_112338 | 3.47E-08 | 0.890 | up |
| gene3148 | 4.68E-02 | -0.631 | down | gene15475 | 2.34E-06 | 0.891 | up |
| gene2102 | 7.24E-03 | -0.628 | down | gene15485 | 8.71E-08 | 0.891 | up |
| gene11685 | 2.95E-02 | -0.628 | down | gene18403 | 4.57E-08 | 0.892 | up |
| gene13775 | 9.55E-03 | -0.628 | down | gene26256 | 7.08E-03 | 0.894 | up |
| Pig_newGene_209027 | 3.47E-03 | -0.623 | down | gene599 | 2.19E-04 | 0.895 | up |
| Pig_newGene_137316 | 7.76E-03 | -0.620 | down | gene15011 | 1.15E-07 | 0.895 | up |
| gene7995 | 2.14E-03 | -0.620 | down | gene7549 | 7.08E-08 | 0.895 | up |
| gene28117 | 3.47E-03 | -0.620 | down | gene24258 | 2.40E-08 | 0.896 | up |
| gene3570 | 3.98E-02 | -0.619 | down | gene1769 | 5.37E-08 | 0.896 | up |
| gene11079 | 2.69E-03 | -0.619 | down | gene24942 | 3.24E-08 | 0.898 | up |
| gene14509 | 2.51E-03 | -0.616 | down | gene3250 | 6.31E-08 | 0.899 | up |
| gene10214 | 7.24E-03 | -0.616 | down | gene441 | 1.12E-07 | 0.903 | up |
| gene25770 | 4.17E-02 | -0.616 | down | gene14613 | 1.70E-08 | 0.903 | up |
| gene20185 | 2.63E-03 | -0.615 | down | gene27062 | 5.13E-08 | 0.904 | up |
| gene10303 | 7.41E-03 | -0.614 | down | gene21217 | 1.58E-08 | 0.904 | up |
| gene2221 | 4.79E-02 | -0.613 | down | gene9998 | 1.78E-08 | 0.905 | up |
| gene18831 | 2.95E-02 | -0.613 | down | gene17424 | 5.62E-08 | 0.905 | up |
| Pig_newGene_158432 | 3.16E-03 | -0.609 | down | gene25163 | 2.51E-08 | 0.906 | up |
| gene17840 | 3.24E-03 | -0.607 | down | gene16320 | 4.37E-07 | 0.907 | up |
| gene6935 | 3.72E-03 | -0.607 | down | gene20000 | 2.04E-06 | 0.909 | up |
| gene22647 | 9.77E-03 | -0.606 | down | gene28824 | 1.35E-05 | 0.910 | up |
| gene10059 | 1.78E-02 | -0.602 | down | Pig_newGene_205492 | 1.74E-04 | 0.911 | up |
| gene22652 | 3.89E-02 | -0.600 | down | gene11421 | 3.72E-02 | 0.912 | up |
| gene26214 | 7.24E-03 | -0.600 | down | gene9333 | 1.12E-07 | 0.913 | up |
| gene13390 | 4.90E-03 | -0.598 | down | gene28228 | 2.09E-07 | 0.913 | up |
| gene13365 | 3.02E-02 | -0.598 | down | gene18338 | 8.51E-09 | 0.914 | up |
| gene14725 | 1.26E-02 | -0.596 | down | gene19691 | 1.02E-08 | 0.916 | up |
| gene16518 | 2.09E-02 | -0.594 | down | gene1480 | 1.35E-08 | 0.918 | up |
| gene13173 | 5.37E-03 | -0.593 | down | gene21120 | 5.50E-08 | 0.919 | up |
| gene20817 | 6.46E-03 | -0.592 | down | gene15198 | 1.66E-08 | 0.922 | up |
| gene19980 | 7.24E-03 | -0.591 | down | gene22810 | 4.68E-09 | 0.924 | up |
| gene9483 | 8.32E-03 | -0.588 | up | gene16486 | 2.51E-06 | 0.927 | up |
| gene5125 | 7.41E-03 | -0.585 | up | gene26822 | 3.80E-09 | 0.931 | up |
| gene15511 | 7.24E-03 | 0.585 | up | gene9346 | 3.98E-09 | 0.934 | up |
| gene7300 | 7.76E-03 | 0.585 | up | Pig_newGene_64174 | 5.62E-09 | 0.934 | up |
| gene26603 | 6.92E-03 | 0.586 | up | gene6844 | 4.90E-08 | 0.935 | up |
| gene22817 | 2.51E-02 | 0.586 | up | gene24446 | 2.57E-08 | 0.935 | up |
| gene23972 | 7.24E-03 | 0.586 | up | gene27394 | 1.12E-08 | 0.935 | up |
| gene27245 | 4.37E-02 | 0.586 | up | gene9843 | 2.00E-05 | 0.938 | up |
| gene24884 | 6.61E-03 | 0.586 | up | gene24406 | 3.09E-09 | 0.940 | up |
| gene27753 | 7.41E-03 | 0.587 | up | gene21299 | 8.51E-09 | 0.941 | up |
| gene13432 | 4.47E-02 | 0.588 | up | gene15677 | 4.68E-06 | 0.942 | up |
| gene15450 | 6.46E-03 | 0.588 | up | gene6390 | 9.33E-09 | 0.945 | up |
| gene26655 | 6.46E-03 | 0.588 | up | gene18045 | 5.25E-04 | 0.945 | up |
| Pig_newGene_16135 | 6.76E-03 | 0.588 | up | gene13699 | 1.35E-09 | 0.949 | up |
| gene24280 | 6.17E-03 | 0.588 | up | Pig_newGene_183134 | 3.16E-07 | 0.952 | up |
| gene21395 | 9.55E-03 | 0.589 | up | gene1968 | 1.32E-09 | 0.954 | up |
| gene9746 | 7.08E-03 | 0.590 | up | gene19777 | 3.09E-09 | 0.955 | up |
| gene10653 | 6.46E-03 | 0.590 | up | gene21509 | 1.62E-06 | 0.959 | up |
| gene21151 | 7.59E-03 | 0.590 | up | gene27579 | 7.94E-10 | 0.962 | up |
| gene15312 | 6.92E-03 | 0.590 | up | gene15132 | 4.27E-09 | 0.964 | up |
| gene15256 | 1.91E-02 | 0.591 | up | gene17283 | 2.09E-03 | 0.965 | up |
| gene341 | 5.89E-03 | 0.591 | up | gene7183 | 1.29E-09 | 0.968 | up |
| gene19513 | 6.17E-03 | 0.591 | up | gene1071 | 8.71E-10 | 0.969 | up |
| gene25557 | 6.46E-03 | 0.591 | up | gene4215 | 9.12E-03 | 0.970 | up |
| gene25134 | 2.09E-02 | 0.592 | up | gene1691 | 1.70E-04 | 0.970 | up |
| gene15407 | 5.50E-03 | 0.592 | up | gene21836 | 5.75E-03 | 0.973 | up |
| gene17636 | 5.50E-03 | 0.593 | up | gene20497 | 8.91E-10 | 0.974 | up |
| gene24044 | 9.33E-03 | 0.593 | up | gene12388 | 5.25E-09 | 0.975 | up |
| gene9246 | 2.63E-02 | 0.594 | up | gene14853 | 6.76E-10 | 0.977 | up |
| gene26777 | 5.13E-03 | 0.595 | up | gene869 | 4.57E-08 | 0.977 | up |
| gene27664 | 7.24E-03 | 0.595 | up | gene10801 | 3.72E-09 | 0.983 | up |
| gene13964 | 5.01E-03 | 0.596 | up | gene12611 | 4.27E-09 | 0.990 | up |
| gene25112 | 9.12E-03 | 0.597 | up | gene13374 | 1.32E-09 | 0.993 | up |
| gene14967 | 5.01E-03 | 0.598 | up | gene18691 | 1.15E-09 | 0.995 | up |
| gene1815 | 4.90E-03 | 0.598 | up | gene11986 | 1.38E-10 | 0.996 | up |
| gene5235 | 5.75E-03 | 0.598 | up | gene19651 | 1.41E-10 | 0.997 | up |
| gene13866 | 4.79E-03 | 0.599 | up | gene4545 | 1.62E-10 | 0.998 | up |
| gene27775 | 6.76E-03 | 0.599 | up | gene23464 | 1.62E-08 | 1.000 | up |
| gene24935 | 4.79E-03 | 0.600 | up | gene9377 | 4.17E-10 | 1.002 | up |
| gene2993 | 4.90E-03 | 0.600 | up | gene8448 | 1.45E-10 | 1.008 | up |
| gene4812 | 5.75E-03 | 0.600 | up | gene13140 | 1.66E-08 | 1.008 | up |
| gene396 | 4.37E-03 | 0.601 | up | gene8157 | 7.94E-07 | 1.014 | up |
| gene1486 | 8.71E-03 | 0.601 | up | gene4577 | 1.15E-03 | 1.021 | up |
| gene25658 | 5.01E-03 | 0.601 | up | gene17018 | 9.77E-09 | 1.026 | up |
| gene7247 | 4.27E-03 | 0.601 | up | gene21503 | 3.89E-11 | 1.032 | up |
| gene1651 | 4.07E-03 | 0.602 | up | gene496 | 1.55E-09 | 1.038 | up |
| gene8202 | 3.72E-03 | 0.603 | up | gene8248 | 1.91E-11 | 1.038 | up |
| gene21799 | 3.80E-03 | 0.605 | up | gene9307 | 1.66E-09 | 1.038 | up |
| gene5032 | 3.89E-03 | 0.605 | up | gene772 | 7.94E-09 | 1.039 | up |
| gene10501 | 3.63E-03 | 0.606 | up | Pig_newGene_205491 | 1.62E-11 | 1.045 | up |
| gene11997 | 4.47E-03 | 0.606 | up | gene28005 | 2.69E-10 | 1.045 | up |
| gene7575 | 2.57E-02 | 0.607 | up | gene538 | 3.02E-11 | 1.048 | up |
| gene9139 | 3.39E-03 | 0.607 | up | gene22805 | 7.94E-04 | 1.051 | up |
| gene3286 | 5.62E-03 | 0.608 | up | gene15007 | 1.20E-11 | 1.056 | up |
| gene4835 | 3.80E-03 | 0.608 | up | gene20550 | 3.63E-06 | 1.056 | up |
| gene23527 | 1.12E-02 | 0.608 | up | gene22802 | 5.25E-12 | 1.058 | up |
| gene12159 | 3.39E-03 | 0.609 | up | gene19574 | 7.59E-05 | 1.059 | up |
| gene192 | 3.31E-03 | 0.610 | up | gene5989 | 4.47E-11 | 1.060 | up |
| gene18552 | 1.07E-02 | 0.610 | up | gene1918 | 5.37E-07 | 1.062 | up |
| gene21848 | 3.47E-03 | 0.610 | up | gene2619 | 1.51E-10 | 1.063 | up |
| Pig_newGene_211530 | 3.09E-03 | 0.610 | up | gene17989 | 1.91E-12 | 1.066 | up |
| gene19131 | 3.47E-03 | 0.611 | up | gene30088 | 7.41E-12 | 1.073 | up |
| gene24926 | 1.10E-02 | 0.611 | up | gene248 | 7.41E-11 | 1.073 | up |
| gene7594 | 3.24E-03 | 0.611 | up | gene14010 | 1.51E-12 | 1.075 | up |
| gene18668 | 3.63E-03 | 0.611 | up | gene5574 | 2.19E-04 | 1.079 | up |
| gene15110 | 3.39E-03 | 0.611 | up | gene13994 | 8.32E-13 | 1.080 | up |
| gene931 | 3.89E-03 | 0.612 | up | gene7288 | 1.78E-12 | 1.082 | up |
| gene1322 | 2.82E-03 | 0.612 | up | gene15706 | 3.80E-07 | 1.088 | up |
| Pig_newGene_203853 | 3.98E-03 | 0.613 | up | gene1352 | 1.95E-12 | 1.089 | up |
| gene202 | 3.16E-03 | 0.614 | up | gene22840 | 1.95E-13 | 1.102 | up |
| gene29160 | 3.24E-03 | 0.614 | up | gene20437 | 3.31E-08 | 1.114 | up |
| gene8475 | 3.24E-03 | 0.614 | up | gene13053 | 6.03E-12 | 1.117 | up |
| gene18105 | 2.57E-03 | 0.615 | up | gene16961 | 6.17E-12 | 1.117 | up |
| gene23216 | 3.16E-03 | 0.615 | up | gene26565 | 1.78E-13 | 1.118 | up |
| gene4781 | 2.75E-03 | 0.616 | up | gene15610 | 3.63E-11 | 1.118 | up |
| gene7430 | 2.45E-03 | 0.617 | up | gene12258 | 8.91E-14 | 1.125 | up |
| gene4881 | 2.45E-03 | 0.617 | up | gene8136 | 3.89E-14 | 1.126 | up |
| gene23554 | 8.71E-03 | 0.618 | up | gene27781 | 6.76E-06 | 1.138 | up |
| gene8096 | 2.45E-03 | 0.618 | up | gene8724 | 1.48E-14 | 1.140 | up |
| gene13265 | 5.01E-03 | 0.619 | up | gene17685 | 9.55E-12 | 1.142 | up |
| gene1563 | 2.95E-02 | 0.619 | up | gene8053 | 1.62E-07 | 1.146 | up |
| gene27192 | 2.45E-03 | 0.619 | up | gene21266 | 8.32E-08 | 1.150 | up |
| gene19711 | 2.40E-03 | 0.620 | up | gene23973 | 1.38E-12 | 1.151 | up |
| gene17307 | 2.29E-03 | 0.620 | up | gene2731 | 9.33E-09 | 1.152 | up |
| gene17043 | 2.45E-03 | 0.621 | up | gene23804 | 2.63E-13 | 1.158 | up |
| gene13749 | 2.51E-03 | 0.621 | up | gene18505 | 2.00E-14 | 1.168 | up |
| gene15736 | 2.45E-03 | 0.621 | up | gene27776 | 5.89E-15 | 1.175 | up |
| gene21425 | 4.47E-03 | 0.621 | up | gene3173 | 5.50E-03 | 1.175 | up |
| gene25102 | 2.19E-03 | 0.621 | up | gene9986 | 6.03E-15 | 1.192 | up |
| gene7153 | 2.19E-03 | 0.621 | up | Pig_newGene_207848 | 8.91E-10 | 1.193 | up |
| gene15634 | 2.19E-03 | 0.621 | up | gene10664 | 1.78E-10 | 1.196 | up |
| gene27834 | 1.12E-02 | 0.622 | up | gene27818 | 4.79E-09 | 1.197 | up |
| gene22215 | 3.98E-03 | 0.622 | up | gene24114 | 5.89E-14 | 1.198 | up |
| gene6432 | 2.45E-03 | 0.623 | up | gene1394 | 1.95E-13 | 1.198 | up |
| gene9763 | 2.00E-03 | 0.623 | up | gene22919 | 8.91E-16 | 1.199 | up |
| gene532 | 3.89E-03 | 0.623 | up | gene19492 | 2.09E-14 | 1.216 | up |
| gene1359 | 1.95E-03 | 0.624 | up | gene4672 | 1.35E-14 | 1.217 | up |
| gene2278 | 2.63E-03 | 0.624 | up | gene18481 | 9.55E-15 | 1.217 | up |
| gene18312 | 2.19E-03 | 0.624 | up | gene8535 | 1.58E-04 | 1.238 | up |
| gene2019 | 2.29E-03 | 0.625 | up | Pig_newGene_223647 | 6.61E-16 | 1.240 | up |
| gene3169 | 2.51E-03 | 0.625 | up | gene12339 | 1.78E-14 | 1.254 | up |
| gene299 | 1.91E-03 | 0.626 | up | gene25580 | 2.34E-03 | 1.269 | up |
| gene15400 | 2.04E-03 | 0.626 | up | Pig_newGene_221933 | 9.12E-11 | 1.274 | up |
| gene24009 | 4.27E-02 | 0.626 | up | gene19505 | 1.00E-08 | 1.278 | up |
| gene9153 | 2.34E-03 | 0.626 | up | gene3652 | 1.58E-13 | 1.283 | up |
| gene10678 | 1.45E-02 | 0.626 | up | gene6545 | 5.01E-14 | 1.284 | up |
| gene518 | 2.45E-02 | 0.626 | up | gene26869 | 5.01E-11 | 1.290 | up |
| gene15015 | 2.04E-02 | 0.626 | up | Pig_newGene_105251 | 4.57E-05 | 1.292 | up |
| gene6277 | 1.91E-03 | 0.626 | up | Pig_newGene_155596 | 6.03E-05 | 1.293 | up |
| gene27111 | 1.78E-03 | 0.627 | up | gene24043 | 3.98E-13 | 1.295 | up |
| gene24204 | 2.00E-03 | 0.627 | up | gene19457 | 6.61E-16 | 1.301 | up |
| gene22380 | 1.91E-03 | 0.627 | up | Pig_newGene_35665 | 1.17E-04 | 1.304 | up |
| gene22382 | 2.29E-03 | 0.628 | up | gene15754 | 1.32E-15 | 1.318 | up |
| gene17661 | 1.70E-03 | 0.628 | up | gene22390 | 3.16E-13 | 1.326 | up |
| gene24913 | 1.05E-02 | 0.629 | up | gene6787 | 1.74E-04 | 1.333 | up |
| gene20684 | 2.19E-03 | 0.629 | up | gene29139 | 5.01E-18 | 1.341 | up |
| gene22913 | 5.62E-03 | 0.630 | up | gene7422 | 3.89E-03 | 1.342 | up |
| gene11755 | 3.80E-03 | 0.630 | up | gene20676 | 2.75E-15 | 1.346 | up |
| gene15566 | 2.40E-03 | 0.631 | up | gene26439 | 3.55E-14 | 1.348 | up |
| gene2803 | 1.58E-03 | 0.631 | up | gene10407 | 8.32E-11 | 1.349 | up |
| gene26660 | 1.41E-03 | 0.632 | up | gene22876 | 5.01E-13 | 1.364 | up |
| gene23268 | 5.37E-03 | 0.634 | up | Pig_newGene_43037 | 1.26E-20 | 1.365 | up |
| gene9717 | 3.72E-03 | 0.634 | up | gene24054 | 3.98E-15 | 1.377 | up |
| gene28820 | 1.38E-03 | 0.634 | up | gene11081 | 2.00E-18 | 1.399 | up |
| gene9736 | 6.46E-03 | 0.635 | up | gene24933 | 1.58E-19 | 1.400 | up |
| gene23257 | 1.45E-03 | 0.635 | up | gene23047 | 3.89E-03 | 1.405 | up |
| gene18548 | 1.62E-03 | 0.635 | up | gene4973 | 2.09E-12 | 1.412 | up |
| gene828 | 1.35E-03 | 0.636 | up | gene19047 | 1.32E-04 | 1.413 | up |
| gene24943 | 1.38E-03 | 0.636 | up | gene6022 | 1.02E-13 | 1.416 | up |
| gene23950 | 3.39E-02 | 0.636 | up | gene11451 | 1.26E-20 | 1.419 | up |
| gene21063 | 5.62E-03 | 0.637 | up | gene12120 | 3.24E-09 | 1.422 | up |
| gene9588 | 1.17E-03 | 0.638 | up | gene29245 | 1.26E-13 | 1.440 | up |
| gene20678 | 1.23E-03 | 0.638 | up | gene26074 | 1.58E-19 | 1.460 | up |
| gene15201 | 1.07E-03 | 0.639 | up | gene4422 | 7.76E-06 | 1.468 | up |
| gene19767 | 1.26E-03 | 0.642 | up | Pig_newGene_79790 | 3.16E-17 | 1.476 | up |
| gene21254 | 1.29E-03 | 0.642 | up | gene7608 | 2.00E-18 | 1.480 | up |
| gene9389 | 2.40E-03 | 0.643 | up | gene1236 | 2.51E-19 | 1.495 | up |
| gene15628 | 1.05E-03 | 0.644 | up | gene4974 | 3.16E-20 | 1.520 | up |
| gene7298 | 1.82E-02 | 0.645 | up | gene9971 | 7.59E-09 | 1.553 | up |
| gene9868 | 9.33E-04 | 0.646 | up | gene6166 | 7.76E-10 | 1.586 | up |
| gene27739 | 1.58E-03 | 0.646 | up | gene20898 | 7.94E-11 | 1.587 | up |
| gene25047 | 1.00E-03 | 0.646 | up | gene301 | 1.26E-12 | 1.589 | up |
| gene3197 | 9.33E-04 | 0.647 | up | gene2785 | 1.58E-13 | 1.594 | up |
| gene17405 | 2.57E-03 | 0.647 | up | gene25066 | 2.00E-14 | 1.618 | up |
| gene21283 | 2.69E-03 | 0.647 | up | gene19716 | 1.91E-06 | 1.637 | up |
| gene20350 | 8.51E-04 | 0.647 | up | gene4977 | 1.45E-15 | 1.646 | up |
| gene27038 | 8.71E-04 | 0.649 | up | gene20917 | 2.51E-15 | 1.656 | up |
| gene4465 | 2.95E-03 | 0.649 | up | Pig_newGene_215232 | 3.16E-16 | 1.661 | up |
| gene1517 | 8.13E-04 | 0.649 | up | gene2027 | 2.69E-10 | 1.661 | up |
| gene21290 | 2.00E-03 | 0.649 | up | gene26548 | 3.16E-17 | 1.662 | up |
| gene22927 | 1.38E-03 | 0.650 | up | gene17184 | 3.98E-18 | 1.735 | up |
| gene25514 | 1.66E-03 | 0.650 | up | gene26859 | 5.01E-19 | 1.737 | up |
| gene4437 | 7.94E-04 | 0.650 | up | Pig_newGene_205147 | 6.31E-20 | 1.739 | up |
| gene6631 | 8.32E-04 | 0.651 | up | gene973 | 7.94E-21 | 1.787 | up |
| gene20487 | 9.12E-04 | 0.651 | up | gene2778 | 2.51E-07 | 1.806 | up |
| gene27124 | 3.39E-03 | 0.651 | up | gene26709 | 2.00E-08 | 1.812 | up |
| gene27690 | 7.24E-03 | 0.653 | up | gene6408 | 1.58E-09 | 1.847 | up |
| gene16667 | 6.76E-04 | 0.653 | up | gene11385 | 1.26E-10 | 1.952 | up |
| gene24042 | 7.94E-04 | 0.654 | up | gene29288 | 1.00E-10 | 2.013 | up |
| gene24383 | 1.15E-03 | 0.655 | up | gene6297 | 4.57E-15 | 2.026 | up |
| gene20463 | 1.26E-02 | 0.655 | up | Pig_newGene_141371 | 7.94E-12 | 2.035 | up |
| gene21666 | 6.61E-04 | 0.655 | up | gene8112 | 6.31E-13 | 2.110 | up |
| gene11374 | 6.76E-04 | 0.655 | up | gene8079 | 5.01E-14 | 2.146 | up |
| gene2230 | 1.41E-03 | 0.656 | up | gene27515 | 3.98E-15 | 2.175 | up |
| gene17357 | 6.61E-04 | 0.656 | up | gene6963 | 3.16E-16 | 2.200 | up |
| gene9919 | 6.92E-04 | 0.657 | up | gene19369 | 2.51E-17 | 2.246 | up |
| gene23735 | 7.94E-04 | 0.657 | up | gene1095 | 2.00E-18 | 2.250 | up |
| gene22222 | 6.31E-04 | 0.657 | up | gene11382 | 1.58E-19 | 2.263 | up |
| gene21945 | 6.31E-04 | 0.657 | up | gene14848 | 2.45E-09 | 2.268 | up |
| gene25048 | 6.03E-04 | 0.658 | up | gene4293 | 1.26E-20 | 2.279 | up |
| gene6623 | 6.76E-04 | 0.658 | up | gene320 | 1.45E-08 | 2.300 | up |
| gene978 | 1.48E-03 | 0.659 | up | Pig_newGene_211434 | 2.51E-08 | 2.363 | up |
| gene25237 | 5.50E-04 | 0.659 | up | gene17047 | 2.00E-09 | 2.364 | up |
| gene25475 | 5.62E-04 | 0.659 | up | gene5422 | 2.00E-10 | 2.388 | up |
| gene9747 | 5.50E-04 | 0.660 | up | gene19825 | 1.58E-10 | 2.407 | up |
| gene24948 | 5.62E-04 | 0.661 | up | gene3869 | 1.26E-11 | 2.469 | up |
| gene8540 | 3.02E-03 | 0.662 | up | gene10844 | 1.00E-11 | 2.494 | up |
| gene19217 | 6.17E-03 | 0.662 | up | gene23319 | 7.94E-13 | 2.500 | up |
| gene2231 | 9.55E-04 | 0.662 | up | gene30218 | 6.31E-14 | 2.529 | up |
| Pig_newGene_78748 | 1.23E-02 | 0.663 | up | gene3310 | 5.01E-15 | 2.557 | up |
| gene1042 | 1.32E-03 | 0.663 | up | Pig_newGene_133171 | 3.98E-16 | 2.563 | up |
| gene18521 | 4.90E-04 | 0.664 | up | gene25884 | 6.17E-13 | 2.633 | up |
| gene7167 | 2.04E-03 | 0.664 | up | gene9203 | 3.16E-17 | 2.727 | up |
| gene17669 | 5.13E-04 | 0.665 | up | gene13733 | 2.51E-18 | 2.802 | up |
| gene18534 | 5.62E-04 | 0.665 | up | gene12100 | 2.00E-18 | 2.953 | up |
| gene683 | 5.37E-04 | 0.665 | up | gene10646 | 3.80E-14 | 3.095 | up |
| gene16600 | 7.24E-04 | 0.666 | up | gene22736 | 2.00E-19 | 3.128 | up |
| gene5682 | 1.70E-02 | 0.666 | up | Pig_newGene_243383 | 1.12E-15 | 3.271 | up |
| gene4501 | 9.33E-03 | 0.667 | up | gene20047 | 1.58E-20 | 3.424 | up |
| gene21672 | 6.17E-04 | 0.667 | up | gene28868 | 1.26E-21 | 3.753 | up |
| gene15498 | 4.07E-04 | 0.668 | up | gene4988 | 1.26E-11 | 3.860 | up |
| gene10004 | 5.01E-04 | 0.669 | up | gene19989 | 7.94E-23 | 4.418 | up |
| gene4625 | 4.27E-04 | 0.669 | up | gene11946 | 6.31E-24 | 4.605 | up |
| Pig_newGene_83610 | 2.29E-03 | 0.669 | up | gene26025 | 5.01E-25 | 4.935 | up |
| gene4677 | 3.98E-04 | 0.669 | up | Pig_newGene_206428 | 3.98E-26 | 5.176 | up |
| gene15518 | 2.45E-02 | 0.669 | up | gene13051 | 3.16E-27 | 5.223 | up |
| gene22966 | 5.13E-04 | 0.670 | up | gene8084 | 2.51E-28 | 5.276 | up |
| Pig_newGene_203850 | 4.47E-04 | 0.670 | up | Pig_newGene_194856 | 2.00E-29 | 5.367 | up |
| gene23584 | 3.89E-04 | 0.670 | up | Pig_newGene_192505 | 1.58E-30 | 5.773 | up |
| gene9719 | 3.89E-04 | 0.670 | up | gene21112 | 1.26E-23 | 6.213 | up |
| gene3301 | 4.17E-04 | 0.671 | up | gene14415 | 1.26E-23 | 6.219 | up |
| gene1811 | 3.98E-04 | 0.671 | up | Pig_newGene_209873 | 1.00E-30 | 6.262 | up |
| gene26250 | 5.89E-04 | 0.671 | up | gene8105 | 7.94E-33 | 8.146 | up |
|  |  |  |  | gene16625 | 5.01E-34 | 8.828 | up |

|Log_2_(Fold change)| ≥ 0.59 and adjusted FDR < 0.05 was the cut-off criteria for DEmRNAs.

**TABLE S3** GO enrichment analysis of DEmRNAs after *NORFA* inhibition.

| **Category** | **Term ID** | **GO Terms** | **Count** | **Percentage %** | ***P*-value^1^** |
| --- | --- | --- | --- | --- | --- |
| GOTERM_BP | GO:0045944 | positive regulation of transcription from RNA polymerase II promoter | 36 | 3.7306 | 2.80E-03 |
| GOTERM_BP | GO:0006355 | regulation of transcription, DNA-templated | 27 | 2.7979 | 4.63E-02 |
| GOTERM_BP | GO:0000122 | negative regulation of transcription from RNA polymerase II promoter | 24 | 2.4870 | 3.70E-02 |
| GOTERM_BP | GO:0006351 | transcription, DNA-templated | 23 | 2.3834 | 2.83E-02 |
| GOTERM_BP | GO:0045892 | negative regulation of transcription, DNA-templated | 19 | 1.9689 | 5.40E-03 |
| GOTERM_BP | GO:0007018 | microtubule-based movement | 10 | 1.0363 | 2.81E-04 |
| GOTERM_BP | GO:0043524 | negative regulation of neuron apoptotic process | 9 | 0.9326 | 1.77E-02 |
| GOTERM_BP | GO:0045444 | fat cell differentiation | 7 | 0.7254 | 1.77E-02 |
| GOTERM_BP | GO:0051726 | regulation of cell cycle | 7 | 0.7254 | 5.96E-02 |
| GOTERM_BP | GO:0097192 | extrinsic apoptotic signaling pathway in absence of ligand | 6 | 0.6218 | 1.83E-03 |
| GOTERM_BP | GO:0008630 | intrinsic apoptotic signaling pathway in response to DNA damage | 6 | 0.6218 | 6.57E-03 |
| GOTERM_BP | GO:0019827 | stem cell population maintenance | 6 | 0.6218 | 2.40E-02 |
| GOTERM_BP | GO:0050680 | negative regulation of epithelial cell proliferation | 6 | 0.6218 | 2.40E-02 |
| GOTERM_BP | GO:0051897 | positive regulation of protein kinase B signaling | 6 | 0.6218 | 4.89E-02 |
| GOTERM_BP | GO:0007050 | cell cycle arrest | 6 | 0.6218 | 4.35E-02 |
| GOTERM_BP | GO:0009791 | post-embryonic development | 6 | 0.6218 | 4.79E-02 |
| GOTERM_BP | GO:0000042 | protein targeting to Golgi | 5 | 0.5181 | 5.94E-04 |
| GOTERM_BP | GO:0032956 | regulation of actin cytoskeleton organization | 5 | 0.5181 | 1.77E-02 |
| GOTERM_BP | GO:0071407 | cellular response to organic cyclic compound | 5 | 0.5181 | 2.79E-02 |
| GOTERM_BP | GO:0001933 | negative regulation of protein phosphorylation | 5 | 0.5181 | 3.20E-02 |
| GOTERM_BP | GO:0070373 | negative regulation of ERK1 and ERK2 cascade | 5 | 0.5181 | 4.83E-02 |
| GOTERM_BP | GO:0019050 | suppression by virus of host apoptotic process | 5 | 0.2073 | 4.58E-02 |
| GOTERM_BP | GO:2001241 | positive regulation of extrinsic apoptotic pathway in absence of ligand | 5 | 0.3109 | 4.99E-02 |
| GOTERM_BP | GO:0001836 | release of cytochrome c from mitochondria | 4 | 0.4145 | 1.44E-02 |
| GOTERM_BP | GO:0008053 | mitochondrial fusion | 4 | 0.4145 | 1.86E-02 |
| GOTERM_BP | GO:2000114 | regulation of establishment of cell polarity | 4 | 0.4145 | 2.33E-02 |
| GOTERM_BP | GO:0051402 | neuron apoptotic process | 4 | 0.4145 | 3.44E-02 |
| GOTERM_BP | GO:0045786 | negative regulation of cell cycle | 4 | 0.4145 | 3.44E-02 |
| GOTERM_BP | GO:2001243 | negative regulation of intrinsic apoptotic signaling pathway | 4 | 0.4145 | 4.79E-02 |
| GOTERM_BP | GO:0032534 | regulation of microvillus assembly | 3 | 0.3109 | 6.98E-03 |
| GOTERM_BP | GO:0051572 | negative regulation of histone H3-K4 methylation | 3 | 0.3109 | 1.35E-02 |
| GOTERM_BP | GO:0046902 | regulation of mitochondrial membrane permeability | 3 | 0.3109 | 1.35E-02 |
| GOTERM_BP | GO:0045616 | regulation of keratinocyte differentiation | 3 | 0.3109 | 1.35E-02 |
| GOTERM_BP | GO:0000722 | telomere maintenance via recombination | 3 | 0.3109 | 2.18E-02 |
| GOTERM_BP | GO:0060068 | vagina development | 3 | 0.3109 | 2.18E-02 |
| GOTERM_BP | GO:0008593 | regulation of Notch signaling pathway | 3 | 0.3109 | 2.18E-02 |
| GOTERM_BP | GO:0022407 | regulation of cell-cell adhesion | 3 | 0.3109 | 3.16E-02 |
| GOTERM_BP | GO:0006265 | DNA topological change | 3 | 0.3109 | 4.29E-02 |
| GOTERM_BP | GO:0032471 | negative regulation of endoplasmic reticulum calcium ion concentration | 3 | 0.3109 | 4.29E-02 |
| GOTERM_BP | GO:0043928 | mRNA catabolic process involved in deadenylation-dependent decay | 3 | 0.3109 | 4.29E-02 |
| GOTERM_BP | GO:0045778 | positive regulation of ossification | 3 | 0.3109 | 4.29E-02 |
| GOTERM_BP | GO:0060039 | pericardium development | 3 | 0.3109 | 4.29E-02 |
| GOTERM_BP | GO:0035067 | negative regulation of histone acetylation | 3 | 0.3109 | 4.55E-02 |
| GOTERM_BP | GO:0030522 | intracellular receptor signaling pathway | 3 | 0.3109 | 4.69E-02 |
| GOTERM_BP | GO:1902750 | negative regulation of cell cycle G2/M phase transition | 3 | 0.2073 | 4.96E-02 |
| GOTERM_CC | GO:0005737 | cytoplasm | 133 | 13.7824 | 1.14E-03 |
| GOTERM_CC | GO:0005634 | nucleus | 130 | 13.4715 | 1.32E-03 |
| GOTERM_CC | GO:0005654 | nucleoplasm | 72 | 7.4611 | 4.22E-05 |
| GOTERM_CC | GO:0005622 | intracellular | 52 | 5.3886 | 2.71E-05 |
| GOTERM_CC | GO:0016020 | membrane | 49 | 5.0777 | 9.59E-03 |
| GOTERM_CC | GO:0005730 | nucleolus | 43 | 4.4560 | 3.34E-05 |
| GOTERM_CC | GO:0005813 | centrosome | 22 | 2.2798 | 7.47E-03 |
| GOTERM_CC | GO:0000790 | nuclear chromatin | 11 | 1.1399 | 3.30E-02 |
| GOTERM_CC | GO:0031965 | nuclear membrane | 10 | 1.0363 | 3.88E-02 |
| GOTERM_CC | GO:0005871 | kinesin complex | 9 | 0.9326 | 3.70E-04 |
| GOTERM_CC | GO:0016607 | nuclear speck | 9 | 0.9326 | 2.53E-02 |
| GOTERM_CC | GO:0071013 | catalytic step 2 spliceosome | 8 | 0.8290 | 2.86E-02 |
| GOTERM_CC | GO:0000785 | chromatin | 7 | 0.7254 | 3.76E-02 |
| GOTERM_CC | GO:0000776 | kinetochore | 6 | 0.6218 | 3.09E-02 |
| GOTERM_CC | GO:0016363 | nuclear matrix | 6 | 0.6218 | 5.04E-02 |
| GOTERM_CC | GO:1990023 | mitotic spindle midzone | 4 | 0.4145 | 1.98E-03 |
| GOTERM_CC | GO:0000177 | cytoplasmic exosome (RNase complex) | 4 | 0.4145 | 1.03E-02 |
| GOTERM_CC | GO:0000176 | nuclear exosome (RNase complex) | 4 | 0.4145 | 1.76E-02 |
| GOTERM_CC | GO:0035098 | ESC/E(Z) complex | 4 | 0.4145 | 2.20E-02 |
| GOTERM_CC | GO:0000775 | chromosome, centromeric region | 4 | 0.4145 | 3.88E-02 |
| GOTERM_CC | GO:0097431 | mitotic spindle pole | 3 | 0.3109 | 1.30E-02 |
| GOTERM_CC | GO:0031519 | PcG protein complex | 3 | 0.3109 | 4.13E-02 |
| GOTERM_CC | GO:0097136 | Bcl-2 family protein complex | 2 | 0.2073 | 4.94E-02 |
| GOTERM_MF | GO:0005524 | ATP binding | 69 | 7.1503 | 2.57E-03 |
| GOTERM_MF | GO:0008270 | zinc ion binding | 61 | 6.3212 | 4.49E-04 |
| GOTERM_MF | GO:0044822 | poly(A) RNA binding | 57 | 5.9067 | 3.35E-05 |
| GOTERM_MF | GO:0046872 | metal ion binding | 57 | 5.9067 | 2.63E-03 |
| GOTERM_MF | GO:0003676 | nucleic acid binding | 50 | 5.1813 | 3.50E-08 |
| GOTERM_MF | GO:0003677 | DNA binding | 39 | 4.0415 | 1.62E-03 |
| GOTERM_MF | GO:0003700 | transcription factor activity, sequence-specific DNA binding | 36 | 3.7306 | 7.91E-04 |
| GOTERM_MF | GO:0003682 | chromatin binding | 23 | 2.3834 | 2.40E-03 |
| GOTERM_MF | GO:0005525 | GTP binding | 21 | 2.1762 | 4.55E-02 |
| GOTERM_MF | GO:0000978 | RNA polymerase II core promoter proximal region DNA binding | 16 | 1.6580 | 4.56E-02 |
| GOTERM_MF | GO:0003729 | mRNA binding | 12 | 1.2435 | 7.00E-04 |
| GOTERM_MF | GO:0004842 | ubiquitin-protein transferase activity | 11 | 1.1399 | 4.70E-02 |
| GOTERM_MF | GO:0016887 | ATPase activity | 9 | 0.9326 | 2.77E-02 |
| GOTERM_MF | GO:0044212 | transcription regulatory region DNA binding | 9 | 0.9326 | 4.81E-02 |
| GOTERM_MF | GO:0003777 | microtubule motor activity | 7 | 0.7254 | 1.75E-02 |
| GOTERM_MF | GO:0046982 | protein heterodimerization activity | 6 | 0.6218 | 4.03E-02 |
| GOTERM_MF | GO:0008574 | ATP-dependent microtubule motor activity, plus-end-directed | 5 | 0.5181 | 9.43E-04 |
| GOTERM_MF | GO:0008301 | DNA binding, bending | 4 | 0.4145 | 1.08E-02 |
| GOTERM_MF | GO:0016538 | cyclin-dependent protein serine/threonine kinase regulator activity | 3 | 0.3109 | 4.69E-02 |
| GOTERM_MF | GO:0008026 | ATP-dependent helicase activity | 3 | 0.3109 | 4.83E-02 |

^1^GO terms with *P*-value < 0.05 were considered as significant functional processes of DEmRNAs.

**TABLE S4** KEGG pathway analysis of DEmRNAs after knocking down of *NORFA*.

| **Term ID** | **KEGG Term** | **Count** | **Percentage %** | ***P*-value^1^** | **Fold.Enrichment** |
| --- | --- | --- | --- | --- | --- |
| ssc05200 | Pathways in cancer | 34 | 3.5233 | 1.52E-05 | 2.246 |
| ssc04144 | Endocytosis | 21 | 2.1762 | 8.79E-04 | 2.267 |
| ssc04151 | PI3K-Akt signaling pathway | 21 | 2.1762 | 3.12E-02 | 4.634 |
| ssc05166 | HTLV-I infection | 20 | 2.0725 | 3.94E-03 | 2.046 |
| ssc04810 | Regulation of actin cytoskeleton | 19 | 1.9689 | 6.56E-04 | 2.459 |
| ssc04071 | Sphingolipid signaling pathway | 17 | 1.7617 | 6.59E-06 | 3.850 |
| ssc05205 | Proteoglycans in cancer | 17 | 1.7617 | 3.17E-03 | 2.270 |
| ssc04010 | MAPK signaling pathway | 17 | 1.7617 | 3.02E-02 | 5.767 |
| ssc04022 | cGMP-PKG signaling pathway | 16 | 1.6580 | 1.31E-03 | 2.569 |
| ssc04014 | Ras signaling pathway | 16 | 1.6580 | 2.60E-02 | 3.845 |
| ssc05202 | Transcriptional misregulation in cancer | 15 | 1.5544 | 2.50E-03 | 2.503 |
| ssc05203 | Viral carcinogenesis | 15 | 1.5544 | 1.45E-02 | 2.046 |
| ssc04015 | Rap1 signaling pathway | 15 | 1.5544 | 3.17E-02 | 1.847 |
| ssc05206 | MicroRNAs in cancer | 15 | 1.5544 | 5.01E-02 | 1.669 |
| ssc04390 | Hippo signaling pathway | 14 | 1.4508 | 2.72E-03 | 2.592 |
| ssc04141 | Protein processing in endoplasmic reticulum | 14 | 1.4508 | 9.54E-03 | 2.233 |
| ssc04550 | Signaling regulating pluripotency of stem cells | 13 | 1.3472 | 4.90E-03 | 2.537 |
| ssc04611 | Platelet activation | 13 | 1.3472 | 4.90E-03 | 2.537 |
| ssc04120 | Ubiquitin mediated proteolysis | 13 | 1.3472 | 6.24E-03 | 4.461 |
| ssc05161 | Hepatitis B | 13 | 1.3472 | 1.54E-02 | 2.184 |
| ssc03013 | RNA transport | 13 | 1.3472 | 1.62E-02 | 2.169 |
| ssc04270 | Vascular smooth muscle contraction | 12 | 1.2435 | 5.69E-03 | 2.624 |
| ssc04261 | Adrenergic signaling in cardiomyocytes | 12 | 1.2435 | 1.30E-02 | 2.341 |
| ssc05210 | Colorectal cancer | 11 | 1.1399 | 2.87E-04 | 4.103 |
| ssc04114 | Oocyte meiosis | 11 | 1.1399 | 1.03E-02 | 5.560 |
| ssc04728 | Dopaminergic synapse | 11 | 1.1399 | 1.73E-02 | 2.365 |
| ssc04915 | Estrogen signaling pathway | 10 | 1.0363 | 1.28E-02 | 2.642 |
| ssc05145 | Toxoplasmosis | 10 | 1.0363 | 2.18E-02 | 2.416 |
| ssc05142 | Chagas disease (American trypanosomiasis) | 10 | 1.0363 | 2.44E-02 | 2.371 |
| ssc04931 | Insulin resistance | 10 | 1.0363 | 3.16E-02 | 2.265 |
| ssc04110 | Cell cycle | 10 | 1.0363 | 3.65E-02 | 2.206 |
| ssc04360 | Axon guidance | 10 | 1.0363 | 4.40E-02 | 2.132 |
| ssc03018 | RNA degradation | 9 | 0.9326 | 7.52E-03 | 3.127 |
| ssc04912 | GnRH signaling pathway | 9 | 0.9326 | 1.80E-02 | 2.686 |
| ssc05215 | Prostate cancer | 9 | 0.9326 | 1.80E-02 | 2.686 |
| ssc04914 | Progesterone-mediated oocyte maturation | 9 | 0.9326 | 2.05E-02 | 2.624 |
| ssc04723 | Retrograde endocannabinoid signaling | 9 | 0.9326 | 3.09E-02 | 2.429 |
| ssc04621 | NOD-like receptor signaling pathway | 7 | 0.7254 | 1.18E-02 | 3.624 |
| ssc04730 | Long-term depression | 7 | 0.7254 | 2.74E-02 | 3.010 |
| ssc05212 | Pancreatic cancer | 7 | 0.7254 | 3.64E-02 | 2.818 |
| ssc04210 | Apoptosis | 7 | 0.7254 | 5.03E-02 | 2.573 |
| ssc04350 | TGF-beta signaling pathway | 7 | 0.7254 | 4.81E-02 | 2.306 |
| ssc05213 | Endometrial cancer | 6 | 0.6218 | 4.21E-02 | 3.106 |

^1^*P*-value < 0.05 was considered as a cut-off criteria to identify significantly enriched pathways.

**TABLE S5** Hub genes in the protein-protein interaction network.

| **Gene ID** | **Gene name** | **Degree^1^** | **Radiality** | **Stress** | **Regulated** |
| --- | --- | --- | --- | --- | --- |
| gene15634 | *CENPE* | 36 | 0.60523870 | 320326 | up |
| gene23496 | *SMC3* | 32 | 0.60925222 | 220822 | up |
| gene3197 | *KIF18A* | 31 | 0.60713984 | 220510 | up |
| gene24044 | *PPP2CB* | 28 | 0.57672159 | 62634 | up |
| gene17424 | *CENPF* | 28 | 0.60608365 | 222156 | up |
| gene25102 | *KIF2A* | 27 | 0.53848754 | 124776 | up |
| gene16483 | *CUL5* | 26 | 0.57777778 | 72214 | up |
| gene20466 | *CASC5* | 26 | 0.59763414 | 124684 | up |
| gene23269 | *KIF11* | 26 | 0.58918462 | 115200 | up |
| gene24636 | *CUL3* | 23 | 0.59678918 | 126610 | up |
| gene24196 | *SGOL1* | 23 | 0.57313054 | 73046 | up |
| gene24063 | *HERC2* | 22 | 0.57313054 | 73046 | up |
| gene18874 | *SOCS3* | 22 | 0.59721166 | 120222 | down |
| gene188766 | *HSPCA* | 22 | 0.57439797 | 71234 | up |
| gene19131 | *CDC27* | 22 | 0.59509928 | 92002 | up |
| gene27783 | *STAG2* | 22 | 0.67460921 | 41532 | up |
| gene10392 | *FBXL8* | 21 | 0.66996198 | 61910 | down |
| gene6516 | *FBXO11* | 21 | 0.67946768 | 224174 | up |
| gene1135 | *ASB7* | 21 | 0.56151246 | 71242 | down |
| gene9592 | *SPSB2* | 21 | 0.54165610 | 21320 | down |
| gene1624 | *KLHL9* | 21 | 0.53954373 | 332786 | up |
| gene27373 | *CENPC1* | 21 | 0.54228982 | 22928 | up |
| gene6764 | *TOPOII* | 21 | 0.56932826 | 63890 | up |
| gene23974 | *CENPU* | 21 | 0.58200253 | 52042 | up |
| gene18313 | *PDS5B* | 21 | 0.59742290 | 82706 | up |

^1^DEmRNAs with degree ≥ 21 (top 5%) were considered as hub genes in the PPI network.

**TABLE S6** Differentially expressed miRNAs in *NORFA*-reduced porcine GCs.

| **miRNA ID** | **Mature Sequence (5'-3')** | **FDR** | **Log_2_FC** | **Regulated** |
| --- | --- | --- | --- | --- |
| novel-miR-318 | AGCCAGGUGGAUGCGGACGAGC | 4.91E-03 | -22.41 | down |
| novel-miR-122 | CAGGGCAUCCCUGUAGGAGCU | 1.15E-02 | -22.14 | down |
| novel-miR-118 | CCUCUGUUCACUCCCUGGAGCU | 2.67E-02 | -21.82 | down |
| novel-miR-263 | UGGAGGAGUCCUGGGCCUCUCUG | 3.92E-02 | -21.63 | down |
| novel-miR-74 | CGUGUGCUGCCCAGAGGCUGG | 1.88E-02 | -3.09 | down |
| ssc-miR-199b-5p | CCCAGUGUUUAGACUAUCUGUU | 3.36E-05 | -3.01 | down |
| novel-miR-155 | UGCAAUUGUGAUCUGAUCUCAGC | 1.33E-03 | -2.97 | down |
| novel-miR-77 | UAGUUGCUGUGGGAAGUGAGC | 4.83E-02 | -2.68 | down |
| ssc-miR-9851-3p | UGGCACCAGCACUGGCGGUGGC | 1.21E-02 | -2.51 | down |
| novel-miR-616 | UUGCAAGCAACACUCUGUGGCAGAU | 1.90E-03 | -2.51 | down |
| novel-miR-272 | CCACAGCCACAGCAACACC | 2.65E-03 | -2.45 | down |
| novel-miR-481 | AACGACACGGUCUCAUUUGUGG | 3.17E-02 | -2.21 | down |
| novel-miR-262 | UCGGGGAGGCUGUGCAGCGCGG | 1.06E-02 | -2.17 | down |
| novel-miR-162 | CCUGGGAACUUCCAUAUGCU | 1.42E-02 | -2.09 | down |
| novel-miR-253 | CGCCACAGCCACAGCAAUGCC | 1.42E-02 | -2.09 | down |
| novel-miR-347 | AUGUAGGGCUAAAAGCCAUGGG | 6.66E-03 | -2.03 | down |
| novel-miR-514 | AAGUGGGCAUGUGUGUGACAAAC | 1.94E-02 | -2.01 | down |
| novel-miR-164 | GGCUUGGAUCUGGUGUUGCU | 3.94E-03 | -1.94 | down |
| novel-miR-187 | UAGGGCACAGGAUGGGAUGAGGA | 2.57E-02 | -1.92 | down |
| novel-miR-302 | UGUGCUGUGCAGUAGGACCCAC | 2.57E-02 | -1.92 | down |
| novel-miR-364 | UACGAAUUUCAGGAAUACAGCA | 3.41E-02 | -1.83 | down |
| novel-miR-354 | UACGAAUUUCAGGAAUACAGCA | 3.41E-02 | -1.83 | down |
| novel-miR-362 | UCGUGGUCACUGUCGCUCUCCAGG | 1.57E-02 | -1.83 | down |
| novel-miR-448 | UGUGGCUGUGGCGUAUACCAGC | 2.11E-02 | -1.75 | down |
| ssc-miR-9858-5p | UUCCUGAGUCGGACUGGGCU | 6.11E-03 | -1.73 | down |
| novel-miR-397 | AGCCACGAUGGGAACUCC | 3.61E-03 | -1.70 | down |
| novel-miR-70 | CUCAGUGGGUUAAGGAUCUGA | 3.52E-02 | -1.59 | down |
| ssc-miR-451 | AAACCGUUACCAUUACUGAGUU | 3.74E-03 | -1.59 | down |
| novel-miR-466 | UAGUGGGUCGGGGAUCUGGUGUUGU | 4.28E-02 | -1.51 | down |
| novel-miR-110 | CUAAGGAUCUGGCAUUGCUGCA | 4.28E-02 | -1.51 | down |
| novel-miR-259 | CUUCUCUGUUACUUCCCUCAGG | 2.57E-03 | -1.44 | down |
| novel-miR-402 | GCGGCUGGCGGCCUGGACGCCGUG | 4.25E-02 | -1.36 | down |
| novel-miR-40 | UACAGUCAACGGUCGGUGGUUU | 7.31E-03 | -1.30 | down |
| novel-miR-416 | CACGUGCGUGCUGGUAUCUGG | 2.24E-02 | -1.26 | down |
| ssc-miR-345-3p | CCCUGAACUAGGGGUCUGGAG | 9.46E-06 | -1.25 | down |
| novel-miR-236 | CGCGGCAUAUGGAGGUUCCCAGGCC | 4.19E-02 | -1.24 | down |
| novel-miR-283 | AUCCAACUAGGAACCAUGAGG | 2.83E-03 | -1.21 | down |
| ssc-miR-1271 | UGCCUGCUAUGUGCCAGGCA | 2.67E-02 | -1.14 | down |
| ssc-miR-27a | UUCACAGUGGCUAAGUUCCGC | 4.73E-04 | -1.14 | down |
| novel-miR-615 | UGAGCUAUGACAGGAACGCCU | 8.73E-03 | -1.09 | down |
| novel-miR-328 | AUCCGGCAUUGCUGUGAGCUG | 1.09E-02 | -1.09 | down |
| novel-miR-323 | UGGAUCCUUAACCCACUGAGC | 1.38E-03 | -1.04 | down |
| novel-miR-25 | UGUGUCUGUGGCGUAGGCCAGA | 3.07E-02 | -1.00 | down |
| novel-miR-553 | AAGCAGGGACUGCUGAGGUUGG | 4.41E-02 | -0.98 | down |
| novel-miR-542 | CGCGGCAUAUGGAGGUUCCCAGGCU | 2.39E-02 | -0.98 | down |
| novel-miR-177 | GGCUUGGAUCUGGUGUUGCUG | 1.96E-03 | -0.95 | down |
| ssc-miR-9860-5p | UUGCCCGAGAGCUUGGACCGC | 4.26E-02 | -0.94 | down |
| novel-miR-471 | AGGAUCUGGUGUUGCUGUGAGCU | 1.66E-02 | -0.89 | down |
| ssc-miR-127 | UCGGAUCCGUCUGAGCUUGGCU | 8.58E-03 | -0.89 | down |
| novel-miR-216 | CAACACUGUGCUGGAAGAUGGA | 2.62E-02 | -0.89 | down |
| novel-miR-624 | UGUGGCUGUGGUGUAGGCUGGC | 3.98E-02 | -0.88 | down |
| ssc-miR-187 | UCGUGUCUUGUGUUGCAGCCGG | 1.99E-02 | -0.86 | down |
| novel-miR-47 | UCCCUGGCCCAGGAACUUCCUU | 5.21E-04 | -0.84 | down |
| novel-miR-340 | UCUCCUUGCGAGUCUCUGCUGC | 1.83E-02 | -0.83 | down |
| ssc-miR-139-5p | UCUACAGUGCACGUGUCUCCAG | 1.16E-02 | -0.83 | down |
| novel-miR-298 | CUUCCUGUCUCCUCCUUCCCAGU | 1.11E-02 | -0.82 | down |
| ssc-miR-126-3p | UCGUACCGUGAGUAAUAAUGCG | 4.62E-04 | -0.81 | down |
| novel-miR-168 | GUCACCUGGCAGGUACCUCUUU | 4.42E-04 | -0.81 | down |
| ssc-miR-7137-3p | AGCUGGUCUGGGAGUUCCCGGG | 7.25E-04 | -0.81 | down |
| novel-miR-169 | GCUGGAUCCUUAACCCACUGAGC | 1.21E-03 | -0.81 | down |
| novel-miR-303 | CGGGGGGUCGUGUGCCUGCGGU | 4.19E-02 | -0.79 | down |
| novel-miR-132 | AUGGGUCUGACGGAGCAGCCGC | 2.24E-03 | -0.79 | down |
| novel-miR-568 | UGAGUCAUGACGGGAACUCCUCA | 3.25E-04 | -0.77 | down |
| novel-miR-312 | UAAGUGCUUCCAUGUUUUAGU | 1.16E-02 | -0.77 | down |
| novel-miR-230 | UAACACUGUCUGGUAACGAUG | 4.72E-02 | -0.74 | down |
| novel-miR-185 | UAGGGGUCAGAUCAGAGCUGU | 3.72E-02 | -0.74 | down |
| ssc-miR-7135-5p | UCUCUGAGACACUGACUGUGG | 4.47E-02 | -0.73 | down |
| novel-miR-166 | AGGAUCUGGUGUUGCUGUGAGCU | 4.29E-02 | -0.72 | down |
| novel-miR-310 | UAAGUGCUUCCAUGUUUUAGU | 4.86E-05 | -0.71 | down |
| novel-miR-51 | CUGCCGUUGGGUCUGGGGUGUG | 1.57E-02 | -0.68 | down |
| novel-miR-247 | CACAGCCGGGCUGGCCUCUGGGCU | 4.82E-02 | -0.68 | down |
| ssc-miR-24-3p | UGGCUCAGUUCAGCAGGAACAG | 2.31E-05 | -0.67 | down |
| ssc-miR-339-3p | AGCUCCUCGAGGCCAGAGCCC | 4.27E-02 | -0.65 | down |
| novel-miR-130 | AGGAUCUGGCGUUGCCGUGAGC | 8.49E-03 | -0.65 | down |
| novel-miR-598 | UGAUUGGCACCUCUUUGAGUGA | 4.14E-02 | -0.64 | down |
| ssc-miR-424-3p | CAAAACGUGAGGCGCUGCUAU | 3.69E-02 | -0.63 | down |
| novel-miR-204 | UCUCCAGUGAGACAGUCUCUGG | 4.74E-02 | -0.62 | down |
| novel-miR-571 | GGGUUGAAUCGGAGCUGUAGC | 3.26E-02 | -0.61 | down |
| novel-miR-100 | UCAGGUAUAGCUGGAUCCAGGG | 9.61E-03 | -0.61 | down |
| novel-miR-53 | CUAAGGAUCUGGCAUUGCUGUG | 2.15E-02 | -0.61 | down |
| ssc-miR-450a | UUUUGCGAUGUGUUCCUAAUAU | 9.46E-06 | -0.60 | down |
| novel-miR-179 | GCUUCUACAGUGGCUGUGG | 3.92E-02 | -0.59 | down |
| ssc-miR-24-2-5p | GUGCCUACUGAGCUGAUAUCAGU | 2.32E-03 | -0.59 | down |
| novel-miR-559 | UGACAAUGAUGCACCUUCCUGAGG | 2.53E-02 | 0.59 | up |
| novel-miR-332 | AAUCACUAAUUCCACUGCCAUC | 4.48E-02 | 0.59 | up |
| novel-miR-26 | UGGAAACACUUCUGCACAAACUAG | 7.34E-03 | 0.65 | up |
| novel-miR-2 | GGCUGUGAUGUAGGCCUGAAGCUGC | 2.03E-03 | 0.68 | up |
| ssc-miR-542-3p | UGUGACAGAUUGAUAACUGAAA | 3.42E-03 | 0.70 | up |
| novel-miR-101 | UGGCAUUGCCGUGAGCUGUGGU | 3.90E-02 | 0.72 | up |
| novel-miR-425 | UGGAGCGCAGGGACCGGCCACC | 3.38E-02 | 0.79 | up |
| novel-miR-280 | AUCUGGGGUUGCUGUGAACUGU | 4.75E-02 | 0.82 | up |
| novel-miR-223 | UACGUCUGUGGUGUAGACCAGU | 7.76E-03 | 0.87 | up |
| novel-miR-352 | UAUUGGCCUACAGAAAUGACAGA | 4.27E-02 | 1.00 | up |
| novel-miR-545 | CAGUGGGUUGAGGAUCUGGAGUUGU | 2.48E-02 | 1.08 | up |
| novel-miR-382 | UGUGGAGACCUCAGCACGUAGU | 2.52E-03 | 1.26 | up |
| novel-miR-517 | ACCGGCCCGGGGCUGCCUCCA | 4.66E-02 | 1.27 | up |
| ssc-miR-193a-3p | AACUGGCCUACAAAGUCCCAGU | 4.66E-02 | 1.27 | up |
| novel-miR-205 | UAAUACUGCCGGGUAAUGAUGGA | 3.57E-02 | 1.39 | up |
| ssc-miR-122 | UGGAGUGUGACAAUGGUGUUUGU | 2.30E-05 | 1.51 | up |
| novel-miR-59 | GGGCAAGCCUGCGGAGGUGUGG | 4.46E-02 | 1.91 | up |
| novel-miR-591 | CUAUACAACUUACUACUUUCCC | 1.34E-02 | 2.08 | up |
| novel-miR-256 | UCCAGGGUGGAAAAGAGCUUG | 1.57E-02 | 2.66 | up |
| novel-miR-290 | GAUUGGGAACACUGGAGAACU | 9.12E-03 | 2.81 | up |
| novel-miR-108 | UUGGGGGACGAGAGGCUGUG | 3.70E-02 | 3.08 | up |
| novel-miR-619 | UAUGUGGGACGGUAAACCAUU | 1.17E-04 | 22.90 | up |

|Log_2_(Fold change)| ≥ 0.59 and adjusted FDR < 0.05 was the cut-off criteria for DEmiRNAs.

**TABLE S7** GO enrichment analysis of DEmiRNAs after *NORFA* inhibition.

| **Category** | **Term ID** | **GO term** | ***P*-value^1^** | **No. of genes** |
| --- | --- | --- | --- | --- |
| GO_BP | GO:0008150 | biological_process | 4.34E-13 | 3729 |
| GO_BP | GO:0034641 | cellular nitrogen compound metabolic process | 2.54E-91 | 1271 |
| GO_BP | GO:0009058 | biosynthetic process | 2.26E-55 | 1037 |
| GO_BP | GO:0006464 | cellular protein modification process | 6.57E-53 | 668 |
| GO_BP | GO:0006351 | transcription, DNA-templated | 9.65E-09 | 602 |
| GO_BP | GO:0044281 | small molecule metabolic process | 1.30E-19 | 548 |
| GO_BP | GO:0006950 | response to stress | 1.45E-07 | 492 |
| GO_BP | GO:0009056 | catabolic process | 2.02E-14 | 452 |
| GO_BP | GO:0002376 | immune system process | 1.84E-03 | 332 |
| GO_BP | GO:0022607 | cellular component assembly | 2.53E-14 | 323 |
| GO_BP | GO:0008219 | cell death | 2.34E-11 | 234 |
| GO_BP | GO:0016192 | vesicle-mediated transport | 1.39E-02 | 224 |
| GO_BP | GO:0065003 | macromolecular complex assembly | 1.26E-11 | 221 |
| GO_BP | GO:0045893 | positive regulation of transcription, DNA-templated | 3.51E-02 | 216 |
| GO_BP | GO:0034655 | nucleobase-containing compound catabolic process | 1.96E-07 | 204 |
| GO_BP | GO:0006461 | protein complex assembly | 2.23E-08 | 188 |
| GO_BP | GO:0007267 | cell-cell signaling | 1.26E-10 | 176 |
| GO_BP | GO:0042592 | homeostatic process | 1.85E-02 | 173 |
| GO_BP | GO:0010467 | gene expression | 1.32E-25 | 169 |
| GO_BP | GO:0045087 | innate immune response | 2.57E-03 | 158 |
| GO_BP | GO:0061024 | membrane organization | 5.65E-12 | 155 |
| GO_BP | GO:0008283 | cell proliferation | 1.39E-02 | 150 |
| GO_BP | GO:0007010 | cytoskeleton organization | 2.92E-02 | 146 |
| GO_BP | GO:0044403 | symbiosis, encompassing mutualism through parasitism | 1.83E-11 | 128 |
| GO_BP | GO:0048870 | cell motility | 4.21E-03 | 127 |
| GO_BP | GO:0007268 | synaptic transmission | 2.01E-12 | 125 |
| GO_BP | GO:0007411 | axon guidance | 2.52E-05 | 121 |
| GO_BP | GO:0007596 | blood coagulation | 5.24E-12 | 120 |
| GO_BP | GO:0016032 | viral process | 2.01E-12 | 118 |
| GO_BP | GO:0007399 | nervous system development | 2.69E-04 | 117 |
| GO_BP | GO:0044267 | cellular protein metabolic process | 1.34E-09 | 109 |
| GO_BP | GO:0048011 | neurotrophin TRK receptor signaling pathway | 2.66E-35 | 107 |
| GO_BP | GO:0000278 | mitotic cell cycle | 1.57E-09 | 97 |
| GO_BP | GO:0007173 | epidermal growth factor receptor signaling pathway | 9.46E-17 | 80 |
| GO_BP | GO:0038095 | Fc-epsilon receptor signaling pathway | 3.35E-28 | 72 |
| GO_BP | GO:0008543 | fibroblast growth factor receptor signaling pathway | 2.79E-11 | 68 |
| GO_BP | GO:0006367 | transcription initiation from RNA polymerase II promoter | 4.60E-07 | 67 |
| GO_BP | GO:0006790 | sulfur compound metabolic process | 4.83E-02 | 60 |
| GO_BP | GO:0043687 | post-translational protein modification | 3.50E-12 | 55 |
| GO_BP | GO:0030168 | platelet activation | 2.05E-06 | 55 |
| GO_BP | GO:0048015 | phosphatidylinositol-mediated signaling | 3.24E-10 | 52 |
| GO_BP | GO:0008286 | insulin receptor signaling pathway | 8.23E-05 | 50 |
| GO_BP | GO:0097190 | apoptotic signaling pathway | 1.88E-02 | 50 |
| GO_BP | GO:0044255 | cellular lipid metabolic process | 5.86E-10 | 48 |
| GO_BP | GO:0000086 | G2/M transition of mitotic cell cycle | 2.11E-04 | 42 |
| GO_BP | GO:0034330 | cell junction organization | 7.08E-04 | 41 |
| GO_BP | GO:0006644 | phospholipid metabolic process | 9.41E-03 | 41 |
| GO_BP | GO:0006325 | chromatin organization | 5.57E-05 | 39 |
| GO_BP | GO:0050900 | leukocyte migration | 3.15E-05 | 36 |
| GO_BP | GO:0002224 | toll-like receptor signaling pathway | 1.24E-04 | 32 |
| GO_BP | GO:0018279 | protein N-linked glycosylation via asparagine | 2.71E-05 | 31 |
| GO_BP | GO:0034142 | toll-like receptor 4 signaling pathway | 5.69E-05 | 29 |
| GO_BP | GO:0006112 | energy reserve metabolic process | 1.84E-04 | 29 |
| GO_BP | GO:0006928 | cellular component movement | 1.31E-02 | 29 |
| GO_BP | GO:0002755 | MyD88-dependent toll-like receptor signaling pathway | 9.17E-04 | 27 |
| GO_BP | GO:0046474 | glycerophospholipid biosynthetic process | 1.68E-04 | 26 |
| GO_BP | GO:0038123 | toll-like receptor TLR1:TLR2 signaling pathway | 2.41E-08 | 25 |
| GO_BP | GO:0038124 | toll-like receptor TLR6:TLR2 signaling pathway | 2.41E-08 | 25 |
| GO_BP | GO:0034134 | toll-like receptor 2 signaling pathway | 2.03E-05 | 25 |
| GO_BP | GO:0034138 | toll-like receptor 3 signaling pathway | 4.21E-05 | 25 |
| GO_BP | GO:0030203 | glycosaminoglycan metabolic process | 7.89E-03 | 25 |
| GO_BP | GO:0034166 | toll-like receptor 10 signaling pathway | 2.44E-08 | 24 |
| GO_BP | GO:0034146 | toll-like receptor 5 signaling pathway | 5.64E-07 | 24 |
| GO_BP | GO:0035666 | TRIF-dependent toll-like receptor signaling pathway | 9.74E-07 | 24 |
| GO_BP | GO:0034162 | toll-like receptor 9 signaling pathway | 4.54E-06 | 24 |
| GO_BP | GO:0002756 | MyD88-independent toll-like receptor signaling pathway | 1.13E-05 | 24 |
| GO_BP | GO:0006892 | post-Golgi vesicle-mediated transport | 6.61E-06 | 21 |
| GO_BP | GO:0038096 | Fc-gamma receptor signaling pathway involved in phagocytosis | 8.37E-05 | 21 |
| GO_BP | GO:0097193 | intrinsic apoptotic signaling pathway | 6.29E-03 | 21 |
| GO_BP | GO:0034329 | cell junction assembly | 2.43E-04 | 20 |
| GO_BP | GO:0006766 | vitamin metabolic process | 8.88E-03 | 19 |
| GO_BP | GO:0007202 | activation of phospholipase C activity | 9.97E-03 | 19 |
| GO_BP | GO:0030879 | mammary gland development | 3.82E-02 | 18 |
| GO_BP | GO:0051403 | stress-activated MAPK cascade | 1.93E-03 | 17 |
| GO_BP | GO:0055086 | nucleobase-containing small molecule metabolic process | 1.27E-02 | 17 |
| GO_BP | GO:0006767 | water-soluble vitamin metabolic process | 1.40E-02 | 17 |
| GO_BP | GO:0002576 | platelet degranulation | 4.19E-02 | 17 |
| GO_BP | GO:0043647 | inositol phosphate metabolic process | 2.46E-03 | 14 |
| GO_BP | GO:0006369 | termination of RNA polymerase II transcription | 6.61E-03 | 14 |
| GO_BP | GO:0031124 | mRNA 3'-end processing | 7.20E-03 | 13 |
| GO_BP | GO:0006921 | cellular component disassembly involved in execution phase of apoptosis | 1.88E-02 | 12 |
| GO_BP | GO:1900740 | positive regulation of protein insertion into mitochondrial membrane involved in apoptotic signaling pathway | 3.83E-03 | 11 |
| GO_BP | GO:0014047 | glutamate secretion | 6.29E-03 | 11 |
| GO_BP | GO:0035872 | nucleotide-binding domain, leucine rich repeat containing receptor signaling pathway | 3.70E-02 | 11 |
| GO_BP | GO:0007215 | glutamate receptor signaling pathway | 4.96E-03 | 9 |
| GO_BP | GO:0060397 | JAK-STAT cascade involved in growth hormone signaling pathway | 1.88E-02 | 9 |
| GO_BP | GO:0050690 | regulation of defense response to virus by virus | 1.88E-02 | 9 |
| GO_BP | GO:0070423 | nucleotide-binding oligomerization domain containing signaling pathway | 2.26E-02 | 8 |
| GO_CC | GO:0043226 | organelle | 2.15E-156 | 2505 |
| GO_CC | GO:0005575 | cellular_component | 6.97E-23 | 3893 |
| GO_CC | GO:0043234 | protein complex | 8.27E-18 | 893 |
| GO_CC | GO:0005654 | nucleoplasm | 2.62E-15 | 308 |
| GO_CC | GO:0005829 | cytosol | 2.70E-13 | 645 |
| GO_MF | GO:0003674 | molecular_function | 5.71E-34 | 3899 |
| GO_MF | GO:0043167 | ion binding | 1.49E-96 | 1591 |
| GO_MF | GO:0003723 | RNA binding | 2.29E-04 | 408 |
| GO_MF | GO:0019899 | enzyme binding | 6.13E-24 | 360 |
| GO_MF | GO:0001071 | nucleic acid binding transcription factor activity | 3.05E-22 | 283 |
| GO_MF | GO:0022857 | transmembrane transporter activity | 1.95E-02 | 227 |
| GO_MF | GO:0030234 | enzyme regulator activity | 5.99E-11 | 217 |
| GO_MF | GO:0008092 | cytoskeletal protein binding | 1.90E-12 | 208 |
| GO_MF | GO:0000988 | protein binding transcription factor activity | 6.89E-18 | 150 |
| GO_MF | GO:0032182 | small conjugating protein binding | 1.55E-03 | 30 |

^1^GO terms with *P*-value < 0.05 were considered as significant functional processes of DEmiRNAs.

**TABLE S8** KEGG pathway analysis of DEmiRNAs in *NORFA*-reduced porcine GCs.

| **KEGG pathway terms** | ***P*-value^1^** | **No. of targets** | **No. of DEmiRNAs** |
| --- | --- | --- | --- |
| Prion diseases | 2.92E-08 | 8 | 6 |
| Hippo signaling pathway | 9.46E-05 | 52 | 14 |
| Proteoglycans in cancer | 9.64E-05 | 69 | 15 |
| Pathways in cancer | 8.37E-04 | 140 | 16 |
| FoxO signaling pathway | 9.65E-04 | 57 | 15 |
| Glioma | 1.71E-03 | 27 | 13 |
| Biosynthesis of unsaturated fatty acids | 1.87E-03 | 7 | 5 |
| TGF-beta signaling pathway | 1.99E-03 | 32 | 13 |
| Hepatitis B | 3.43E-03 | 52 | 14 |
| Circadian rhythm | 4.56E-03 | 17 | 8 |
| GABAergic synapse | 4.56E-03 | 32 | 11 |
| Dorso-ventral axis formation | 4.87E-03 | 16 | 11 |
| Colorectal cancer | 4.87E-03 | 29 | 12 |
| Long-term potentiation | 4.87E-03 | 31 | 13 |
| Glutamatergic synapse | 4.93E-03 | 44 | 13 |
| Prolactin signaling pathway | 6.39E-03 | 30 | 12 |
| MAPK signaling pathway | 6.95E-03 | 93 | 16 |
| Signaling pathways regulating pluripotency | 7.27E-03 | 53 | 14 |
| Adrenergic signaling in cardiomyocytes | 8.72E-03 | 51 | 13 |
| Prostate cancer | 1.11E-02 | 35 | 14 |
| Acute myeloid leukemia | 1.29E-02 | 25 | 12 |
| Alanine, aspartate and glutamate metabolism | 1.50E-02 | 16 | 10 |
| Nicotine addiction | 1.50E-02 | 16 | 12 |
| Oxytocin signaling pathway | 1.50E-02 | 55 | 15 |
| Biotin metabolism | 1.64E-02 | 1 | 1 |
| Pancreatic cancer | 1.72E-02 | 27 | 11 |
| Endometrial cancer | 1.72E-02 | 24 | 11 |
| cAMP signaling pathway | 1.72E-02 | 71 | 15 |
| Wnt signaling pathway | 1.80E-02 | 48 | 15 |
| Sphingolipid signaling pathway | 1.84E-02 | 42 | 13 |
| Ras signaling pathway | 1.84E-02 | 75 | 14 |
| PI3K-Akt signaling pathway | 1.84E-02 | 109 | 17 |
| Non-small cell lung cancer | 2.40E-02 | 22 | 12 |
| Long-term depression | 2.40E-02 | 26 | 13 |
| ErbB signaling pathway | 2.40E-02 | 34 | 14 |
| Melanoma | 2.86E-02 | 28 | 14 |
| Endocrine and regulated calcium reabsorption | 2.90E-02 | 19 | 11 |
| Neurotrophin signaling pathway | 2.90E-02 | 44 | 16 |
| Small cell lung cancer | 3.07E-02 | 33 | 15 |
| Thyroid cancer | 3.20E-02 | 12 | 7 |
| Endocytosis | 3.20E-02 | 70 | 16 |
| Transcriptional misregulation in cancer | 4.15E-02 | 60 | 13 |
| Retrograde endocannabinoid signaling | 4.19E-02 | 40 | 14 |
| Amphetamine addiction | 4.73E-02 | 24 | 13 |
| Dopaminergic synapse | 4.82E-02 | 49 | 15 |

^1^*P*-value < 0.05 was considered as a criteria for significant enriched pathway identification.**TABLE S9** Hub genes and miRNAs in the miRNA-mRNA interaction network.

| **DEmiRNAs** | **Regulated** | **Common DEmRNA targets** | **Numbers** |
| --- | --- | --- | --- |
| ssc-miR-1271 | down | *RPAP3, NCKAP1, EVI5, KHSRP, DDX24, SPIN1,*  *CRK, DCUN1D3* | 8 |
| ssc-miR-187 | down | *PHLPP2, MME* | 2 |
| ssc-miR-27a | down | *USP1, RUNX2, PEAK1, CDKN3, CCDC82, ATM, TGFB2, BRCA2, BRCA1, PTEN, LNPK, DROSHA, AR, XIAP, KRAS* | 15 |
| ssc-miR-302 | down | *GATA4, CDC23, SRRM2* | 3 |
| ssc-miR-339 | down | *OIP5, IRGQ, LOC110261162, LOC110258368* | 4 |
| ssc-miR-345 | down | *ZNF827, FYCO1, PR2X6, KIF20B* | 4 |
| ssc-miR-7137 | down | *SPEN, ROCK1, DST, RALGAPA1, INTU, FGF2, AFF1, KIF26B, CSPG5, TMCC1, HEG1, LOC110256366, CLIP1, NOM1, ZNF862, LOC100519997, AKAP17B, CASP8AP2, TRRAP, LGALSL, EPPK1* | 21 |
| ssc-miR-9858 | down | *EP400, NCOA7* | 2 |
| ssc-miR-108 | up | *FKRP, ADAMTS13, B2M* | 3 |
| ssc-miR-126 | up | *TGFB1, ATF5, COL9A2, BAK1, FGF11, TTLL3, HDAC11, TRAF6, VGF, HBQ1* | 10 |
| ssc-miR-280 | up | *AKAP8, EFNA2* | 2 |
| ssc-miR-425 | up | *FBXL8, NAPG, MYLK4, ISLR2, FAM53A, SLC27A3, LOC110255270, LOC110256914, TAZ , NCLN, THNSL2* | 11 |
| ssc-miR-517 | up | *ZNF865, ZNF444, LOC100737142, VWA1, RGL2, LOC110255332, CTSC, TTC36, RGS9, ANKS6, SOCS7, GIT1, GAS7, FAM83G, ZMYND10, PHF19, UMODL1, PLBD2, SEC14L2, RTL10, LOC100511352, PTPN18, HOXD3, UBE2QL1, CENPB, GMEB2, CBX3, SHROOM4, APLN, ZFP92, PNCK, STS, FARSA, ADAMTS10, ZNF346, LOC110259518, C3H7orf50, ZNF668, SNX3, NOXO1, FAM110B, IGFBP6, WNT5B* | 43 |
| ssc-miR-545 | up | *MYCBPAP, MIEF2, ADAM8, GDAP1L1, GIGYF1, TMEM8A, FRMD5* | 7 |
| ssc-miR-59 | up | *LOC110261324, RPS6KL1, TRIM32, UBN2, CYP4F55, DENND6B, IFFO1* | 7 |

**TABLE S10** Hub TFs and miRNAs in the DETF-DEmiRNA interaction network.

| **DETFs** | **Regulated** | **Common DEmiRNA targets^1^** | **Numbers** |
| --- | --- | --- | --- |
| ATF4 | down | miR-542, miR-450a | 2 |
| HIC2 | down | miR-24-2, miR-139, miR-339, miR-199b, miR-9860,  miR-187, miR-9851, miR-542, miR-126, miR-345,  miR-451, miR-450a, miR-7137, miR-127, miR-27a,  miR-122, miR-24, miR-1271, miR-7135 | 19 |
| HOXD3 | down | miR-24-2, miR-187, miR-9851, miR-542, miR-345,  miR-451, miR-450a, miR-7137, miR-27a, miR-122,  miR-24, miR-1271, miR-7135 | 13 |
| NFIX | down | miR-24-2, miR-139, miR-339, miR-199b, miR-9860,  miR-187, miR-9851, miR-542, miR-126, miR-345,  miR-451, miR-450a, miR-7137, miR-127, miR-27a,  miR-122, miR-24, miR-1271, miR-7135 | 19 |
| NR5A1 | down | miR-24-2, miR-139, miR-339, miR-199b, miR-187,  miR-9851, miR-542, miR-345, miR-451, miR-450a,  miR-7137, miR-127, miR-27a, miR-122, miR-24,  miR-1271, miR-7135 | 17 |
| SOX13 | down | miR-24-2, miR-139, miR-339, miR-199b, miR-187,  miR-7137, miR-122 | 7 |
| AR | up | miR-24-2, miR-339, miR-9860, miR-450a, miR-7137,  miR-1271, miR-126 | 7 |
| GATA4 | up | miR-24-2, miR-339, miR-9851, miR-542, miR-7137,  miR-7135 | 6 |
| NFIB | up | miR-9860 | 1 |
| NFIC | up | miR-24-2, miR-139, miR-339, miR-199b, miR-9860,  miR-187, miR-9851, miR-542, miR-126, miR-345,  miR-451, miR-450a, miR-7137, miR-127, miR-27a,  miR-122, miR-24, miR-193a, miR-1271, miR-7135 | 20 |
| RUNX2 | up | miR-24-2, miR-139, miR-339, miR-199b, miR-9851,  miR-542, miR-126, miR-345, miR-451, miR-450a,  miR-7137, miR-127, miR-27a, miR-122,miR-24,  miR-1271, miR-7135 | 17 |
| SOX5 | up | miR-339, miR-9860, miR-187, miR-542, miR-451,  miR-450a, miR-7137, miR-122, miR-1271, miR-7135 | 10 |
| TCF7L2 | up | miR-24-2, miR-139, miR-339, miR-199b, miR-9860,  miR-187, miR-9851, miR-542, miR-345, miR-451,  miR-450a, miR-27a, miR-7135 | 13 |
